# Metformin-induced ROS upregulation as amplified by apigenin causes profound anticancer activity while sparing normal cells

**DOI:** 10.1038/s41598-021-93270-0

**Published:** 2021-07-07

**Authors:** Madhuri Shende Warkad, Chea-Ha Kim, Beom-Goo Kang, Soo-Hyun Park, Jun-Sub Jung, Jing-Hui Feng, Gozde Inci, Sung-Chan Kim, Hong-Won Suh, Soon Sung Lim, Jae-Yong Lee

**Affiliations:** 1 Department of Biochemistry, Institute of Cell Differentiation and Aging, College of Medicine, Hallym University, 1 Hallymdeahak-gil, Chuncheon, 24252 Republic of Korea (South Korea); 2 Department of Pharmacology, Institute of Natural Medicine, Hallym University, 1 Hallymdeahak-gil, Chuncheon, 24252 Republic of Korea (South Korea); 3 Department of Food Science and Nutrition, The Korean Institute of Nutrition, College of Natural Science , Hallym University, 1 Hallymdeahak-gil, Chuncheon, 24252 Republic of Korea (South Korea); 4 FrontBio Inc. #301, Bio-2, Chuncheon BioTown, 32 Soyanggang-ro, Chuncheon-si, Gangwon-do 24232 Republic of Korea (South Korea)

**Keywords:** Cancer, Oncology

## Abstract

Metformin increased cellular ROS levels in AsPC-1 pancreatic cancer cells, with minimal effect in HDF, human primary dermal fibroblasts. Metformin reduced cellular ATP levels in HDF, but not in AsPC-1 cells. Metformin increased AMPK, p-AMPK (Thr172), FOXO3a, p-FOXO3a (Ser413), and MnSOD levels in HDF, but not in AsPC-1 cells. p-AMPK and p-FOXO3a also translocated from the cytosol to the nucleus by metformin in HDF, but not in AsPC-1 cells. Transfection of si-FOXO3a in HDF increased ROS levels, while wt-FOXO3a-transfected AsPC-1 cells decreased ROS levels. Metformin combined with apigenin increased ROS levels dramatically and decreased cell viability in various cancer cells including AsPC-1 cells, with each drug used singly having a minimal effect. Metformin/apigenin combination synergistically decreased mitochondrial membrane potential in AsPC-1 cells but to a lesser extent in HDF cells. Metformin/apigenin combination in AsPC-1 cells increased DNA damage-, apoptosis-, autophagy- and necroptosis-related factors, but not in HDF cells. Oral administration with metformin/apigenin caused dramatic blocks tumor size in AsPC-1-xenografted nude mice. Our results suggest that metformin in cancer cells differentially regulates cellular ROS levels via AMPK-FOXO3a-MnSOD pathway and combination of metformin/apigenin exerts anticancer activity through DNA damage-induced apoptosis, autophagy and necroptosis by cancer cell-specific ROS amplification.

## Introduction

Reactive oxygen species (ROS) constitute a group of highly reactive molecules such as superoxide anion and hydrogen peroxide (H_2_O_2_), generated by mitochondrial byproducts of aerobic respiration, enzymatic activation of cytochrome p450^1^, and NADPH oxidases. ROS are thought to cause damage to the entire cell including its mitochondria and the nucleus by structurally harming DNA, protein, and lipids. However, ROS are also found to be required in certain cell processes as they are involved in maintenance of redox homeostasis and various cellular signaling pathways. Proper levels of ROS are also required for other cellular functions, including gene expression^2^.

For tumor cells, the basal production of ROS is slightly elevated; this is a consequence of increased rates of metabolism and differences in the metabolic pathways used compared with non-transformed cells. Increased ROS production in cancer cells is in the background of increased levels of gene mutation and relative hypoxia compared with normal cells. Moderate increases in ROS levels in cancer cells are thought to contribute to tumor promotion and progression, as they are involved in signaling and metabolic pathways and they enable DNA mutation; however, as higher levels of ROS can induce apoptosis, autophagy and necroptosis in the same cells, these cells also remove higher levels of ROS by increasing the activity of their antioxidant pathways^3–5^. Modulating ROS levels could be a useful therapeutic strategy for treating cancer^6^; for example, ROS inducers such as doxorubicin and cisplatin have proven to be effective anticancer drugs. Most such drugs, however, induce ROS not only in cancer cells but also in non-transformed cells, making their use as anticancer drugs less appealing as they increase peripheral side effects and toxicity.

Metformin, a biguanide, is a prescribed drug for type 2 diabetes patients. It interacts with the respiratory complex I of the electron transport chain (ETC) in mitochondria and makes a mild leakage of electron transport to cause ROS production, leading to a mild reduction in ATP production. ATP reduction, in general, results in an activation of AMP-activated protein kinase (AMPK), in turn inhibiting the mammalian target of the rapamycin (mTOR) pathway and translating to a reduction in cell proliferation. Over-inhibition of mTOR pathway can also induce apoptosis and cell-cycle arrest in the cell^7,8^. Increased AMPK activity has also been reported to activate the forkhead box O3a (FOXO3a, or FKHRL1) transcription factor via phosphorylation^9^. FOXO3a belongs to the FOXO family of transcription factors which play a crucial role in regulating cell cycle arrest, cell death, ROS detoxification, metabolism and longevity^10,11^. FOXO3a protein mediates resistance to oxidative stress via transcriptional activation of the ROS removal enzymes such as catalase and MnSOD^10,12,13^.

Differences between normal and cancer cells may involve alternate modes of energy metabolism with different yields of ATP produced from the glucose consumed by each cell type. In normal cells, in the presence of optimal levels of oxygen, glucose is completely oxidized to CO_2_, generating 30 or 32 mol of ATP per mole of glucose consumed; this process involves sequential TCA cycle and oxidative phosphorylation in mitochondria^14^. When oxygen is limiting, pyruvate is metabolized to lactate instead. For cancer cells, even in the presence of optimal levels of oxygen, glucose is mostly fermented to lactate, generating 2 mol of ATP per mole of glucose, a behavior coined “the Warburg effect.” As oxidative phosphorylation in the mitochondria of cancer cells is heavily downregulated^15^, the secretion of lactate due to the Warburg effect is thought to facilitate tumor progression^16^.

Metformin has been known to possess anti-cancer properties^17,18^. In addition, metformin combined with other anticancer drugs such as doxorubicin^19^, trametinib^20^, and cisplatin^21^ t exerts a strong anticancer effect as revealed in multiple xenograft animal models. However, those combination therapies still show serious toxic effects. Although metformin is considered as a candidate for cancer therapy, metformin by itself is not currently used in cancer patients. The current study sought to assess the anticancer activity of metformin in vitro and in vivo when it was combined with an ROS amplifier, namely apigenin. Apigenin (4′,5,7-trihydroxy-flavone) belongs to the class of ROS amplifiers^22^, a group of agents that increase intracellular ROS. Besides being an anti-oxidant, apigenin also exhibits antibacterial, antiviral, and anti-inflammatory effects^23^. In normal cells, apigenin upregulates anti-oxidant enzymes. However, in cancer cells, apigenin exhibits pro-oxidation properties^24,25^, and several lines of evidence have shown apigenin possesses anticancer activity^26,27^. Apigenin enhances anticancer activity when it is co-administered with typical anticancer drugs such as gemcitabine^28^ and abivertinib^29^ both in vitro and in vivo.

Primary purpose of this study was to investigate whether the combination of metformin and apigenin can shows a synergistic anticancer activity via a cancer cell-specific targeting. To achieve this aim, we carried out the experiment if metformin exerts differential effects on ROS targeting-related pathway between the normal and cancer cells. In addition, the possible synergistic effect of metformin and apigenin in the regulation of cell growth inhibition, ROS amplification, and mitochondrial membrane potential inhibition, and cell death in cancer cells. Furthermore, the effect of combination of metformin and apigenin on cancer tumor size in xenografted nude mice was examined.

## Results

### Metformin differentially regulates cellular ROS and ATP levels in normal and cancer cells

**Figure 1:**
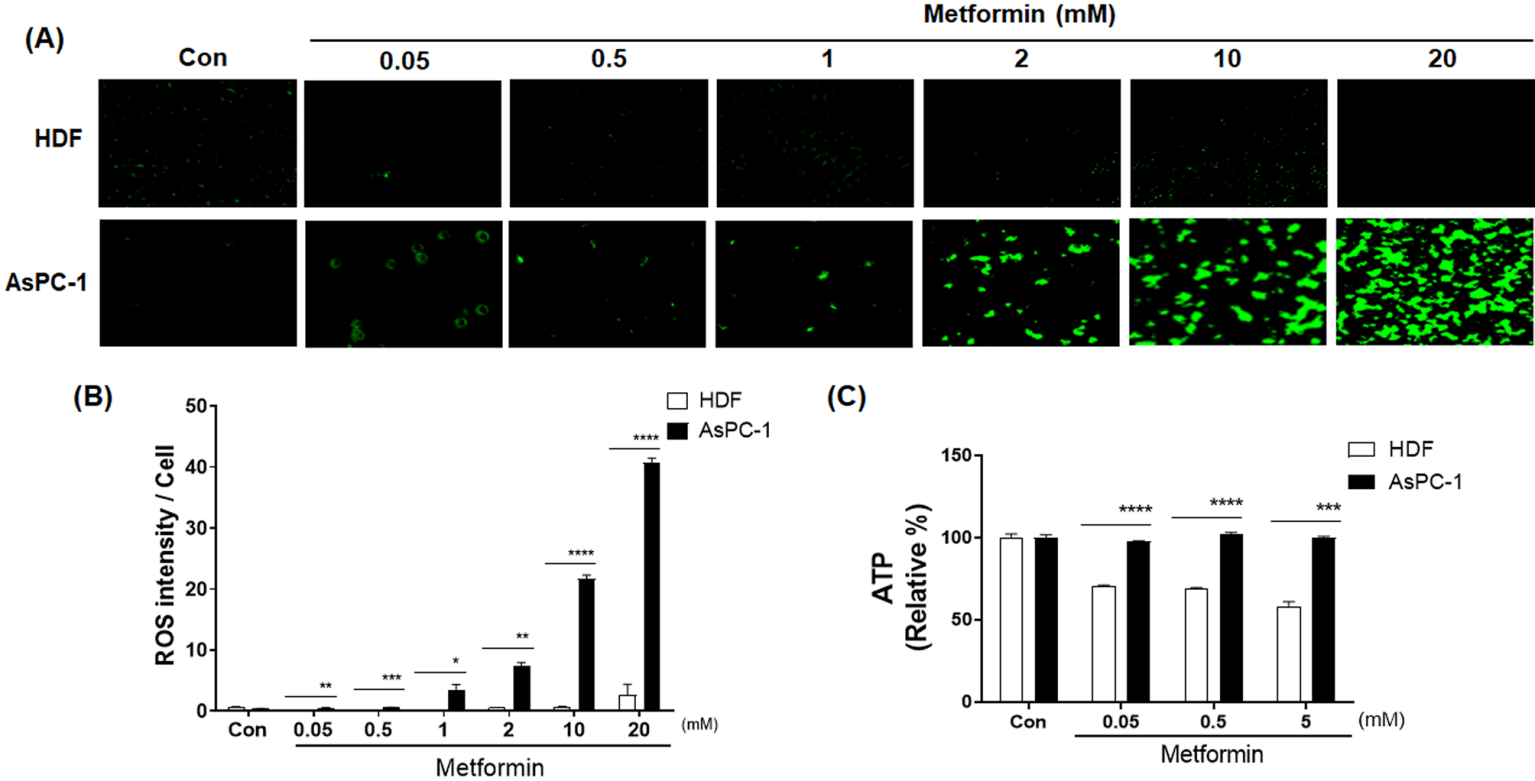
Effects of metformin on cellular ROS and ATP levels in human AsPC-1 and HDF cells. (**A**) Cellular ROS production was detected by CellROX Green staining in AsPC-1 and HDF cells after incubation with metformin (0, 0.05, 0.5, 1, 2, 10 and 20 mM) for 48 h. (**B**) Corresponding quantitative analysis of ROS levels. (**C**) Cellular ATP production was measured in ASPC-1 and HDF cells after incubation with metformin (0, 0.05, 0.5, and 5 mM) for 48 h. Three different measurements were performed for each sample. Statistical significance is indicated as *p < 0.05, **p < 0.01, ***p < 0.001, and ****p < 0.0001 compared with HDF group.

AsPC-1 (human pancreatic cancer) and HDF (human normal fibroblast) cells were treated with increasing concentration of metformin (0.05 to 20 mM) for 48 h. When AsPC-1 cells were treated with metformin, cellular ROS levels increased in a concentration-dependent manner (*p* < 0.01) (Fig. 1A,B). When HDF cells were treated with metformin, cellular ROS levels were not increased and remained at nearly an undetectable level (Fig. 1A,B). Cellular ATP levels were almost unchanged by metformin in ASPC-1 cells but deceased by up to 50% with metformin in HDF cells (*p* < 0.001) (Fig. 1C).

### Metformin causes nuclear localization of p-AMPK (Thr172) and p-FOXO3a (Ser413) by activating the AMPK/FOXO3a/MnSOD pathway in normal cells but not in cancer cells

**Figure 2:**
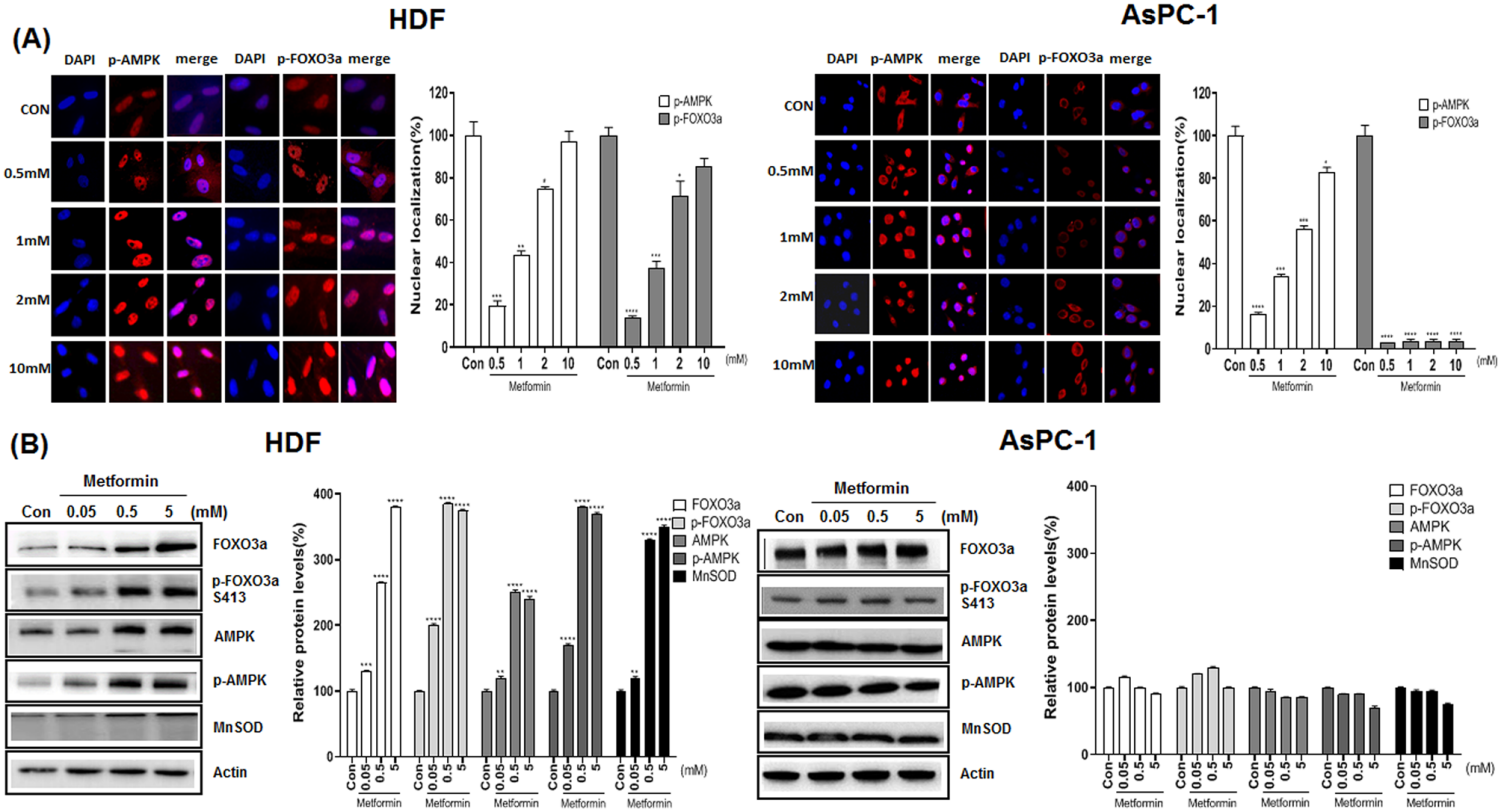
Nuclear localization and protein levels of p-AMPK (Thr172) and p-FOXO3a (Ser413) in metformin-treated AsPC-1 and HDF cells. AsPC-1 and HDF cells were treated with metformin (0.05, 0.5, 5 mM) for 24 h. (**A**) Immunostaining was performed in AsPC-1 and HDF cells using anti-p-AMPK (Thr172) and p-FOXO3a (Ser413) antibodies. Anti-mouse IgG, F(ab’)2 fragment (Alexa fluor 594 conjugate, red color) was used as the secondary antibody. DAPI (blue color) was used as the nucleus marker. (**B**) Levels of AMPK, p-AMPK (Thr172), FOXO3a, p-FOXO3a (Ser413) and MnSOD proteins were measured in metformin-treated AsPC-1 and HDF cells by western blot analysis. Three different measurements were performed for each sample. Statistical significance is indicated as *p < 0.05, **p < 0.01, ***p < 0.001, and ****p < 0.0001 compared with control group.

We compared AMPK activation by metformin in cancer and normal cells along with activation of FOXO3a, as the latter is downstream of AMPK. AsPC-1 and HDF cells were treated with metformin (0.5, 1, 2 and 10 mM) for 24 h and immunofluorescence analysis of the treated cells with anti p-AMPK (Thr172) and p-FOXO3a (Ser413) antibodies. As shown, p-AMPK (Thr172) and p-FOXO3a (Ser413) were localized in the nucleus for HDF cells but were more evenly distributed and mostly in the cytosol for AsPC-1 cells (Fig. 2A). Western blot analysis of cell lysates with anti AMPK, p-AMPK (Thr172), FOXO3a, p-FOXO3a (Ser413) and MnSOD antibodies indicated that metformin does not activate AMPK, FOXO3a and MnSOD in ASPC-1 cells; however, metformin (from 0.05 to 5 mM) up-regulated p-AMPK (Thr172), p-FOXO3a (Ser413) and MnSOD only in HDF cells (*p* < 0.01) (Fig. 2B).

### Transfection with wild type FOXO3a (wt-FOXO3a) and si-FOXO3a RNA suggests that FOXO3a activation plays a key role in determining cellular ROS levels

**Figure 3:**
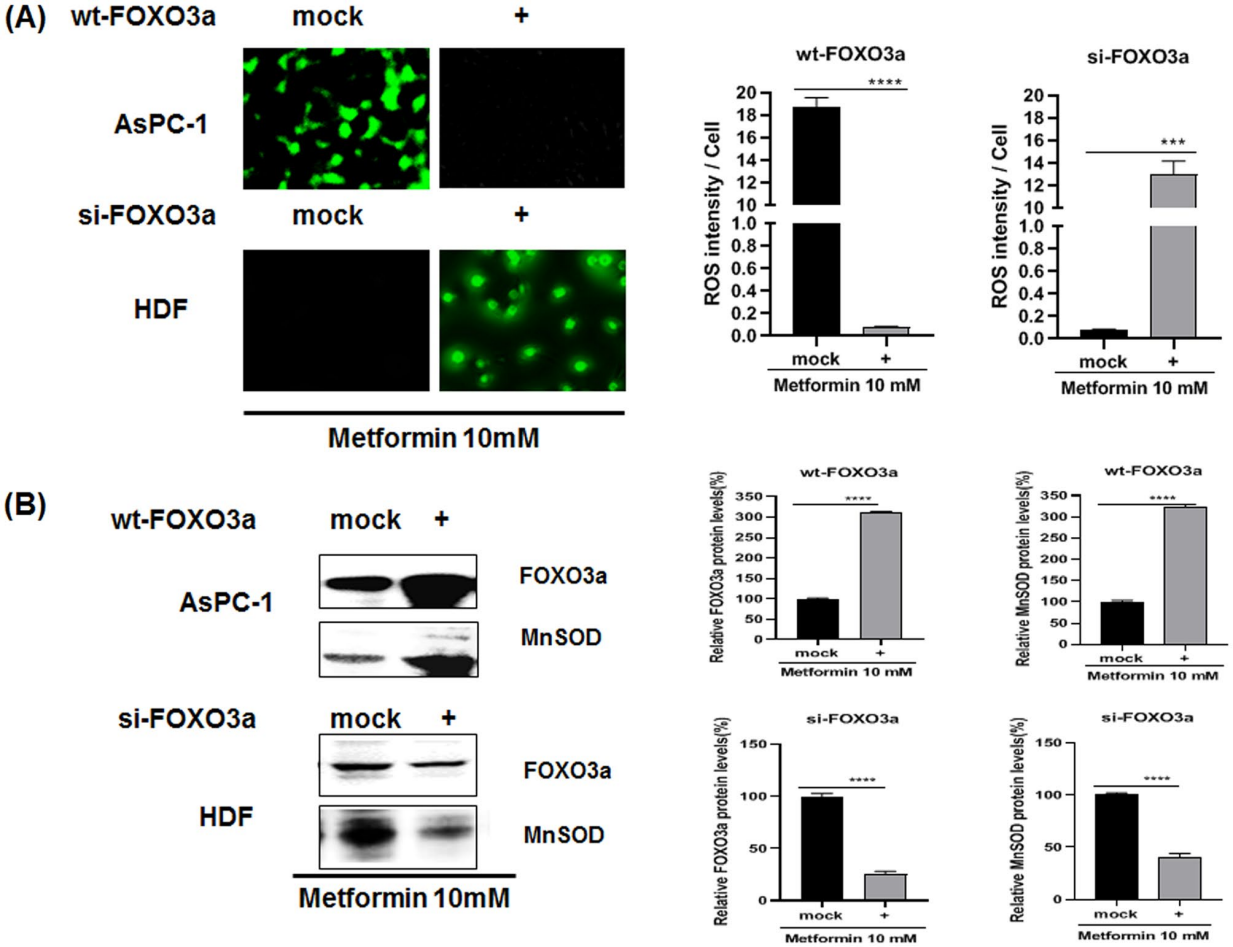
Effect of transfection with si-FOXO3a RNA or wt-FOXO3a plasmids on cellular ROS and protein levels in metformin-treated AsPC-1 and HDF cells. (**A**) Cellular ROS level of metformin-treated (for 24 h) AsPC-1 and HDF cells were measured by CellROX Green staining. AsPC-1 cells were transfected with si-FOXO3a RNA while HDF cells were transfected with wt-FOXO3a. The transfected cells were treated with metformin (10 mM) for 24 h; then cellular ROS levels were measured by CellROX Green staining. (**B**) Protein levels of FOXO3a and MnSOD were measured in the transfected and metformin-treated cells by western blot analysis using anti-FOXO3a and MnSOD antibodies. Three different measurements were performed for each sample. Statistical significance is indicated as *p < 0.05, **p < 0.01, ***p < 0.001, and ****p < 0.0001.

To examine whether FOXO3a activation contributes to cellular ROS levels, AsPC-1 cells were either mock transfected or transfected with wt-FOXO3a, both subsequently treated with 10 mM metformin. For HDF cells, they were mock transfected or transfected with si-FOXO3a RNA and then treated with 10 mM metformin as indicated (Fig. 3A). The intracellular ROS levels for this experiment were detected with CellROX staining of the cells and the cell lysates were also analyzed by western blotting (Fig. 3A,B). Cellular ROS levels were dramatically decreased in wt-FOXO3a-transfected AsPC-1 cells when compared with mock transfected cells (*p* < 0.0001) (Fig. 3A). Expression of FOXO3a and MnSOD proteins were also highly increased in wt-FOXO3a-transfected AsPC-1 cells compared with mock transfected cells (*p* < 0.0001) (Fig. 3B). On the other hand, cellular ROS levels were dramatically increased in si-FOXO3a RNA-transfected HDF cells (*p* < 0.001) when compared with mock transfected HDF cells and expression of FOXO3a and MnSOD were largely decreased in wt-FOXO3a-transfected HDF cells (*p* < 0.001) (Fig. 3A,B).

### Co-treatment with metformin and apigenin affects cell survival, apoptosis and cellular ROS levels, and inhibits mitochondrial potential

**Figure 4:**
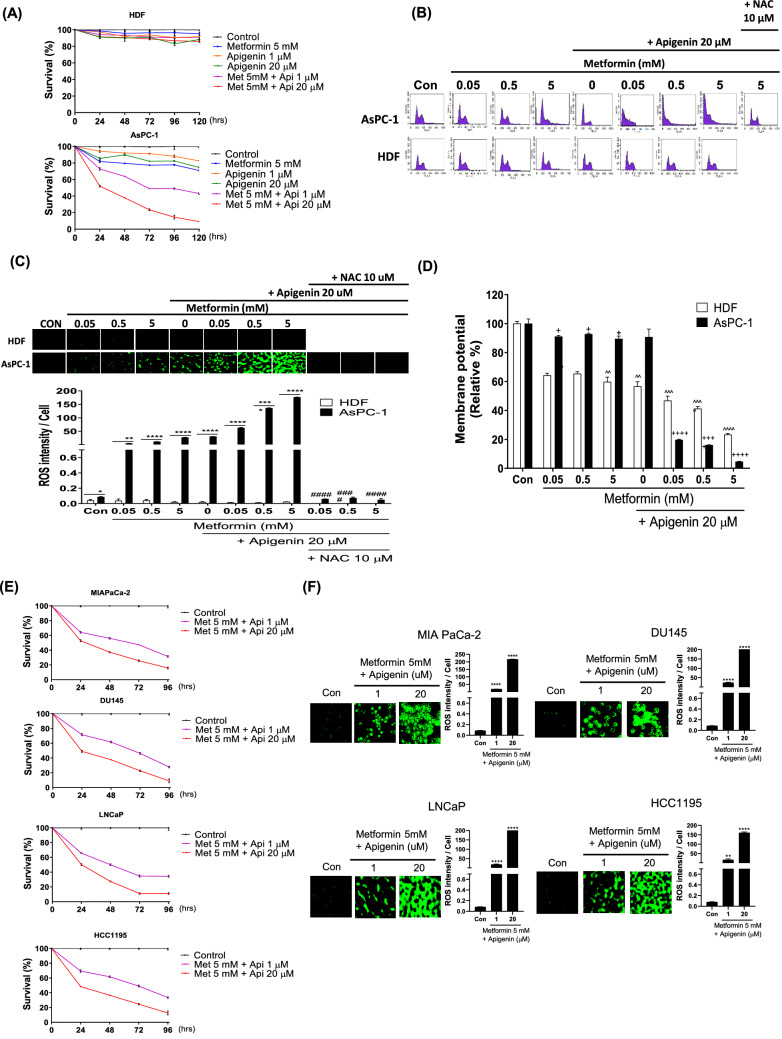
Effect of combination metformin/apigenin on cell growth, cell cycle, cellular ROS and mitochondrial membrane potential in AsPC-1 and HDF cells. (**A**) The effect of combination of metformin/apigenin on cell proliferation/viability in AsPC-1 and HDF cells. The cells were treated with metformin (5 mM) alone, apigenin (1 or 20 µM) alone or metformin/apigenin combination for 24 to 120 h and the cell proliferation/viability was assessed by MTT assay. (**B**) Cell cycle was analyzed with PI staining of the cells (AsPC-1 and HDF) treated with metformin (0.05, 0.5 or 5 mM) alone, apigenin (20 µM) alone or metformin/apigenin combination. (**C**) Cellular ROS levels of metformin-treated (for 24 h) in AsPC-1 and HDF cells were measured by CellROX Green staining when cells (AsPC-1 and HDF) were treated with metformin (0.05, 0.5 or 5 mM), apigenin (20 µM) or combination of metformin and apigenin for 24 h. (**D**) Mitochondrial membrane potentials were measured when cells (AsPC-1 and HDF) were treated with metformin (0.5 or 5 mM), apigenin (20 µM) or combination of metformin/apigenin for 24 h. Effect of NAC (N-acetyl cysteine, 10 µM) pretreatment on ROS production (**A**) and cell death (**B**) induced by combination of metformin/apigenin was examined. The effects of combination of metformin (5 mM)/apigenin (1 and 20 μM) on cell proliferation/viability (**E**) and cellular ROS level (**F**) in MIAPaCa-2, DU145, LNCaP and HCC1195 cells. Three different measurements were performed for each sample. Statistical significance is indicated as *p < 0.05, **p < 0.01, and ****p < 0.0001 compared with control group. ^####^*p* < 0.0001 compared with metformin (0.05, 0.5 or 5 mM)/apigenin (20 µM) treated group. ^+^*p* < 0.05, ^+++^*p* < 0.01, and ^++++^*p* < 0.0001 compared with ASPC-1 control group. ^^*p* < 0.01, ^^^*p* < 0.001 and ^^^^*p* < 0.0001 compared with HDF control group.

To examine the anticancer activity of co-treatment with metformin and apigenin, AsPC-1 cells and HDF cells were treated with either metformin (5 mM), apigenin (1 or 20 µM), or both metformin and apigenin up to 120 h. Cell growth/viability changes was examined by the MTT assay. The results showed that metformin or apigenin alone causes little change in cell growth/viability for AsPC-1 cells; however, co-treatment of the cells with metformin and apigenin cells led to a significant inhibition of cell growth/viability (Fig. 4A). In contrast, for HDF cells co-treatment with metformin and apigenin did not significantly affect cell growth/viability (Fig. 4A). Cell cycle analysis via flowcytometry and cellular ROS level changes via CellROX were also performed in response to varying levels of metformin and in the presence or absence of apigenin (20 µM) in cancer cells treated for 24 h. Cell cycle analysis showed that co-treatment with metformin and apigenin induces cell death in majority of AsPC-1 cells (Fig. 4B). As shown in Fig. 4C, co-treatment with metformin (0.05, 0.5 or 5 mM) and apigenin (20 µM) dramatically increased cellular ROS levels in AsPC-1 cells (*p* < 0.0001). However, the same co-treatment did not affect cellular ROS levels or extent of cell death in HDF cells (Fig. 4B,C). In addition, the effect of N-acetyl cysteine (NAC), an ROS scavenger, was gauged when co-treating the cancer cells in the presence of metformin and apigenin. From cell cycle analysis, NAC blocked the metformin/apigenin co-treatment-induced cell death in AsPC-1 cells (Fig. 4B) and NAC also blocked ROS increases seen with co-treatment with metformin and apigenin in the same cells (*p* < 0.0001) (Fig. 4C). Combination of metformin (0.05, 0.5 or 5 mM) and apigenin (20 µM) also synergistically inhibited mitochondrial membrane potential in AsPC-1 cells (*p* < 0.0001) (Fig. 4D), whereas the same treatment had a lesser effect in HDF cells (*p* < 0.001) (Fig. 4D). In addition to AsPC-1 cells, co-treatment with metformin and apigenin decreased cell viability and increased ROS levels (*p* < 0.01) in a synergistic manner in other cancer cells such as MIAPaCa-2, DU145, LNCaP and HCC1195 cells (Fig. 4E,F).

### Combination of metformin and apigenin leads to DNA damage-induced apoptosis, autophagy and necroptosis in AsPC-1 cells but not in HDF cells

**Figure 5:**
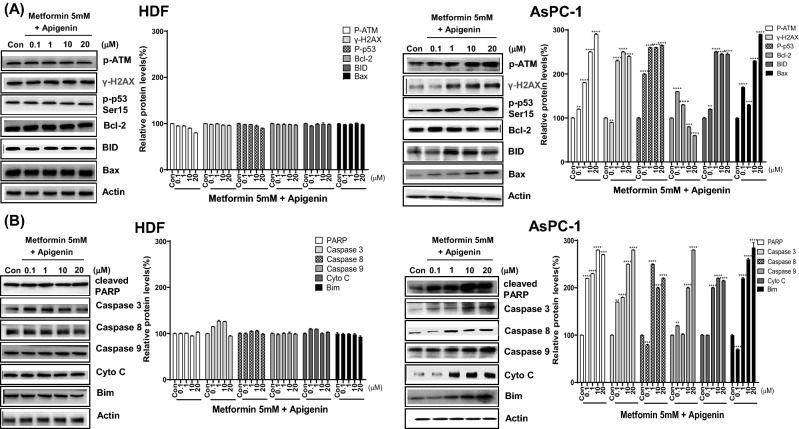
Levels of DNA damage- and apoptosis-related proteins in metformin and apigenin-cotreated AsPC-1 and HDF cells. AsPC-1 and HDF cells were treated with metformin (5 mM) and apigenin (0.1, 1, 10 or 20 μM) for 48 h. Cell extracts were prepared and subjected to western blot analysis. DNA damage-related proteins were analyzed with anti-p-ATM, H2AX, and p-p53 (Ser15) antibodies. Apoptosis-related proteins were analyzed with anti-BID, Bax, cleaved-PARP, cleaved-caspase 3, 8, 9, cytochrome C, and Bim antibodies. Three different measurements were performed for each sample. Statistical significance is indicated as *p < 0.05, **p < 0.01, ***p < 0.001, and ****p < 0.0001 compared with control group.

**Figure 6:**
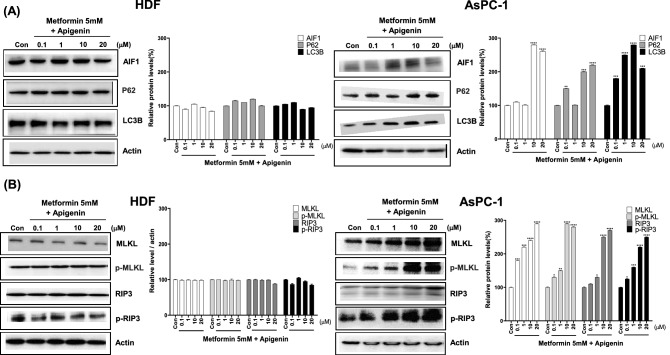
Levels of autophagy- and necroptosis-related proteins in metformin/apigenin-combination treated AsPC-1 and HDF cells. AsPC-1 and HDF cells were treated with metformin (5 mM) and apigenin (0.1, 1, 10 or 20 μM) for 48 h. Cell extracts were prepared and subjected to western blot analysis. Autophagy-related proteins were analyzed with anti-AIF, P62 and LC3B antibodies. Necroptosis-related proteins were analyzed with anti-MLKL, p-MLKL, RIP3 and p-RIP3 antibodies. Three different measurements were performed for each sample. Statistical significance is indicated as *p < 0.05, **p < 0.01, ***p < 0.001, and ****p < 0.0001 compared with control group.

To examine the mechanism involved in combination of metformin and apigenin-induced cell death, expression of DNA damage-related proteins was measured by western blot analysis in AsPC-1 and HDF cells. Levels of p-ATM, γ-H2AX, and DNA damage markers were increased by combination of metformin and apigenin in AsPC-1 cells (*p* < 0.05), indicating that amplified ROS induced severe DNA damage (Fig. 5A). DNA damage appeared to induce apoptosis as the levels of p-p53, Bim, Bid, Bax, cleaved PARP, caspase 3, caspase 8, and caspase 9 were also significantly increased by combination of metformin and apigenin in AsPC-1 cells (*p* < 0.05), and not HDF cells (Fig. 5B). Cytochrome C was also released from mitochondria in AsPC-1 cells, along with Bcl-2, an anti-apoptotic marker, becoming decreased in AsPC-1 cells (*p* < 0.01) (Fig. 5B). Interestingly, autophagy-related proteins (AIF, P62 and LC3B) and necroptosis-related proteins (MLKL, p-MLKL, RIP3 and p-RIP3) were also increased by combination of metformin and apigenin (*p* < 0.05), suggesting that autophagy and necroptosis were also involved (Fig. 6A,B). In comparison, DNA damage markers, apoptosis-, autophagy-, and necroptosis-related proteins were not altered by combination of metformin and apigenin in HDF cells (Fig. 6A,B).

### Combination of metformin and apigenin effectively reduces tumor growth in an in vivo model

**Figure 7:**
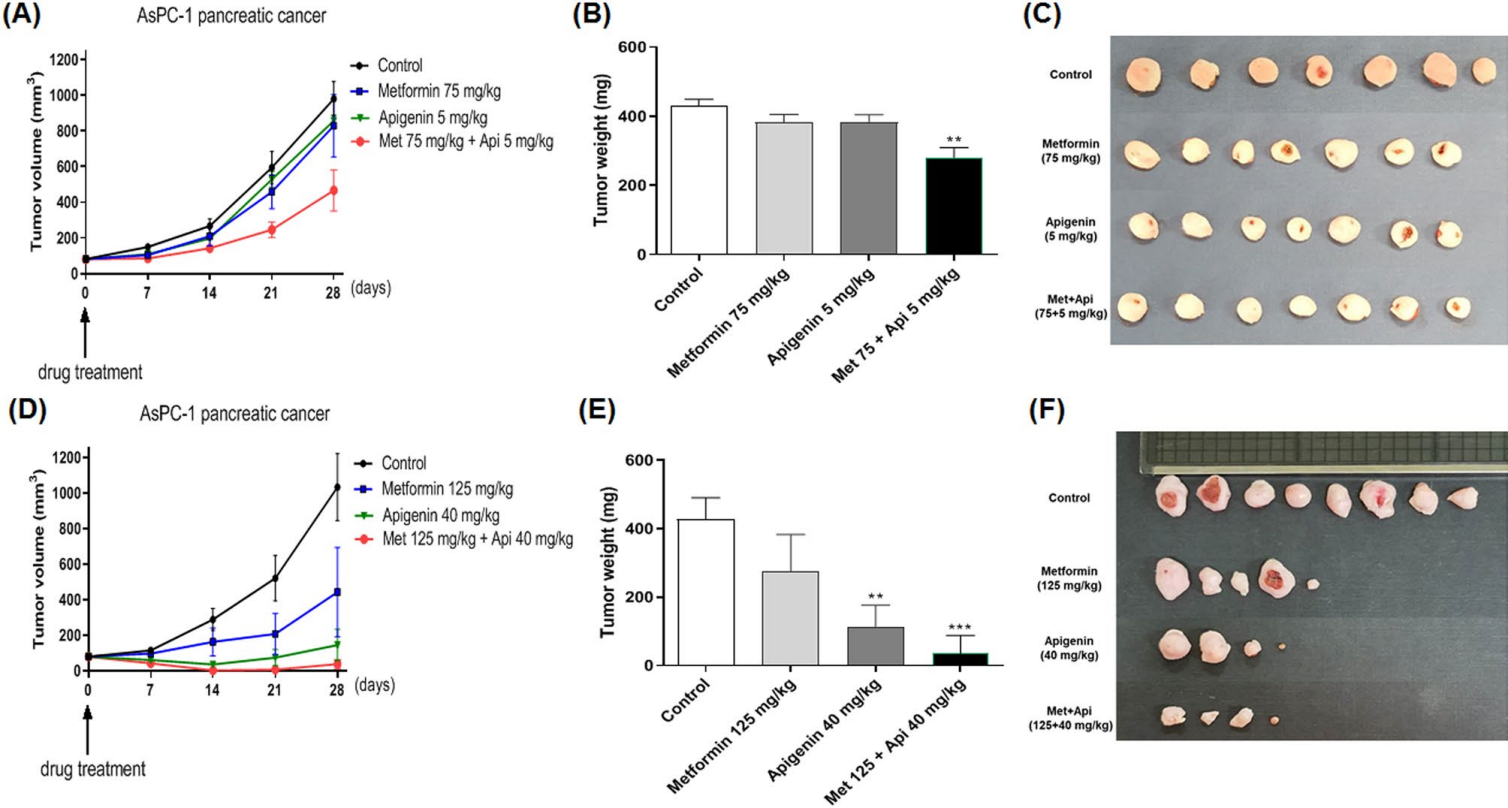
Effect of metformin and apigenin on tumor growth in xenograft model of nude mice. AsPC-1 (1 × 10^7^ cells in 100 μl) cells were injected subcutaneously into the dorsal flank of athymic nude mice. When tumors reached a size of approximately 80 mm^3^, mice were administered orally with metformin (75 mg/kg), apigenin (5 mg/kg), or combination of two drugs in a group. In another group, mice were administered orally with metformin (125 mg/kg), apigenin (40 mg/kg) or the combination of two drugs. The drugs were administered twice a day for a total of 28 days. (**A**) and (**D**): Tumor volume was measured for up to four weeks. (**B**) and (**E**): Weights of excised tumors were measured at the end of the study. (**C**) and (**F**): Photographs of excised tumors in each group are shown. Statistical significance is indicated as **p < 0.01, and ***p < 0.001 compared with control group.

To test the effect of combination of metformin and apigenin on tumor growth, AsPC-1 (1 × 10^7^ cells) cells were injected into athymic nude mice to generate a xenograft cancer model. When the xenografts had reached about 80 mm^3^ in size, the mice were randomized into treatment groups of control (vehicle treated), metformin (75 or 125 mg/kg), apigenin (5 or 40 mg/kg), or metformin/apigenin combination. The control/drugs were given orally and twice daily as described in the “Materials and methods” section. As depicted in Fig. 7A,D, the treatments were continued for a period of 4 weeks, with the tumor sizes monitored as the control group reached an average of 1000 mm^3^ in size (starting from 80 mm^3^ in size). As seen in Fig. 7A, administration of metformin (75 mg/kg) or apigenin (5 mg/kg) alone caused a little change of tumor size, but a combination of two drugs decreased tumor size and weight in a synergistoical manner (Fig. 7B,C). Administration with higher dose of metformin (125 mg/kg) or apigenin (40 mg/kg) caused a reduction of tumor size compared to the control group (Fig. 7D). However, oral administration of combination of metformin and apigenin decreased tumor weight profoundly (*p* < 0.01) (Fig. 7E,F).

## Discussion

**Figure 8:**
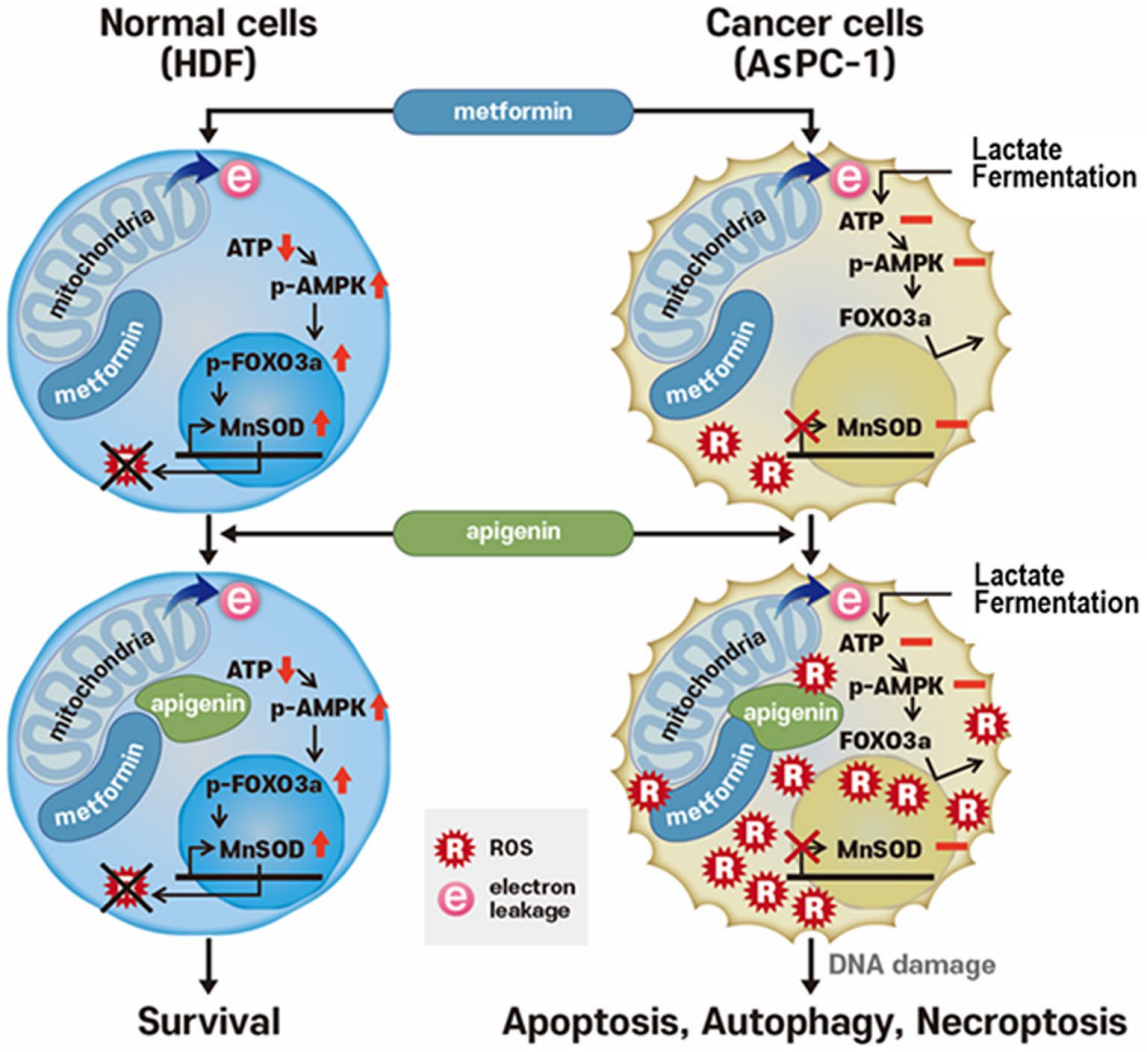
Hypothetical diagram depicting mechanism of differential cell death induced by combination of metformin and apigenin between HDF and AsPC-1 cells.

In the present study, metformin increases ROS production in AsPC-1 cancer cells, but not in the HDF normal cells. In addition, for AsPC-1 cells, ATP levels were not changed, whereas ATP levels were decreased in HDF cells. Metformin-induced ROS production in normal cells appears to be primarily associated with signal molecules such as AMPK, FOXO3a, and MnSOD. Our study clearly demonstrated that in normal cells metformin increases AMPK and p-AMPK levels and in turn, elevations in FOXO3a and p-FOXO3a, finally leading to increases in MnSOD levels. MnSOD causes a decrease in existing levels of ROS. For normal cells, metformin leads to AMPK being activated from a decrease in ATP production in mitochondria. In contrast to the normal cells, metformin did not affect AMPK, FOXO3a, and MnSOD levels in AsPC-1 cancer cells. Thus, increased ROS levels in cancer cells appear to be due to the lack of MnSOD action. AMPK-induced activation of FOXO3a is a key step in allowing a differential response between normal and cancer cells via metformin. AMPK-mediated phosphorylation of FOXO3a S413 activates FOXO3a. When FOXO3a levels were reduced via si-FOXO3a transfection, ROS increases were also seen in normal cells. We also found that when FOXO3a levels are raised by transfection via wt-FOXO3a, ROS levels becomes undetectable in cancer cells. This result clearly suggests that FOXO3a is a key molecule in bringing about this anticancer activity seen with metformin. In normal cells, many drugs are known to bind to mitochondria, generate ROS and decrease production of ATP upon producing electron leakage from the mitochondrial ETC. Some of these agents like metformin and apigenin result in a mild leakage in the ETC^30,31^ and do not affect cellular integrity and cell survival. However, certain other chemicals such as KCN and arsenic cause severe mitochondrial damage and induce cell death^32,33^. If agents that are not cytotoxic to normal cells and only mildly affect mitochondrial membrane potential are combined, they may still be excellent for amplifying ROS levels and inducing apoptosis in target cancer cells. One such agent is apigenin that is known to bind mitochondria and only mildly decrease the membrane potential in the treated cells^30,31^. Treatment with metformin or apigenin alone in normal fibroblasts did not affect cell viability. For the same cells, the combination of metformin and apigenin decreased mitochondrial membrane potential greatly but it did not affect cellular integrity and cell viability. In the present study, 5 mM of metformin itself inhibited MMP just partially in HDF cells. This observation is in part correlated with finding that IC50 value of metformin for MMP inhibition reported previously is 19 mM^34^. However, what we observed in the present is the finding that 5 mM of metformin could strongly inhibit MMP when metformin (0.05, 0.5 or 5 mM) was combined with apigenin (20 µM). This finding suggests that metformin interacts with apigenin for MMP inhibition in a synergistic manner. Based on our results, the overall hypothetical diagram depicting differential cell death by combination of metformin and apigenin between normal and cancer cells is described (Fig. 8). The exact reason for the synergistic increase on ROS production with the combination of metformin and apigenin in many cancer cells is not currently clear. However, it is speculated that metformin and apigenin may act via different mechanisms on the electron transport system in mitochondria and these additively produce ROS in mitochondria in cancer cells. Our findings suggest that the decrease of membrane potential by metformin and apigenin appeared to be synergistic when compared to treatment by each drug alone. Our finding is in line with a previous study in that the decrease of membrane potential by treatment metformin is well related with the increase of ROS production^35^.

We also found the combination of metformin and apigenin causing a reduction in cell growth in AsPC-1, MIAPaCa-2, LNCaP, DU145 and HCC1195 cells in a synergistic manner as revealed by the MTT assay and an induction of apoptosis in AsPC-1 cells as revealed by cell cycle analysis. This synergistic interaction was not observed in HDF cells, suggesting that cell growth inhibition and apoptosis induction by combination of metformin and apigenin are cancer cell specific. The nullifying effects of NAC against metformin/apigenin-induced ROS increase and apoptosis suggest that overproduction of ROS level appears to be primarily responsible for the cell death. Excessive amounts of ROS can cause oxidative damage to lipids, proteins, and DNA^5^. It is documented that if increases in ROS reaches a certain threshold that is detrimental to the cell with ROS exerting a cytotoxic effect. For cancer cells, this may lead to cell death and thus limit cancer progression^36–38^. In our observations, a combination of metformin and apigenin generated much higher levels of ROS over that perceived threshold, bringing about irreversible DNA damage.

The current study indicate that cell death induced by the combination of metformin and apigenin is mediated through apoptosis, autophagy, and necroptosis as there were increases in the levels of the proteins for each of these processes. For normal cells, these changes were not seen by the combination of metformin and apigenin, suggesting that the anticancer activity induced by these two agents might be achieved by selective activation of apoptosis, autophagy, and necroptosis pathways only for cancer cells.

From our in vivo experiment, we found that the individual oral administration with a lower dose of metformin (75 mg/kg) or apigenin (5 mg/kg) alone for 4 weeks did not affect much on tumor volume and weight. However, a combination of metformin and apigenin for 4 weeks caused a synergistic reduction in tumor volume. In addition to this finding, we found that individual oral administration of metformin (125 mg/kg) or apigenin (40 mg/kg) alone at higher doses, to some extent, reduced tumor growth in the ASPC-1 xenograft. However, oral administration of a combination of both metformin and apigenin almost completely inhibited tumor growth, suggesting that a combination of the two drugs exerts a more profound anticancer effect in vivo.

Several studies have reported that apigenin exerts an inhibition on tumor growth in several cancer xenograft models^39–41^. In addition, administration of metformin has also been documented to have a decrease in tumor growth in several animal cancer models^42,43^. However, as metformin and apigenin individually do not exert sufficiently robust antitumor activity, our findings suggest that combining metformin with apigenin may be useful to further test in preclinical models of pancreatic and other cancer types and potentially for a new class of normal-cell sparing anticancer drugs.

## Materials and methods

### Reagents

MTT reagents and propidium iodide (PI) were obtained from Sigma-Aldrich (St. Louis, MO). The CellROX Green reagent, DMEM, FBS, trypsin, penicillin–streptomycin and Hanks Balanced Salt Solution were purchased from Thermo Fisher Scientific (Waltham, MA); Lipofectamine was obtained from Invitrogen (Carlsbad, CA). The Lowery assay reagent was from BioRad (Hercules, CA). The immunoblot PVDF (polyvinylidene difluoride) membrane and Immobilon reagent were both purchased from Millipore (St. Louis, MO). The Mitochondrial Membrane Potential kit and the ATP Assay kit were purchased from Cayman (Ann Arbor, MI). The Lactate Assay and Mitochondrial Isolation kits along with the PI/RNase solution were purchased from Abcam (Cambridge, MA). Anti-AMPK, p-AMPK (Thr172), FOXO3a, p-FOXO3a (Ser413) and MnSOD p-ATM, γ-H2AX, p-p53, Bim, Bid, Bax, cleaved-PARP, cleaved-caspases 3, 8, and 9, cytochrome C and Bcl-2 antibodies and the secondary anti-mouse IgG-horseradish peroxidase antibody were obtained from Cell Signaling (Danvers, MA).

### Cell culture

The human cancer cell lines such as AsPC-1 (human pancreatic cancer), MIAPaCa-2 (human pancreatic cancer), LNCaP (human prostate cancer), DU145 (human prostate cancer) and HCC1195 (human lung cancer) were purchased from the Korean Cell Line Bank (Seoul, Korea) and the HDF (human primary dermal fibroblast) was obtained from the Dermatology Laboratory of Seoul National University Medical School (Seoul, Korea). The cultured cells were grown in the media of DMEM with 10% FBS-penicillin–streptomycin in a humidified 5% CO_2_ incubator at 37 °C.

### MTT assay

The 3-(4,5-dimethylthiazol-2-yl)-2,5-diphenyltetrazolium bromide (MTT) colorimetric assay protocol was performed as described by Mosmann^44^. For the assay, the cells were seated in 96-well culture plates (2 × 10^3^ cells/well) for 24 h. The cells were then treated with the indicated drugs or control (DMSO carrier) up to 120 h. The culture media in each well was replaced with 10 µl of stock MTT solution in 100 µl of DMEM and the plates were incubated for 4 h. MTT solution was replaced with 100 µl of DMSO. The plates were incubated for 1 h. The absorbance yields of control and drug-treated wells were measured at 570 nm using an automated microplate reader (Thermo Fisher Scientific, Waltham, MA). These values were then used to calculate the viability/proliferation changes relative to control wells.

### Cell cycle analysis

Drug-treated/control cells were grown for 48 h and then harvested by trypsinization. The cells were then washed with 1 × PBS and fixed in 70% ethanol at − 20 °C overnight. After the fixation, the cells were washed twice with 1 × PBS and then collected by centrifugation. The cells were resuspended in PI/RNase solution (Abcam) and incubated for 1 h. The cell cycle was measured using the FACS Vantage SE cell sorter (Becton Dickinson, Franklin Lakes, NJ). Cell cycle analysis was performed using ModFit LT software (Verity Software, Topsham, ME).

### Immunofluorescence

The cellular localization of p-FOXO3a (Thr172) or p-AMPK (Ser413) proteins in AsPC-1 and HDF cells was determined by immunofluorescence antibody staining. First, the cells were treated with 0.5, 1, 10 and 20 mM of metformin, grown on glass slides for 24 h and were then washed with 1 × PBS. The cells were fixed in 4% formaldehyde at room temperature for 15 min; they were then washed with 1xPBS and incubated in blocking buffer (1% BSA in PBS) for 1 h. The slides were again washed with 1 × PBS and then diluted anti-FOXO3a, p-FOXO3a (Ser413), AMPK, p-AMPK (Thr172) antibodies were added to the cells on the slides. The slides were incubated in dark at 4 °C overnight. After washing the slides twice with 1 × PBS, the diluted fluorophore-conjugated secondary antibody was added and incubated at room temperature in dark for 1 h. After washing the slides twice with 1 × PBS, the slides were incubated with the DAPI solution (0.1 µg/ml double distilled water) for 2 min and washed with 1 × PBS for three times. One drop of mounting reagent was added to the slides, and were covered with a cover glass and incubated at room temperature overnight. The photographs were taken using a confocal microscope (Carl Zeiss LSM710).

### ROS measurement

To determine the cellular ROS levels in drug-treated AsPC-1 and HDF cells, the cells were grown for 24 h and then washed with 1 × PBS. The cells were incubated with the fluorogenic probe, CellROX Green, for 2 h and were then fixed with 4% paraformaldehyde. The photographs were taken using a fluorescence microscope (absorption 485 nm, emission 520 nm) (Zeiss Axiovert 200, Zeiss, Oberkochen, Germany). ROS intensity of each picture was quantified using Photoshop CS4 (Adobe Systems, San Jose, CA) software after subtraction of background fluorescence measured in the nucleus. Cellular ROS level was calculated through dividing ROS intensity by cell numbers in a picture and plotted in bar.

### Western blots analysis

The cells were first harvested by trypsinization and then washed twice with cold 1 × PBS. The cells were resuspended in the extraction buffer (150 mM NaCl/50 mM EDTA, pH 8.0/1% Non-diet p-40) containing the mixture of protease inhibitors (1 mM phenylmethyl methyl sulfonyl fluoride, 5 µg/ml aprotinin, 5 µg/ml leupeptin). After centrifugation at 14,000 rpm, 4 °C for 30 min, the supernatant was collected and used for western blot analysis, with the remaining lysate stored at − 70 °C. The protein concentration of each lysates was determined using the Lowry protein assay reagent (Thermo Fisher Scientific, Waltham, MA). The protein bands of the cell lysates were resolved in 12% SDS-PAGE gels and they were then electrically transferred onto a PVDF membrane for western probing. The membrane was blocked in 5% nonfat powder milk in TBST (50 mM Tris, pH 7.5, 150 mM NaCl, 0.1% Tween 20) for 2 h. The membrane was incubated overnight with the probing antibody diluted in TBST, washed and incubated with the diluted-anti-mouse IgG-horseradish peroxidase for 2 h. The resulting protein bands were visualized with the Immobilon reagent according to the manufacture’s protocol (Merck Millipore, Burlington, MA). The protein band intensity for each membrane probing was captured and measured using an image analyzer (Fusion FX7, Vilber Korea, Seoul, Korea). The final figures were prepared from original protein bands using Adobe Photoshop CS4 program.

### In vivo test of anticancer activity

All procedures were conducted in accordance with the ‘Guide for Care and Use of Laboratory Animals’ published by the National Institute of Health. This work was carried out in compliance with the ARRIVE guidelines^45^. Four-week-old female athymic nude mice (Koatech, Seoul, Korea) were received and allowed to acclimatize for a week. AsPC-1 cells (1 × 10^7^ cells/100 µl) were then injected subcutaneously at the flank region of mice. Drug treatment was initiated at 7-days post injection of the cancer cells with the tumors being palpable to a mean volume of 80 mm^3^. The animals were randomly allocated to four groups (8 mice per group); these included the control group (vehicle treated); metformin group; apigenin group; and the combination group. Control (vehicle treated), metformin (75 or 125 mg/kg), apigenin (5 or 40 mg/kg) or the combination of two drugs were dissolved in 0.5% carboxymethyl cellulose (CMC); Drugs were twice daily, orally administrated. Tumor volume and body weight were measured once a week. The tumor volume assessment was with a Vernier caliper measurement along two perpendicular axes of the tumor lump, using the formula of total volume being equal to (length × width^2^)/2. After 28 days of treatment, the mice were sacrificed by cervical vertebral dislocation and the tumors were extracted immediately. Tumor weights at the sacrifice time were then measured. All animal procedures and experimental protocols were approved by Laboratory Animal Committee of Hallym University (Hallym 2020-23).

### Statistical analysis

Statistical analysis was carried out by student t test using the GraphPad Prism Version 4.0 software for Windows (GraphPad Software, San Diego, CA). *P*-values of less than 0.05 were considered to indicate statistical significance. All values were expressed as mean ± S.E.M.

## Supplementary Information

Supplementary Information.

## Abbreviations

ROS: Reactive oxygen species
H_2_O_2_: Hydrogen peroxide
ETC: Electron transport chain
AMPK: AMP-activated protein kinase
mTOR: Mammalian target of the rapamycin
FOXO3a: Forkhead box O3a
PVDF: Polyvinylidene difluoride
CMC: Carboxymethyl cellulose
