# Supplementary material for: Metformin-induced ROS upregulation as amplified by apigenin causes profound anticancer activity while sparing normal cells
Source: Sci Rep. 2021 Jul 7;11:14002. doi: 10.1038/s41598-021-93270-0 (PMC8263563; doi:10.1038/s41598-021-93270-0)
Supplement: Supplementary file 1 — Supplementary Information. [file 41598_2021_93270_MOESM1_ESM.pdf]

Supplementary Information File

Metformin-induced ROS upregulation as amplified by apigenin causes profound anticancer activity while sparing normal cells

Madhuri Shende Warkad, Chea-Ha Kim, Beom-Goo Kang, Soo-Hyun Park, Jun-Sub Jung, Jing-Hui Feng, Gozde Inci, Sung-Chan Kim, Hong-Won Suh, Soon Sung Lim & Jae-Yong Lee

Fig.1 A ROS analysis (AsPC-1)

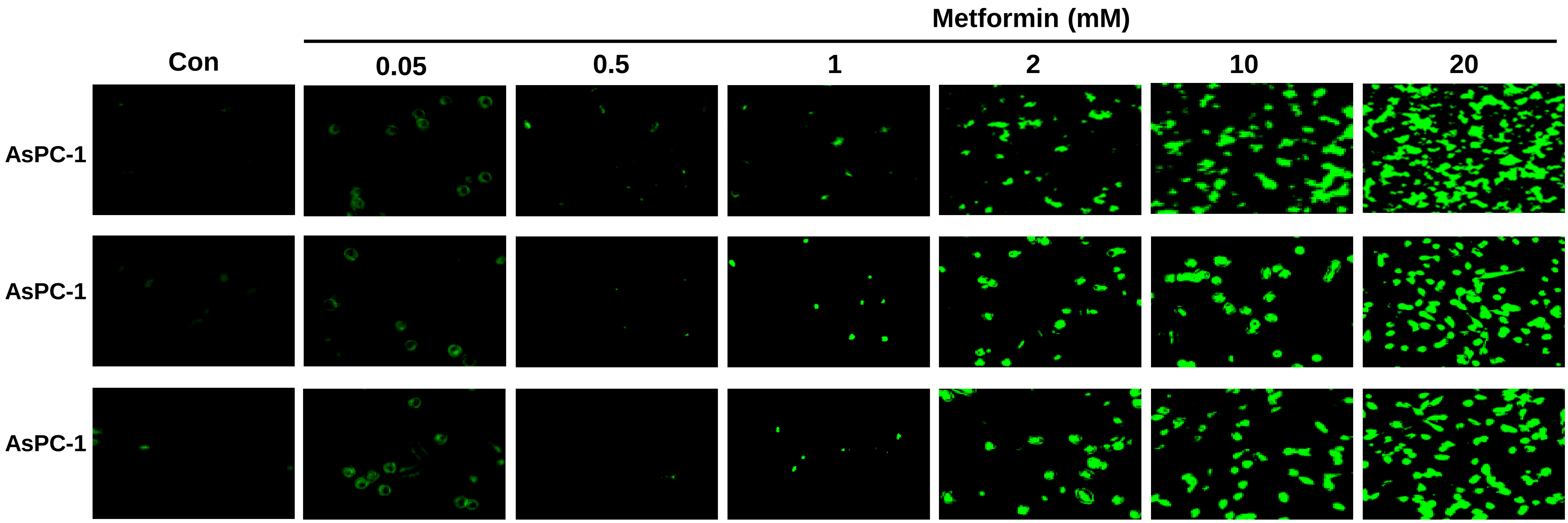

Fig.1 A ROS analysis (HDF)

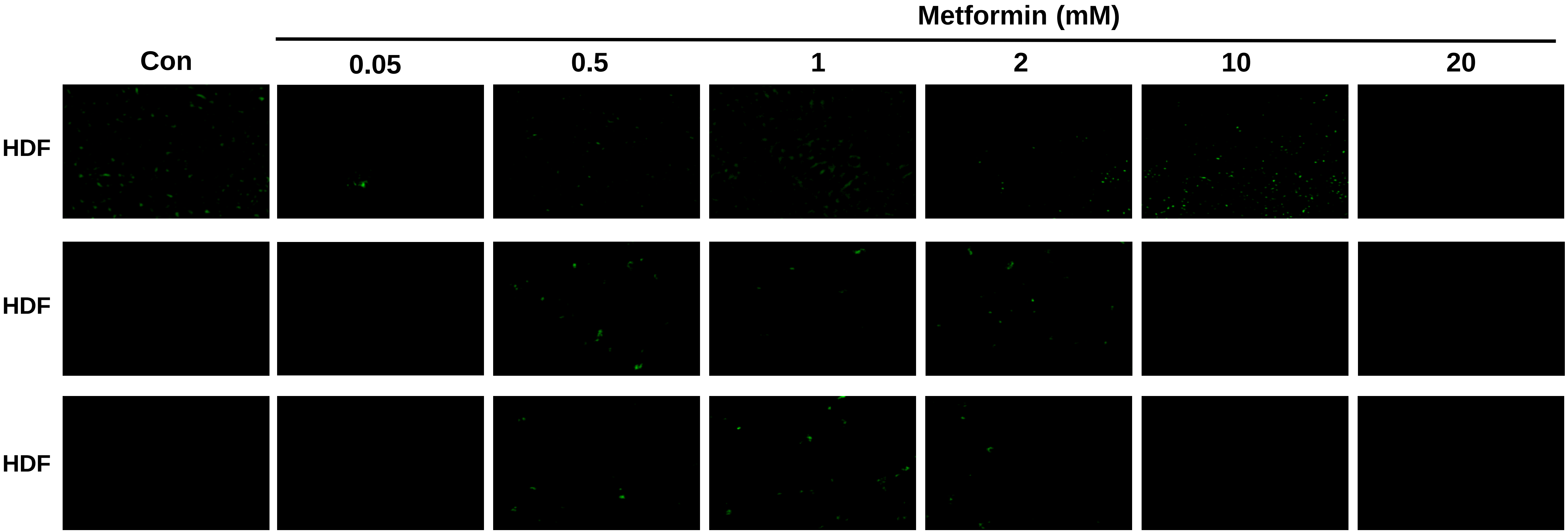

Fig.1 B&C ROS intensity and ATP (HDF & AsPC-1)

(B)

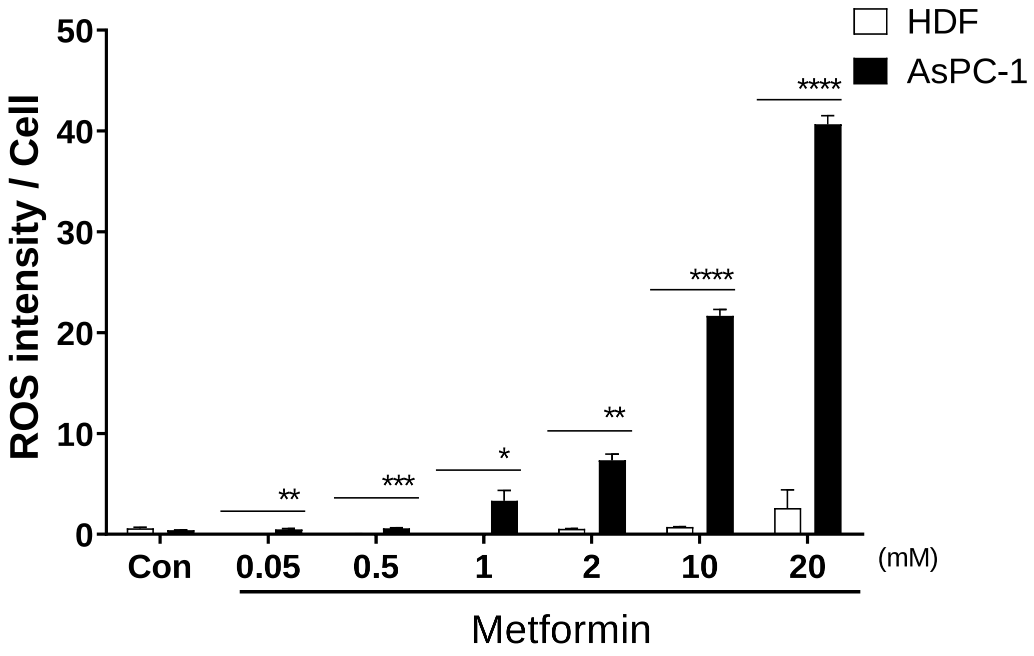

(C)

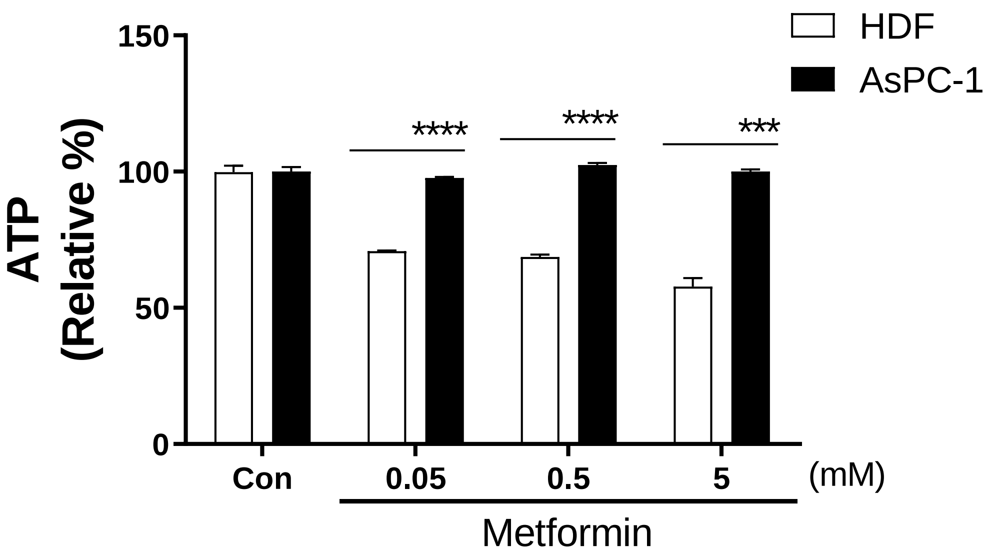

Figure 1. C) Cellular ATP production AsPC-1 and HDF cells

AsPC-1 ATP			
Control	0.5mM Me	5mM Me	20μM Apigenin
0.41	0.43	0.42	0.24
0.43	0.43	0.42	0.25
0.43	0.44	0.43	0.23
HDF			
con	0.5mM Me	5mM Me	20uM Apigenin
0.253	0.165	0.141	0.115
0.247	0.168	0.155	0.128
0.234	0.172	0.129	0.142

Fig. 2A. Nuclear Transport: Immunofluorescence HDF-1

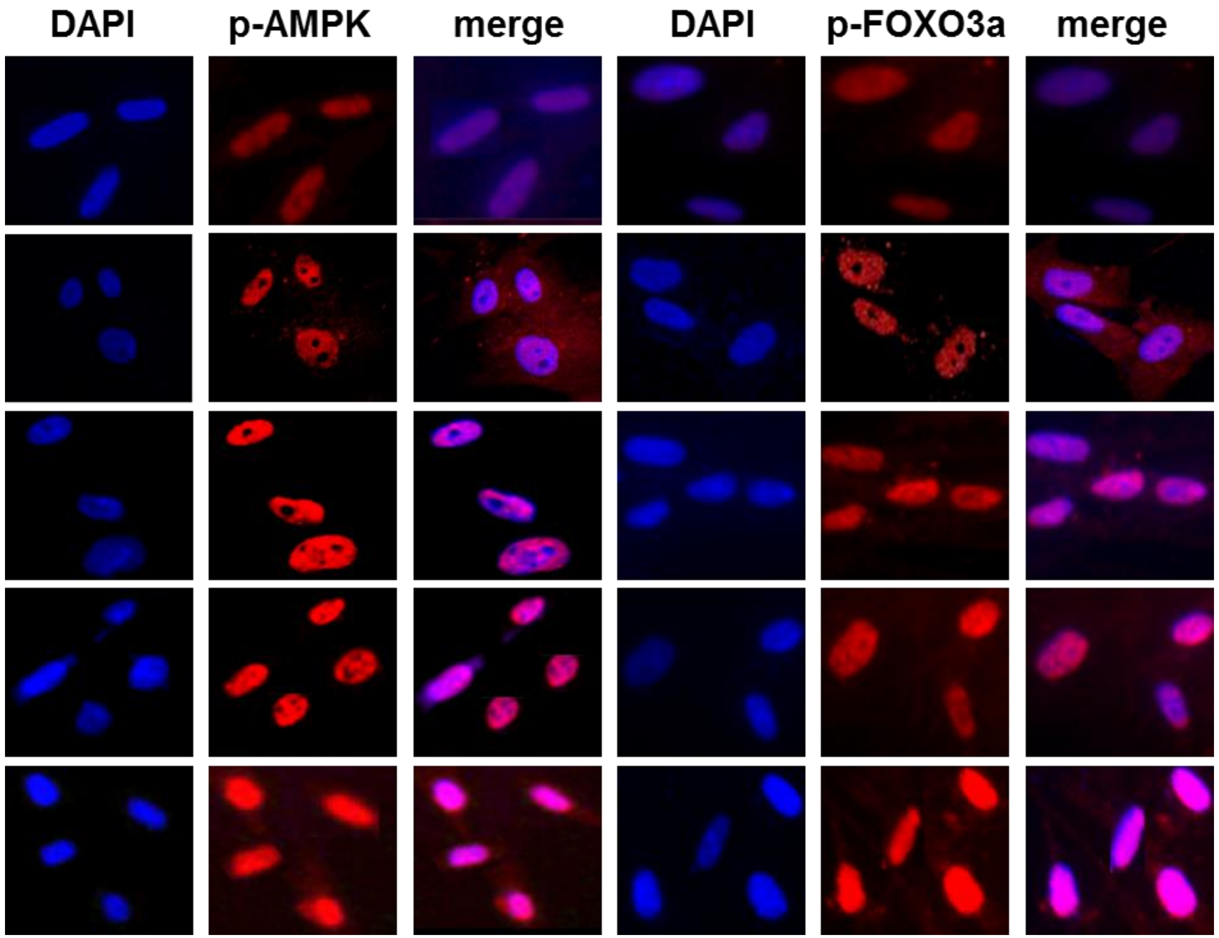

Fig. 2A. Nuclear Transport: Immunofluorescence HDF-2

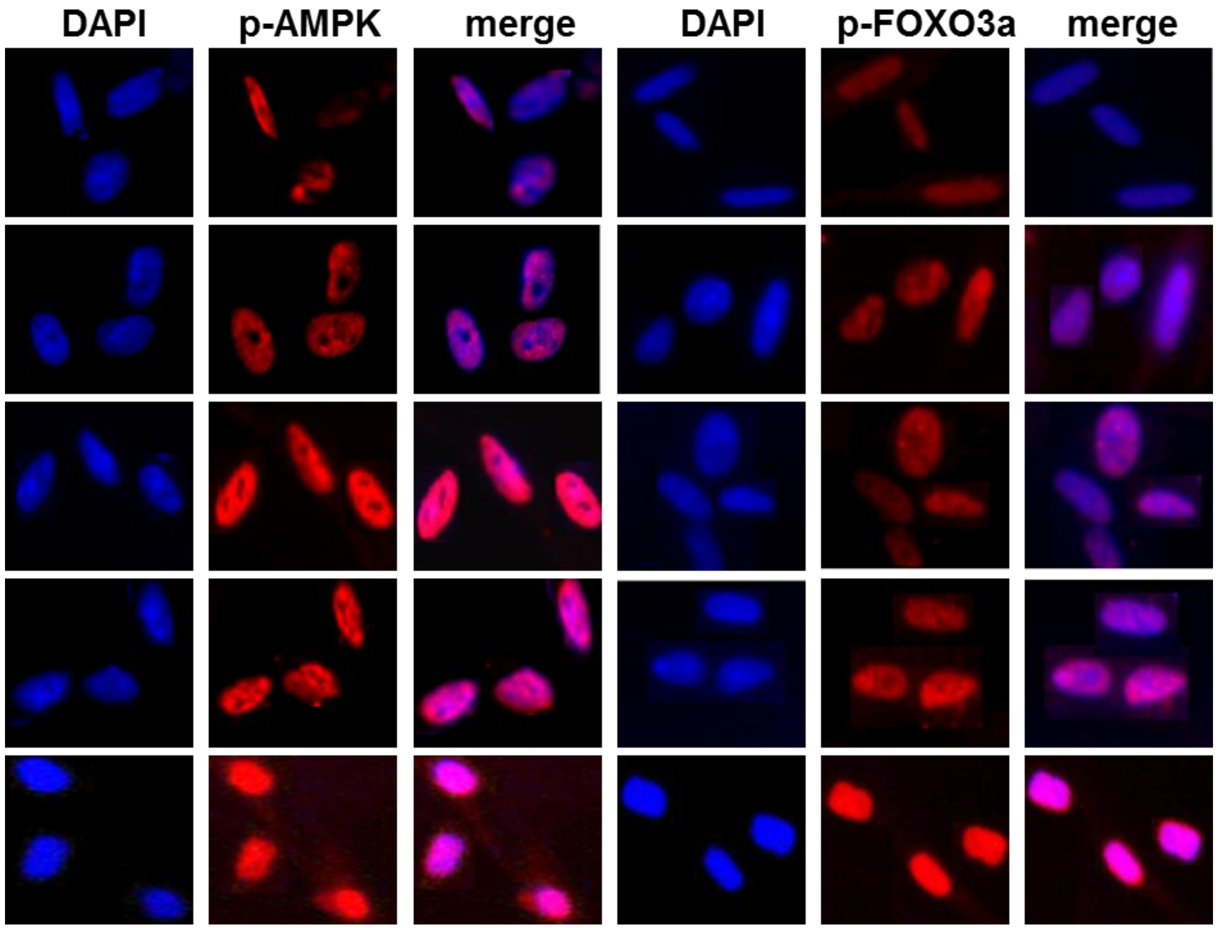

Fig. 2A. Nuclear Transport: Immunofluorescence HDF-3

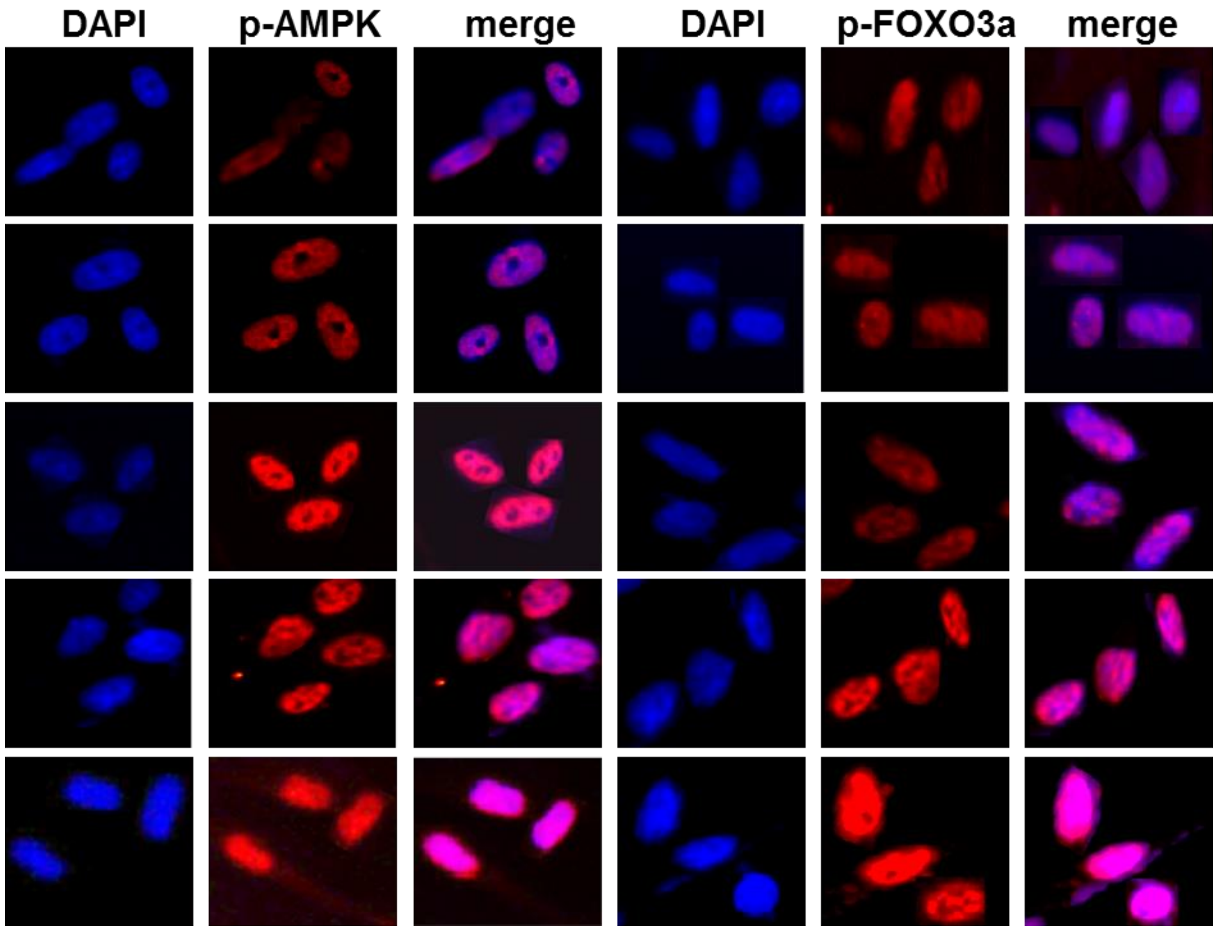

Fig. 2A. Nuclear Transport: Immunofluorescence ASPC-1 -1

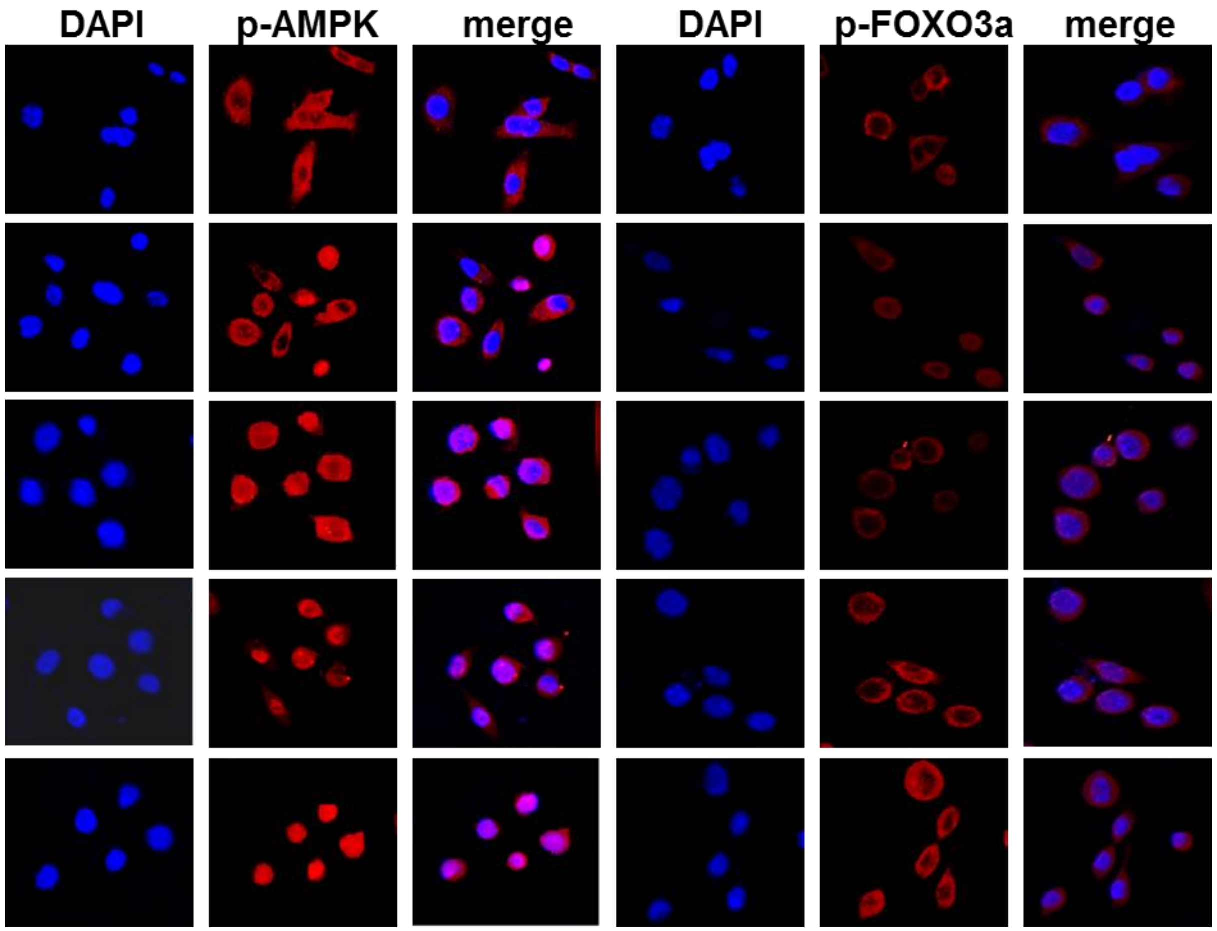

Fig. 2A. Nuclear Transport: Immunofluorescence ASPC-1 -2

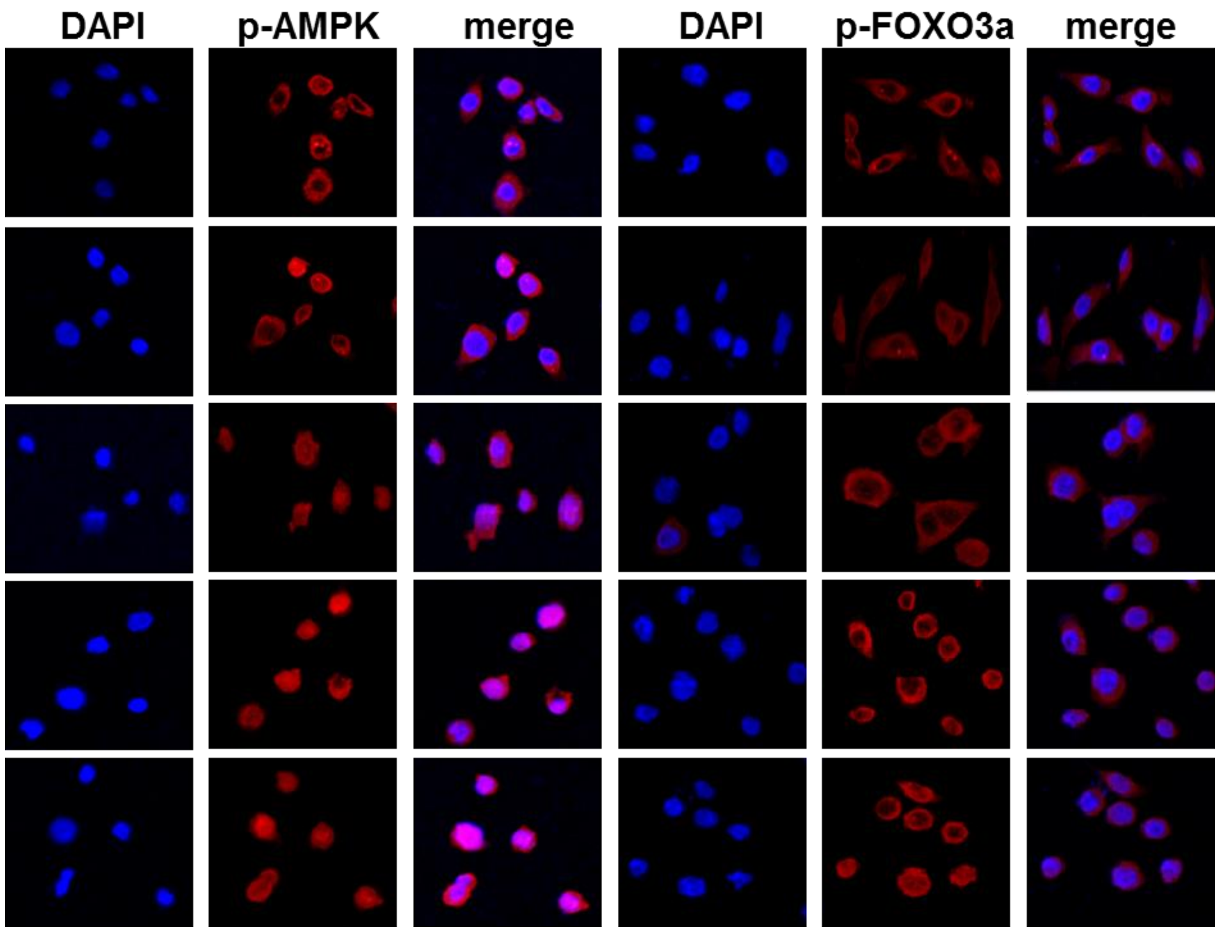

Fig. 2A. Nuclear Transport: Immunofluorescence   ASPC-1 -3

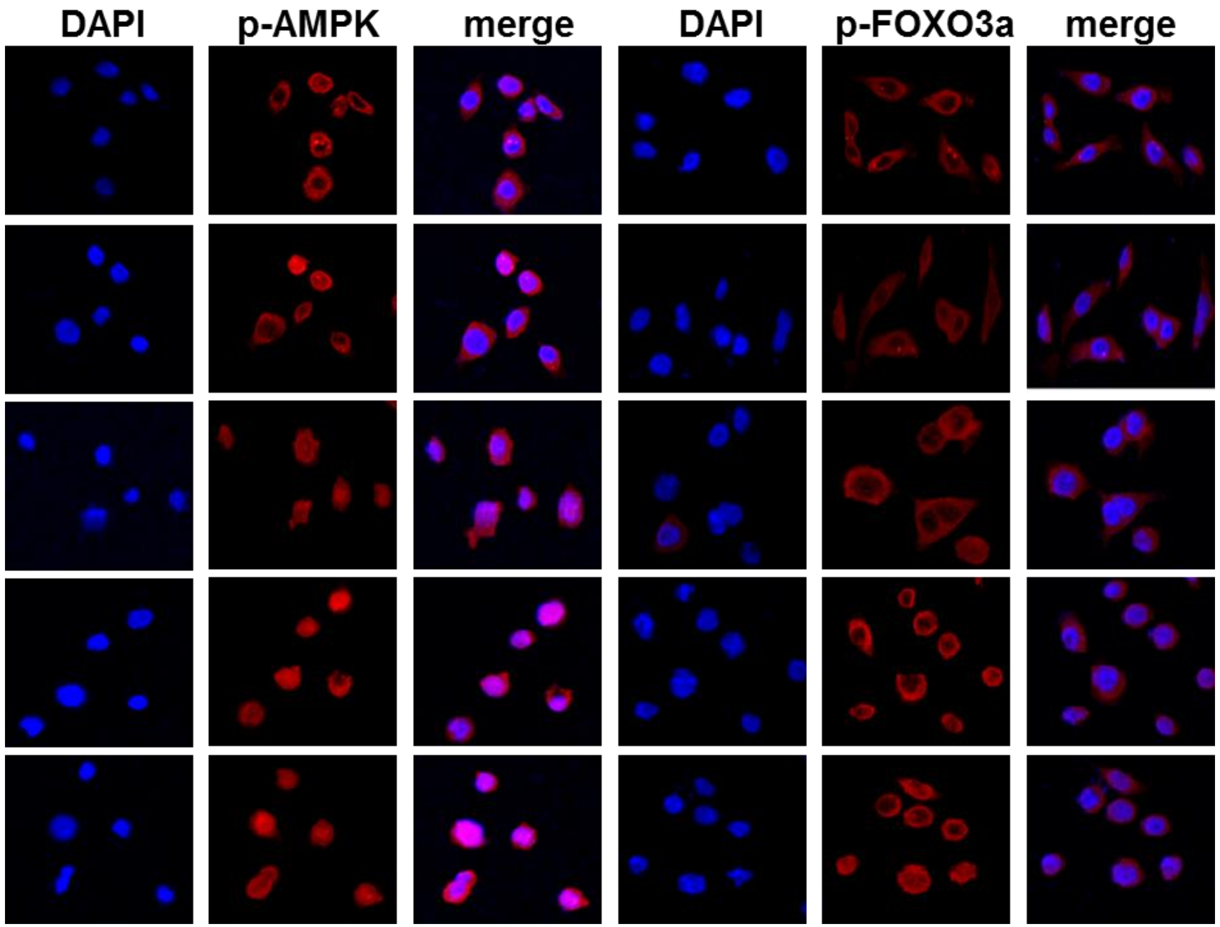

Fig. 2A. Nuclear Transport: Immunofluorescence HDF-1

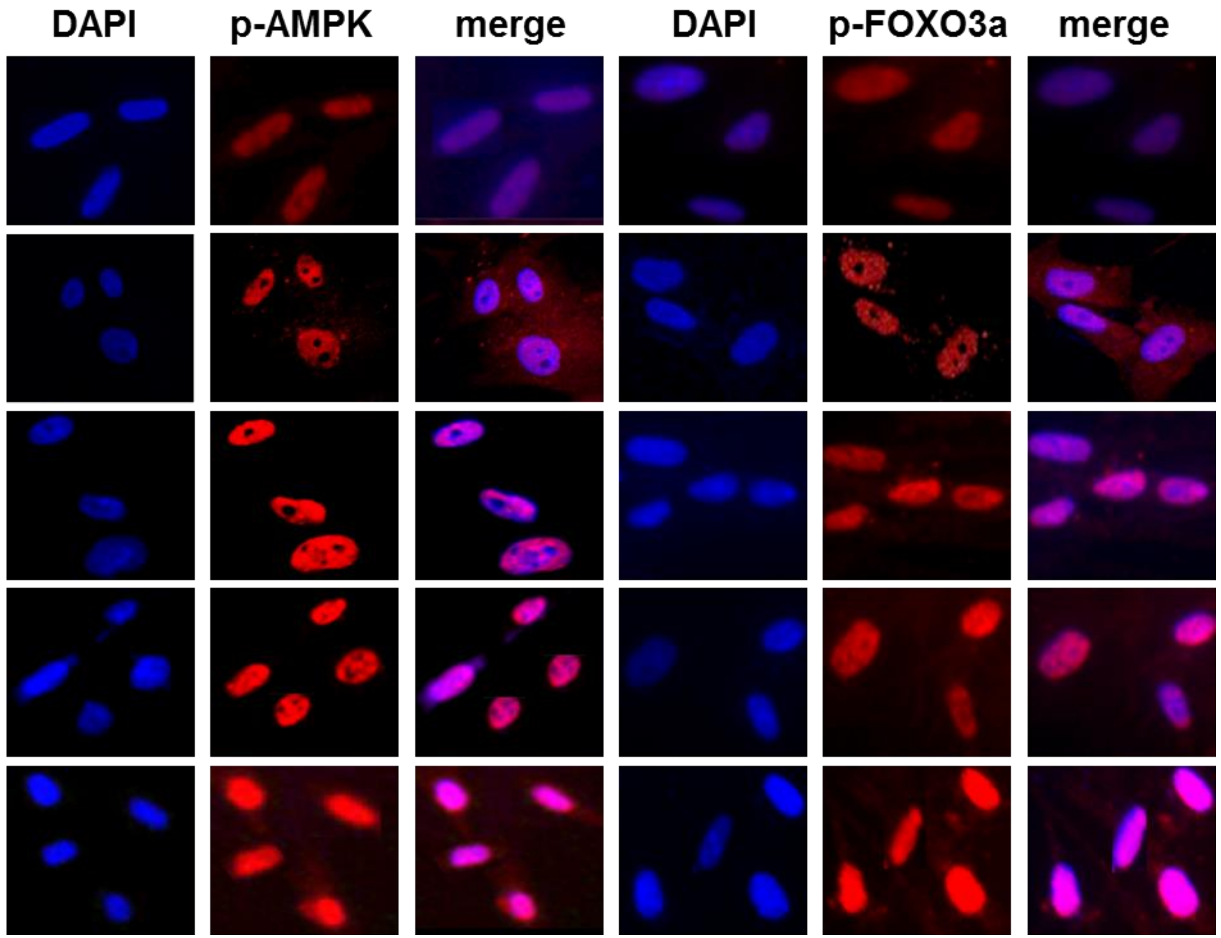

Fig. 2B western blot analysis (HDF)

Ot: other sample

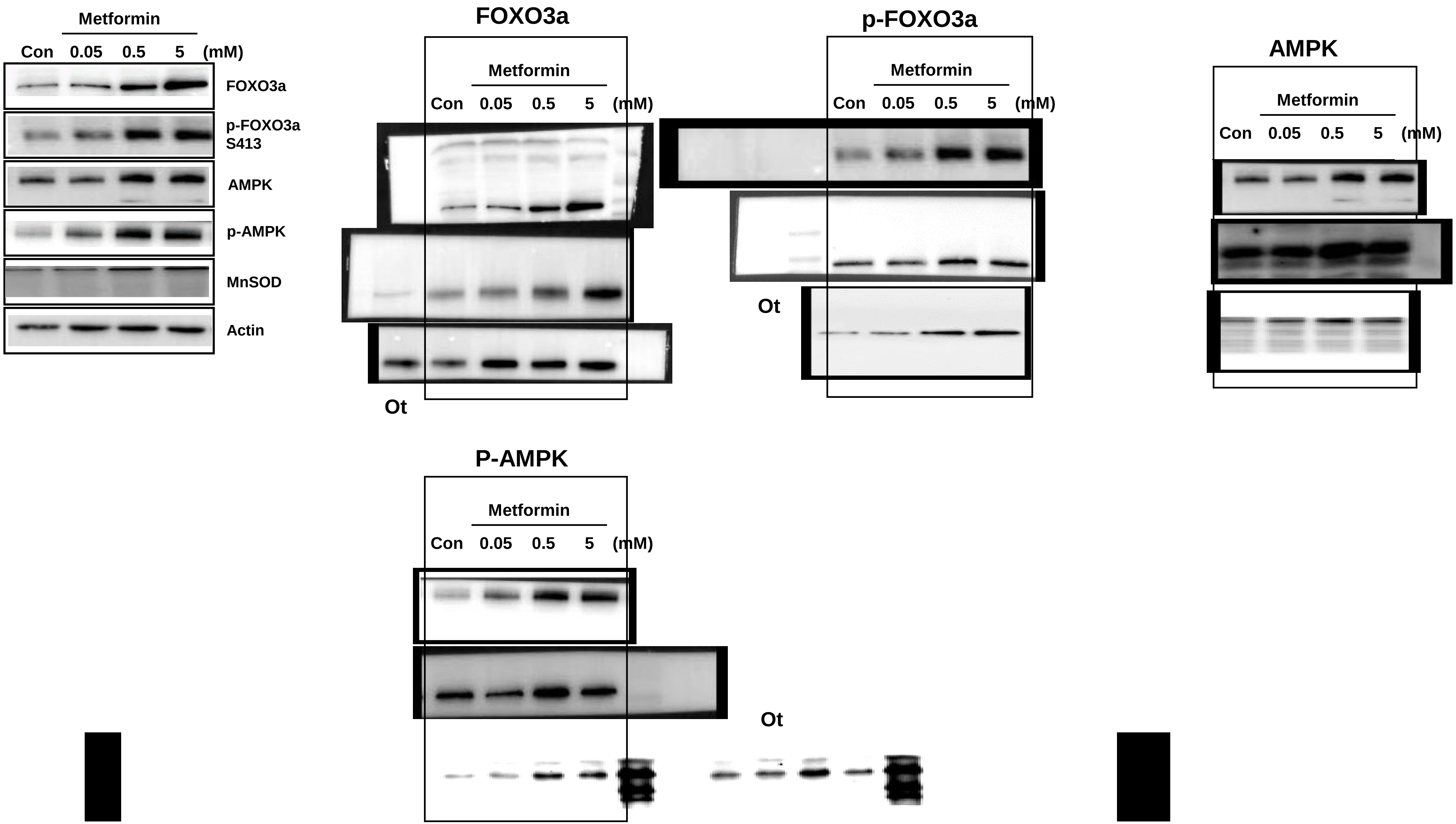

Fig. 2B western blot analysis (HDF)

Ot: other sample

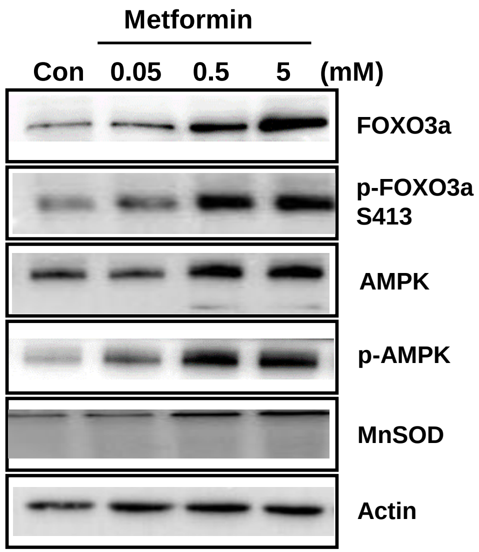

MnSOD

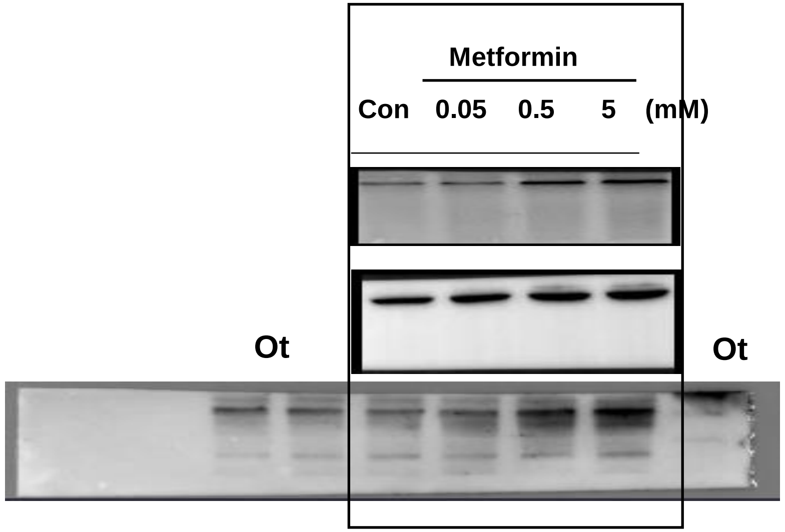

Actin

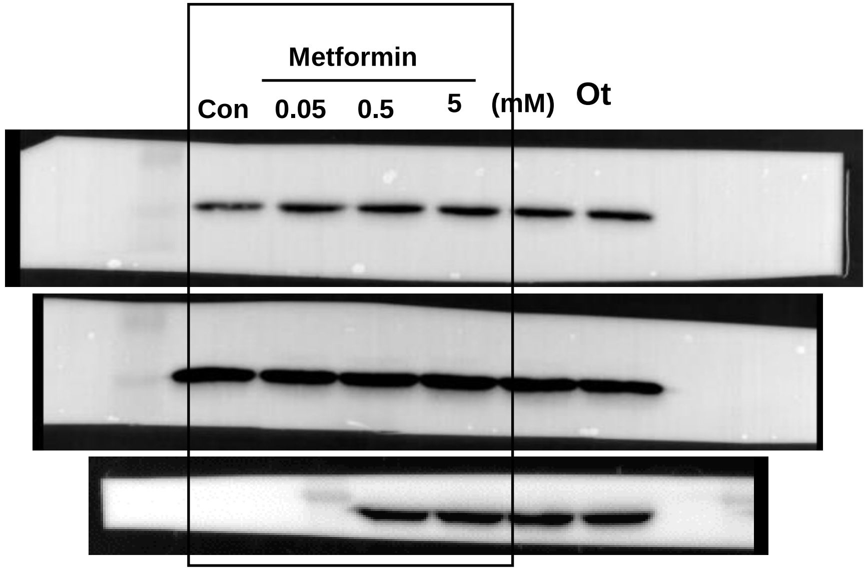

Fig. 2B western blot analysis (AsPC-1)

Ot: other sample

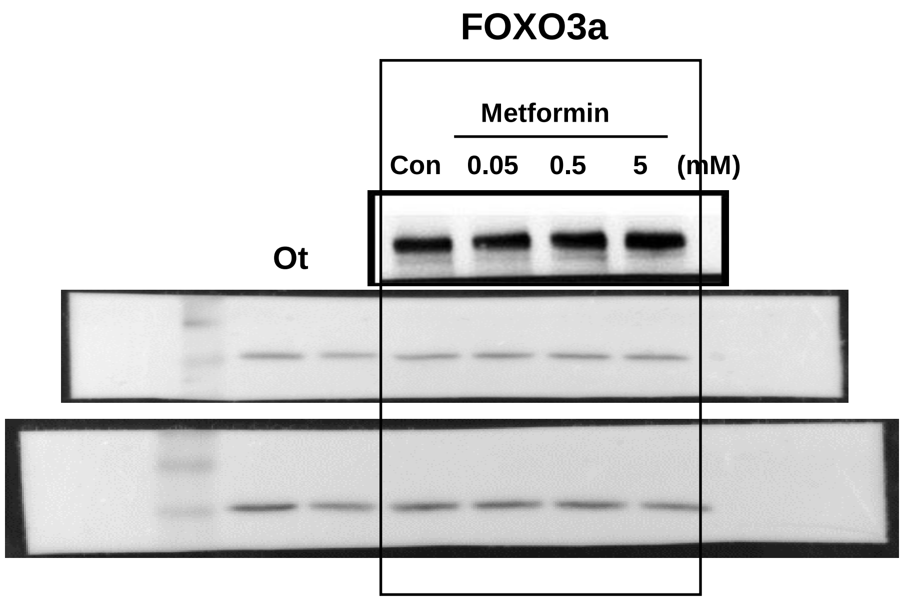

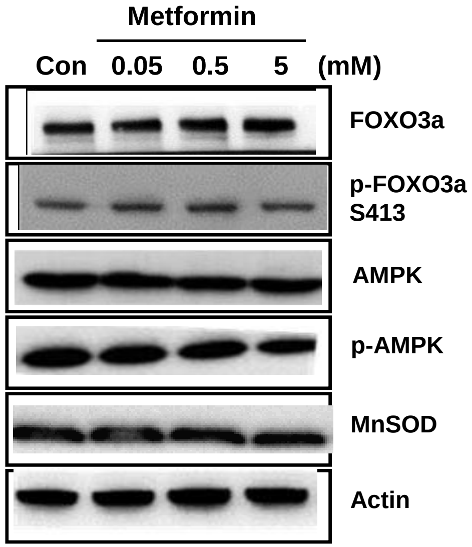

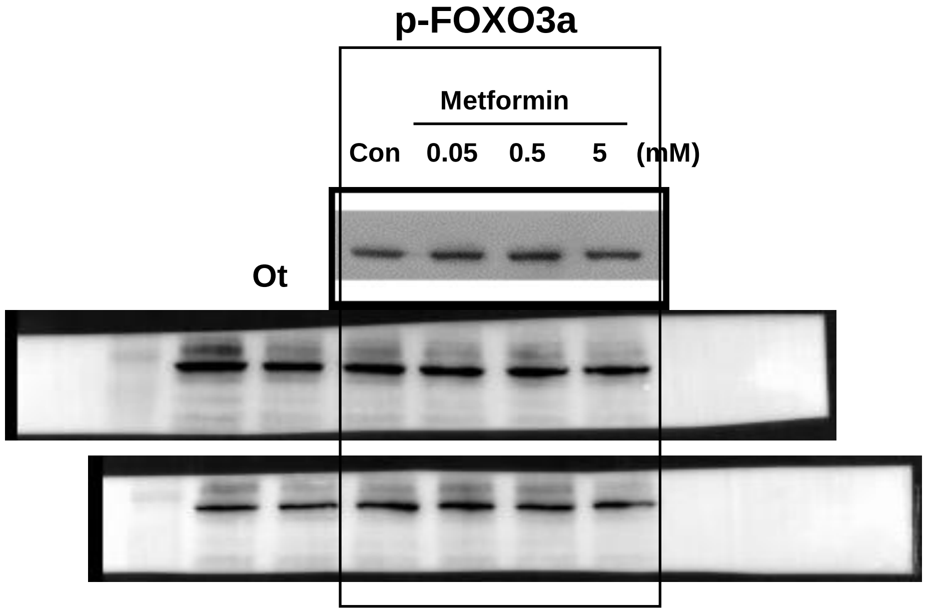

Fig. 2B western blot analysis (AsPC-1)

Ot: other sample

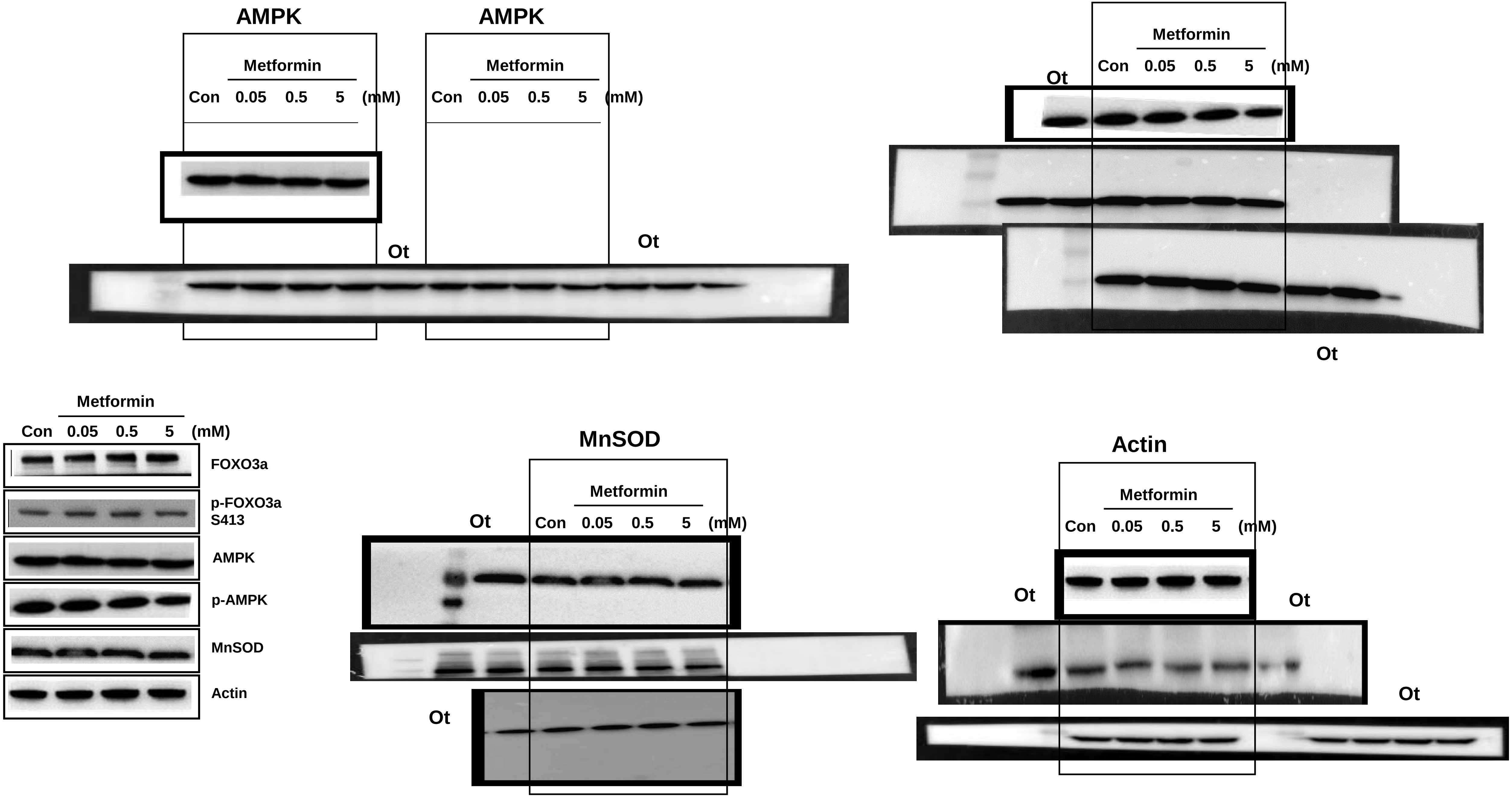

Fig. 3A. si-FOXO3a and wt-FOXO3a transfection by ROS analysis

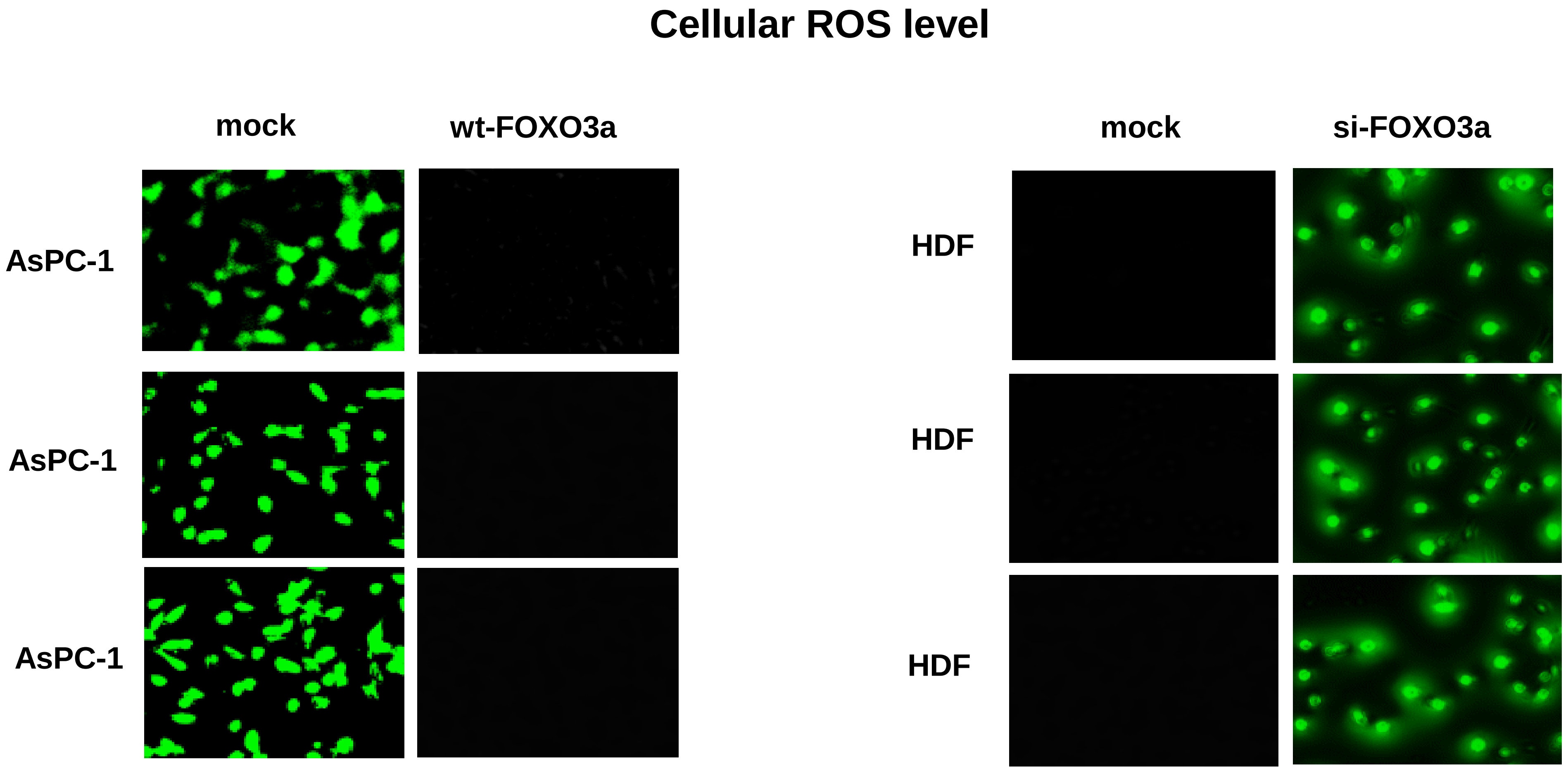

Fig. 3B western blot analysis (AsPC-1 and HDF)

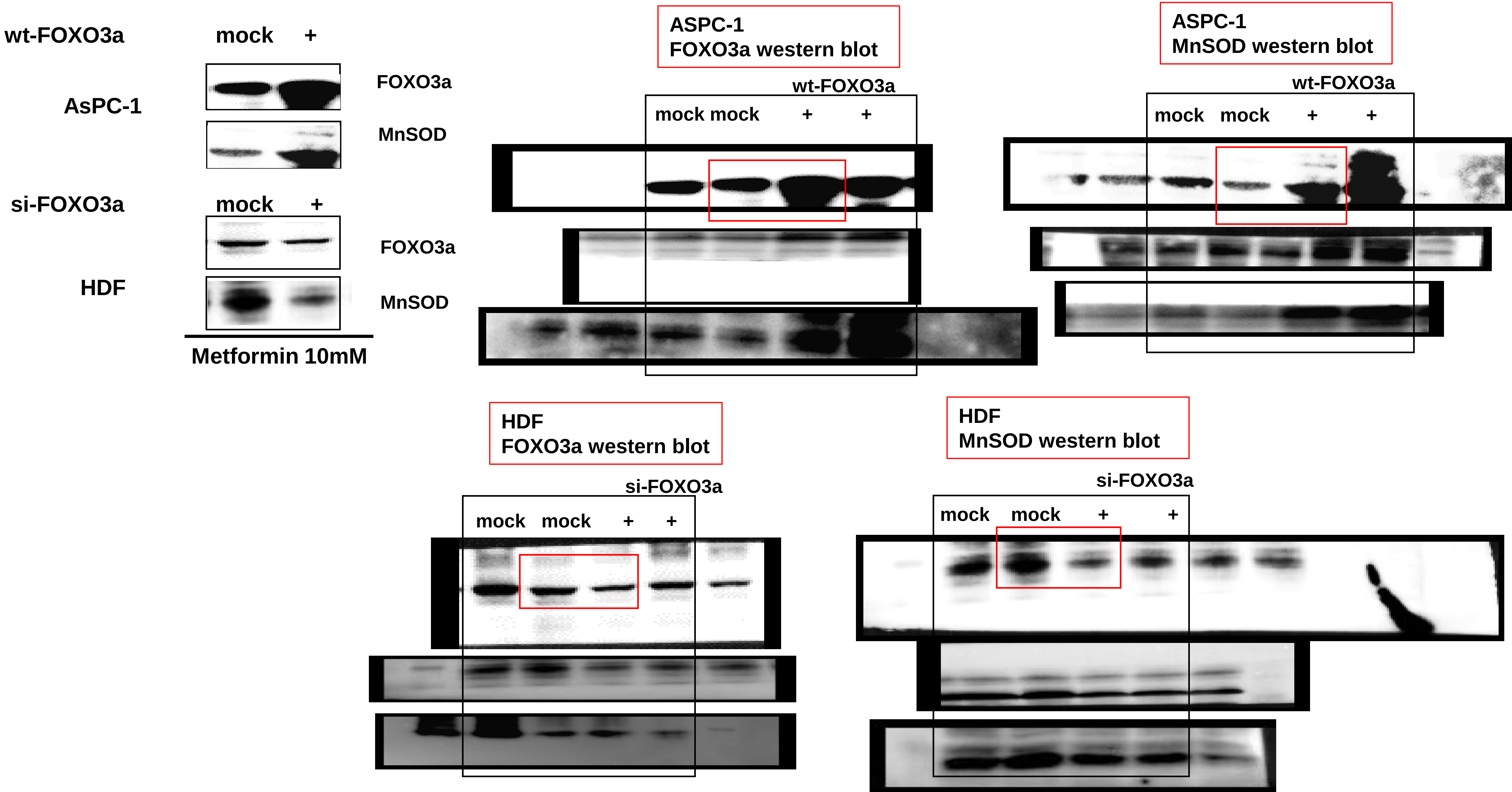

Fig.4B Cell cycle analysis (HDF)

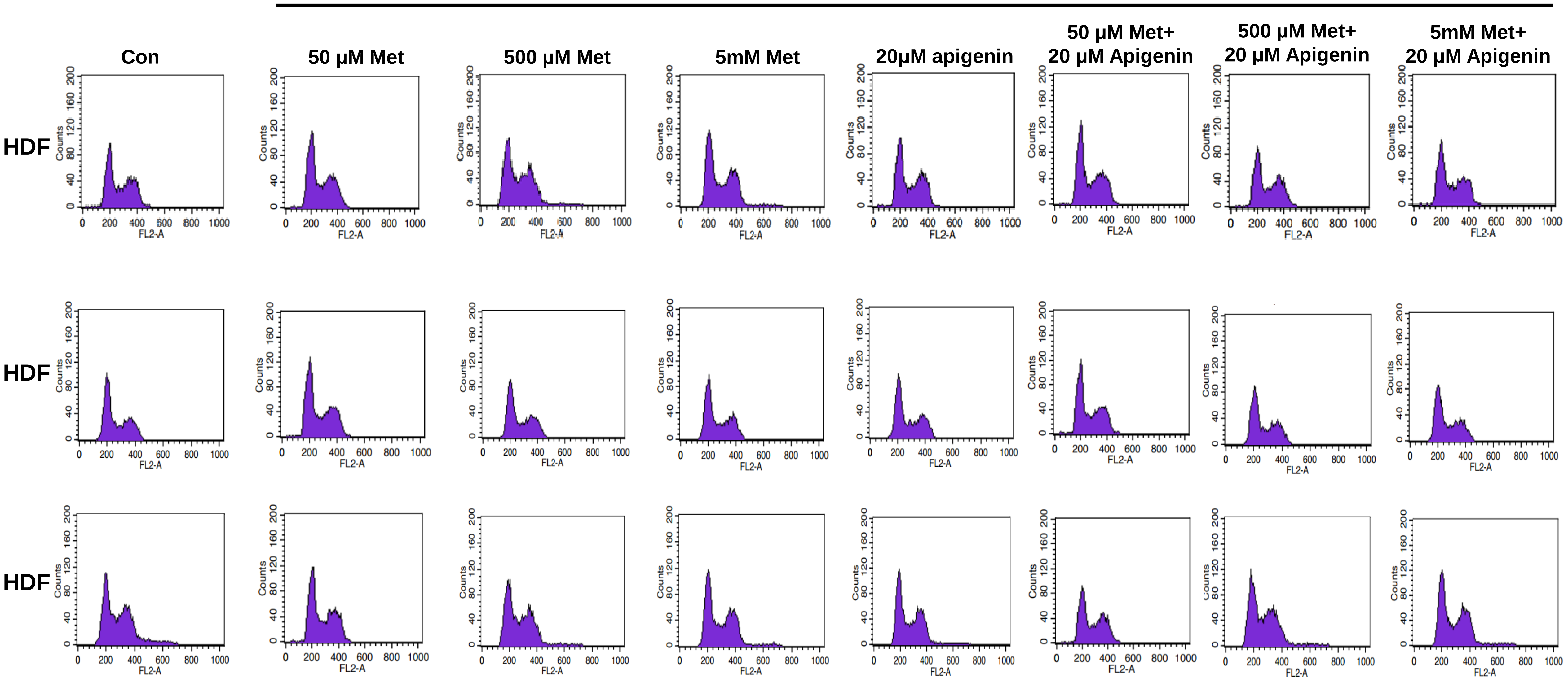

Fig.4 B Cell cycle analysis (AsPC-1)

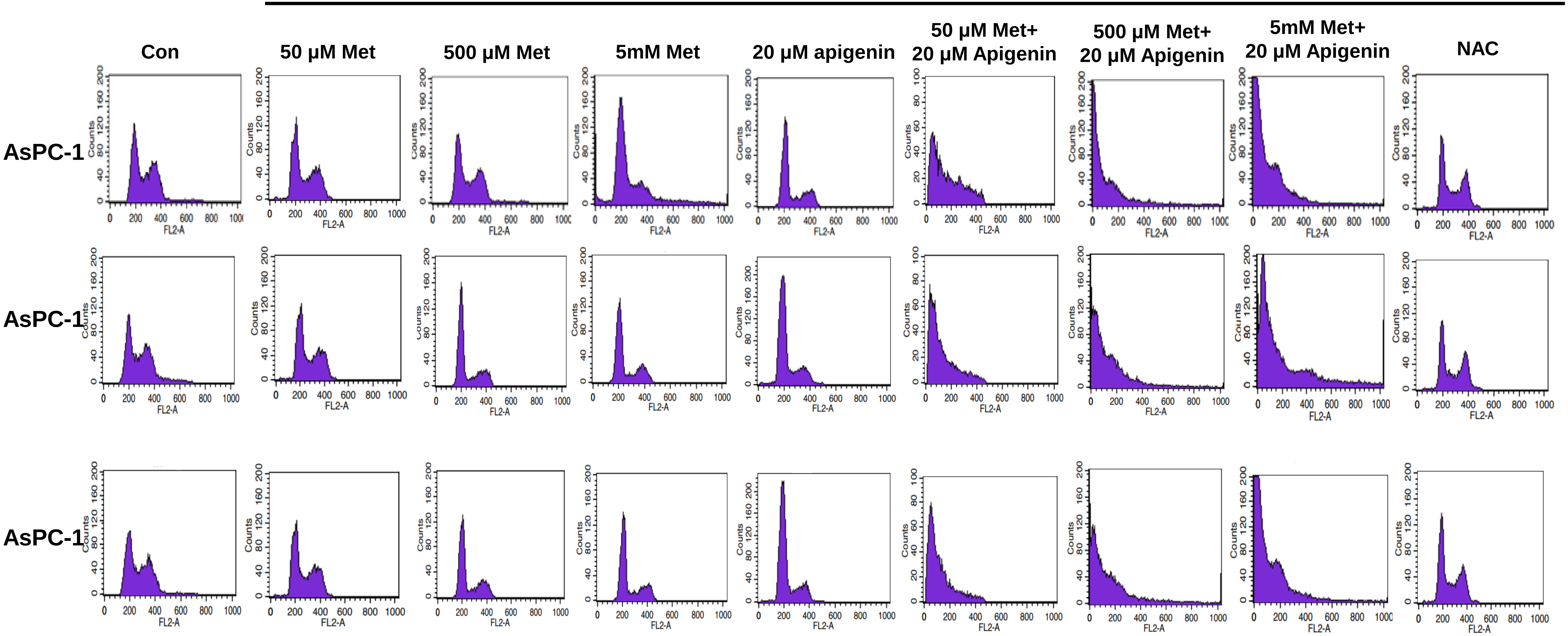

Fig.4 C ROS analysis (HDF)

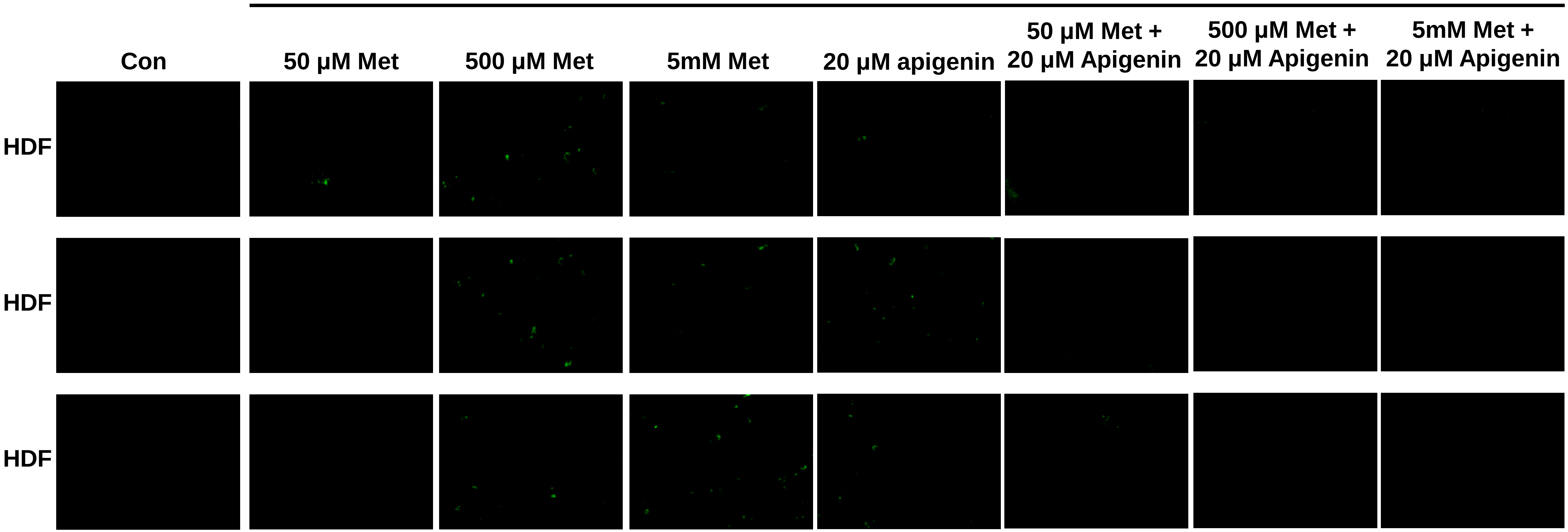

Fig.4 C ROS analysis (AsPC-1)

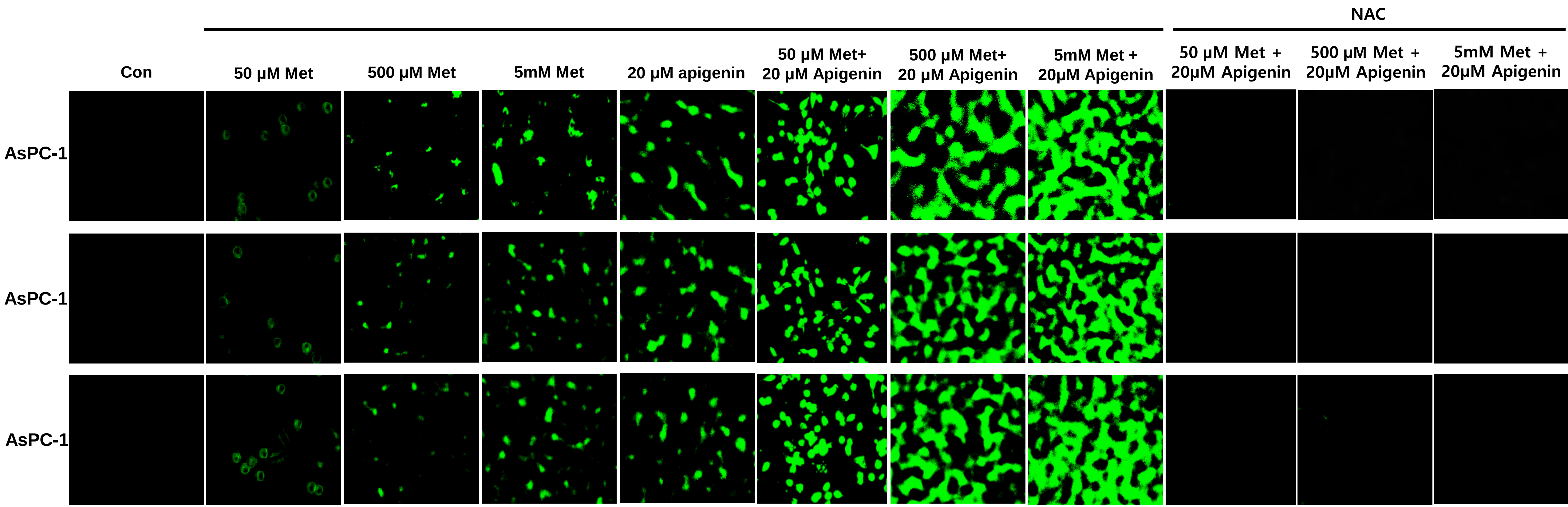

Fig.4 D Membrane potential analysis (AsPC-1)

(D)

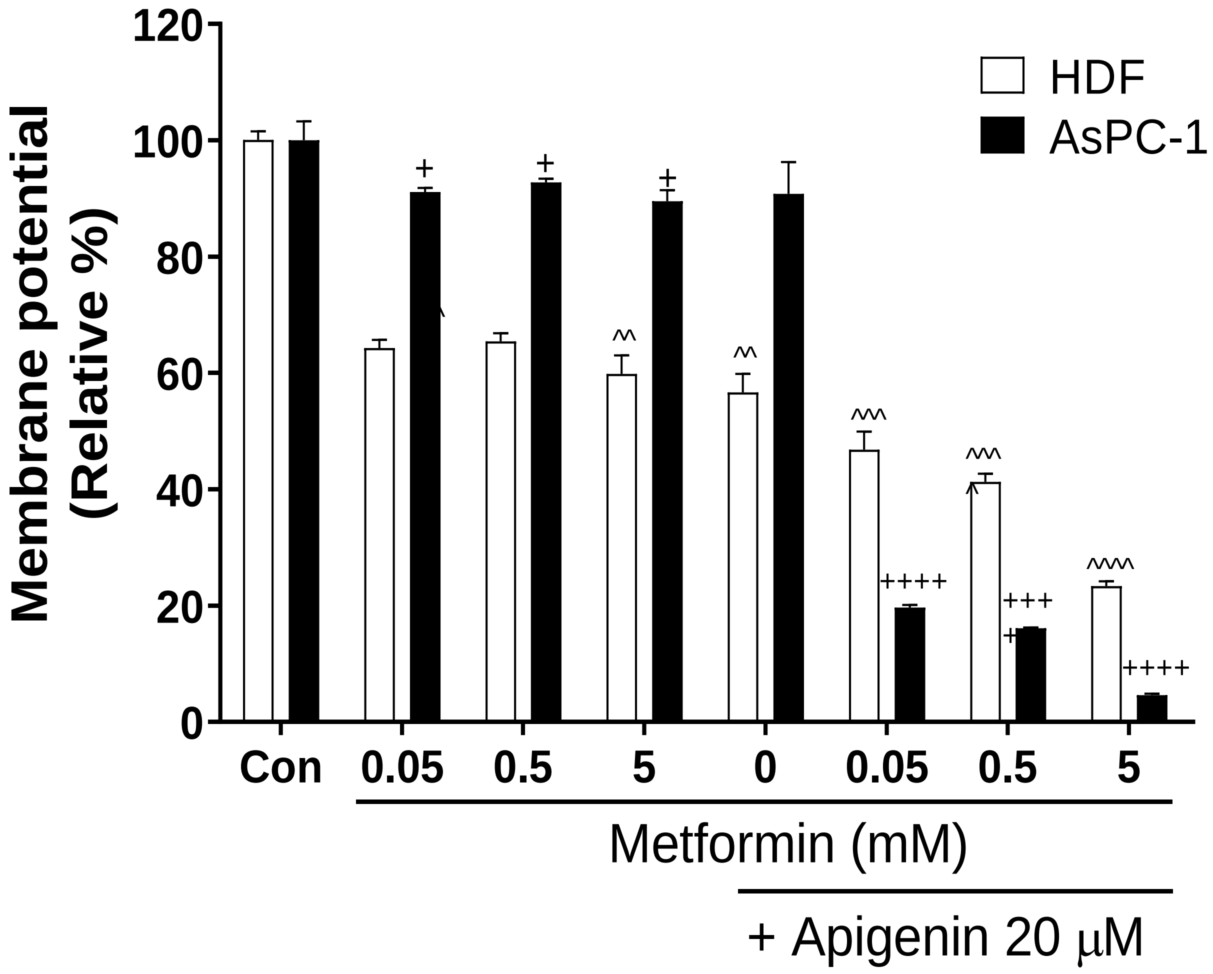

Fig.4 F ROS analysis

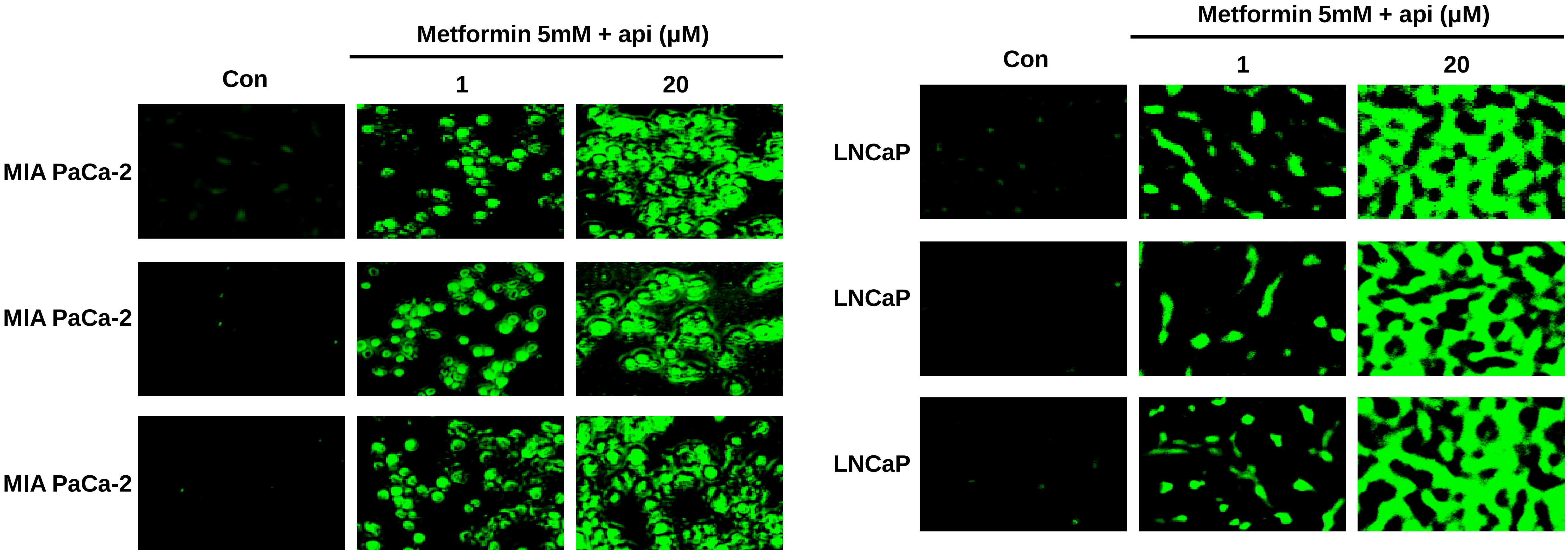

Fig.4 F ROS analysis

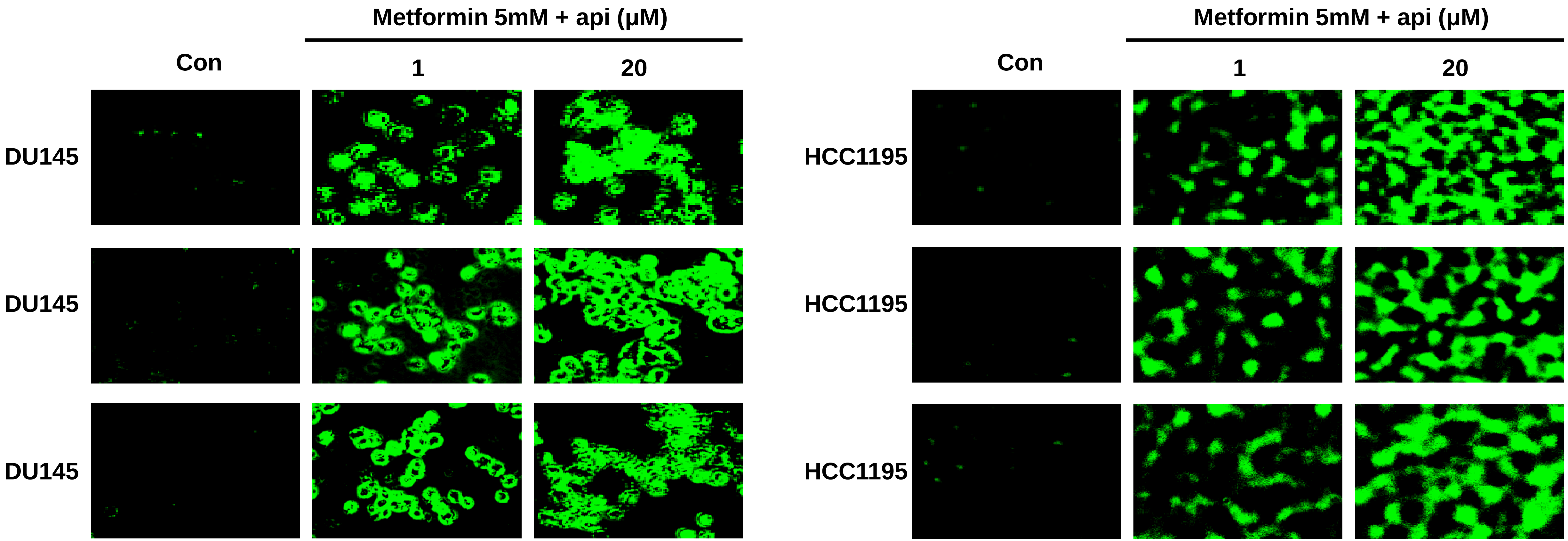

Figure 4 (A) HDF MTT assay

HDF **Metformin+apigenin**

24hrs

Control	0.209	0.216	0.207	0.211
5mM Me	0.219	0.201	0.203	
	(4.762)	5.952	4.762	
1uM Apig	0.211	0.189	0.216	
	0.000	13.690	(2.381)	
20uM apig	0.196	0.191	0.190	
	8.929	11.905	12.500	
5mM Me	0.192	0.199	0.205	
	11.310	7.143	3.571	
5mM Me	0.188	0.189	0.202	
	13.690	13.095	5.357	

Control	(4.762)	5.952	4.762
5mM Me	0.000	13.690	(2.381)
1uM Apig	8.929	11.905	12.500
20uM apig	11.310	7.143	3.571
5mM Me	13.690	13.095	5.357
5mM Met+20uM Apigenin			

HDF **Metformin+apigenin**

48hrs

Control	0.241	0.243	0.237	0.240
	(0.508)	(1.523)	1.523	
5mM Me	0.240	0.219	0.230	
	0.000	10.660	5.076	
1uM Apig	0.228	0.193	0.242	
	6.091	23.858	(1.015)	
20uM apig	0.219	0.215	0.214	
	10.660	12.690	13.198	
5mM Me	0.221	0.223	0.228	
	9.645	8.629	6.091	
5mM Me	0.214	0.216	0.222	
	13.198	12.183	9.137	

Control	(0.508)	(1.523)	1.523
5mM Me	0.000	10.660	5.076
1uM Apig	6.091	23.858	(1.015)
20uM apig	10.660	12.690	13.198
5mM Me	9.645	8.629	6.091
5mM Me	13.198	12.183	9.137

HDF **Metformin+apigenin**

72hrs

Control	0.294	0.300	0.290	0.295
	0.397	(1.984)	1.984	
5mM Me	0.292	0.277	0.284	
	1.190	7.143	4.365	
1uM Apig	0.286	0.249	0.294	
	3.571	18.254	0.397	
20uM apig	0.273	0.268	0.264	
	8.730	10.714	12.302	
5mM Me	0.266	0.264	0.278	
	11.508	12.302	6.746	
5mM Me	0.264	0.256	0.267	
	12.302	15.476	11.111	

Control	0.397	(1.984)	1.984
5mM Me	1.190	7.143	4.365
1uM Apig	3.571	18.254	0.397
20uM apig	8.730	10.714	12.302
5mM Me	11.508	12.302	6.746
5mM Me	12.302	15.476	11.111

HDF **Metformin+apigenin**

96hrs

Control	0.278	0.293	0.273	0.281
	1.261	(5.042)	3.361	
5mM Me	0.265	0.291	0.257	
	6.723	(4.202)	10.084	
1uM Apig	0.292	0.236	0.236	
	(4.622)	18.908	18.908	
20uM apig	0.246	0.227	0.227	
	14.706	22.689	22.689	
5mM Me	0.257	0.257	0.248	
	10.084	10.084	13.866	
5mM Me	0.236	0.239	0.257	
	18.908	17.647	10.084	

Control	1.261	(5.042)	3.361
5mM Me	6.723	(4.202)	10.084
1uM Apig	(4.622)	18.908	18.908
20uM apig	14.706	22.689	22.689
5mM Me	10.084	10.084	13.866
5mM Me	18.908	17.647	10.084

HDF **Metformin+apigenin**

120hrs

Control	0.319	0.328	0.321	0.323
	1.429	(1.786)	0.714	
5mM Me	0.319	0.296	0.307	
	1.429	9.643	5.714	
1uM Apig	0.301	0.273	0.314	
	7.857	17.857	3.214	
20uM apig	0.285	0.290	0.281	
	13.571	11.786	15.000	
5mM Me	0.291	0.299	0.292	
	11.429	8.571	11.071	
5mM Me	0.278	0.271	0.283	
	16.071	18.571	14.286	

Control	1.429	(1.786)	0.714
5mM Me	1.429	9.643	5.714
1uM Apig	7.857	17.857	3.214
20uM apig	13.571	11.786	15.000
5mM Me	11.429	8.571	11.071
5mM Me	16.071	18.571	14.286

Figure 4 (A) AsPC-1 MTT assay

**ASPC-1**	**24 hrs**				Avg					
	Control	0.270	0.258	0.267	0.265		Control	(2.232)	3.125	(0.893)
		(2.232)	3.125	(0.893)			5mM Met	28.571	23.214	24.554
	5mM Met	0.201	0.213	0.210			1uM Apig	13.393	12.500	8.482
		28.571	23.214	24.554			20uM Api	19.196	23.661	21.429
	1uM Apig	0.235	0.237	0.246			5mM Met	28.571	34.821	33.036
		13.393	12.500	8.482			5mM Met	54.464	58.929	56.696
	20uM Api	0.222	0.212	0.217						
		19.196	23.661	21.429						
	5mM Met	0.201	0.187	0.191						
		28.571	34.821	33.036						
	5mM Met	0.143	0.133	0.138						
		54.464	58.929	56.696						
**ASPC-1**	**48 hrs**				Avg		Control	(1.111)	(0.833)	1.667
	Control	0.405	0.404	0.395	0.401		5mM Met	22.222	21.389	21.944
		(1.111)	(0.833)	1.667			1uM Apig	7.778	6.417	5.000
	5mM Met	0.321	0.324	0.322			20uM Api	9.167	8.889	12.500
		22.222	21.389	21.944			5mM Met	40.833	40.278	39.722
	1uM Apig	0.373	0.378	0.383			5mM Met	70.000	68.611	68.611
		7.778	6.417	5.000						
	20uM Api	0.368	0.369	0.356						
		9.167	8.889	12.500						
	5mM Met	0.254	0.256	0.258						
		40.833	40.278	39.722						
	5mM Met	0.149	0.154	0.154						
		70.000	68.611	68.611						

**ASPC-1**	**72hr**				Avg		Control	0.000	(0.000)	(0.000)
	Control	0.454	0.463	0.463	0.462		5mM Met	24.941	24.466	25.178
		0.000	(0.000)	(0.000)			1uM Apig	8.314	9.739	10.214
	5mM Met	0.357	0.359	0.356			20uM Api	19.715	20.428	18.765
		24.941	24.466	25.178			5mM Met	56.532	55.582	56.057
	1uM Apig	0.427	0.421	0.419			5mM Met	86.223	83.848	82.185
		8.314	9.739	10.214						
	20uM Api	0.379	0.376	0.383						
		19.715	20.428	18.765						
	5mM Met	0.224	0.228	0.226						
		56.532	55.582	56.057						
	5mM Met	0.099	0.109	0.116						
		86.223	83.848	82.185						
**ASPC-1**	**96hr**									
					avg		Control	0.000	(0.000)	(0.000)
	Control	0.370	0.373	0.412	0.385		5mM Met	4.360	3.488	(7.849)
		4.360	3.488	(7.849)			1uM Apig	51.163	50.000	49.419
	5mM Met	0.209	0.213	0.215			20uM Api	43.605	42.733	39.535
		51.163	50.000	49.419			5mM Met	46.221	45.930	47.384
	1uM Apig	0.235	0.238	0.249			5mM Met	57.558	58.430	55.233
		43.605	42.733	39.535				100.581	95.058	91.570
	20uM Api	0.226	0.227	0.222						
		46.221	45.930	47.384						
	5mM Met	0.187	0.184	0.195						
		57.558	58.430	55.233						
	5mM Met	0.039	0.058	0.070						
		100.581	95.058	91.570						
**ASPC-1**	**######**				avg		Control	2.863	(1.542)	(1.322)
	Control	0.481	0.501	0.500	0.494		5mM Met	31.567	32.009	31.567
		2.863	(1.542)	(1.322)			1uM Apig	18.764	18.985	19.205
	5mM Met	0.351	0.349	0.351			20uM Api	27.373	26.711	28.698
		31.567	32.009	31.567			5mM Met	64.018	61.810	60.265
	1uM Apig	0.409	0.408	0.407			5mM Met	99.558	98.675	99.117
		18.764	18.985	19.205						
	20uM Api	0.370	0.373	0.364						
		27.373	26.711	28.698						
	5mM Met	0.204	0.214	0.221						
		64.018	61.810	60.265						
	5mM Met	0.043	0.047	0.045						
		99.558	98.675	99.117						

Figure 4. D) Mitochondrial membrane potentia AsPC-1 and HDF cells

ASPC-1 Membrane potential					
Control	0.5mM Metfromin	5mM Metformin	20uM Apigenin	0.5mM Metformin+20uM Apigenin	5mM Metformin+20uM Apigenin
0.9	0.89	0.89	0.78	0.153	0.041
0. 991	0.87	0.867	0.962	0.155	0.049
0.995	0.877	0.827	0.879	0.157	0.043
HDF Membrane potential					
Control	0.5mM Metfromin	5mM Metformin	20uM Apigenin	0.5mM Metformin+20uM Apigenin	5mM Metformin+20uM Apigenin
0.272	0.181	0.175	0.162	0.112	0.064
0.285	0.188	0.174	0.14	0.122	0.069
0.275	0.175	0.148	0.169	0.109	0.061

Figure 4 (F) DU145 MTT assay

**DU145**	**24 hrs**				Avg							
	Control	0.266	0.251	0.254	0.257	Control	103.502	97.665	98.833	Control	5mM Met	5mM Met+20uM Apigenin
		103.502	97.665	98.833		5mM Met	68.872	73.541	72.374	103.502	68.872	46.304
	5mM Met	0.177	0.189	0.186		5mM Met	46.304	50.973	49.805	97.665	73.541	50.973
		68.872	73.541	72.374						98.833	72.374	49.805
	5mM Met	0.119	0.131	0.128								
		46.304	50.973	49.805								
**DU145**	**48 hrs**				Avg	Control	97.800	101.222	100.978	Control	5mM Met	5mM Met+20uM Apigenin
	Control	0.400	0.414	0.413	0.409	5mM Met	59.413	63.081	62.347	97.800	59.413	36.919
		97.800	101.222	100.978		5mM Met	36.919	38.142	38.142	101.222	63.081	38.142
	5mM Met	0.243	0.258	0.255						100.978	62.347	38.142
		59.413	63.081	62.347								
	5mM Met	0.151	0.156	0.156								
		36.919	38.142	38.142								
**DU145**	**72hr**				Avg	Control	98.253	100.218	101.528	Control	5mM Met	5mM Met+20uM Apigenin
	Control	0.450	0.459	0.465	0.458	5mM Met	46.070	44.323	48.472	98.253	46.070	20.742
		98.253	100.218	101.528		5mM Met	20.742	22.926	24.454	100.218	44.323	22.926
	5mM Met	0.211	0.203	0.222						101.528	48.472	24.454
		46.070	44.323	48.472								
	5mM Met	0.095	0.105	0.112								
		20.742	22.926	24.454								
**DU145**	**96hr**				Avg							
	Control	0.487	0.516	0.518	0.507	Control	96.055	101.775	102.170	Control	5mM Met	5mM Met+20uM Apigenin
		96.055	101.775	102.170		5mM Met	25.838	28.994	28.600	96.055	25.838	6.114
	5mM Met	0.131	0.147	0.145		5mM Met	6.114	6.706	14.398	101.775	28.994	6.706
		25.838	28.994	28.600						102.170	28.600	14.398
	5mM Met	0.031	0.034	0.073								
		6.114	6.706	14.398								

Figure 4 (F) HCCC1195 MTT assay

**HCC1195**	**24 hrs**				Avg										
	Control	0.264	0.276	0.273	0.271		Control	97.417	101.845	100.738			Control	5mM Met	5mM Met+20uM Apigenin
		97.417	101.845	100.738			5mM Met	72.694	67.159	68.266			97.417	72.694	49.077
	5mM Met	0.197	0.182	0.185			5mM Met	49.077	48.339	47.601			101.845	67.159	48.339
		72.694	67.159	68.266									100.738	68.266	47.601
	5mM Met	0.133	0.131	0.129											
		49.077	48.339	47.601											
**HCC1195**	**48 hrs**				Avg		Control	97.789	101.229	100.983			Control	5mM Met	5mM Met+20uM Apigenin
	Control	0.398	0.412	0.411	0.407		5mM Met	59.459	63.145	62.408			97.789	59.459	36.118
		97.789	101.229	100.983			5mM Met	36.118	36.609	37.101			101.229	63.145	36.609
	5mM Met	0.242	0.257	0.254									100.983	62.408	37.101
		59.459	63.145	62.408											
	5mM Met	0.147	0.149	0.151											
		36.118	36.609	37.101											
**HCC1195**	**72hr**				Avg		Control	98.265	100.217	101.518			Control	5mM Met	5mM Met+20uM Apigenin
	Control	0.453	0.462	0.468	0.461		5mM Met	48.807	47.072	51.193			98.265	48.807	22.560
		98.265	100.217	101.518			5mM Met	22.560	24.729	26.247			100.217	47.072	24.729
	5mM Met	0.225	0.217	0.236									101.518	51.193	26.247
		48.807	47.072	51.193											
	5mM Met	0.104	0.114	0.121											
		22.560	24.729	26.247											
**HCC1195**	**96hr**				Avg										
	Control	0.479	0.508	0.510	0.499		Control	95.992	101.804	102.204			Control	5mM Met	5mM Met+20uM Apigenin
		95.992	101.804	102.204			5mM Met	31.463	34.669	34.269			95.992	31.463	9.619
	5mM Met	0.157	0.173	0.171			5mM Met	9.619	10.220	18.036			101.804	34.669	10.220
		31.463	34.669	34.269									102.204	34.269	18.036
	5mM Met	0.048	0.051	0.090											
		9.619	10.220	18.036											

Figure 4 (F) LNCaP MTT assay

**LNCaP**	**24 hrs**				Avg											
	Control	0.254	0.239	0.242	0.245		Control	103.673	97.551	98.776		Control	5mM Met	5mM Met+20uM Apigenin		
		103.673	97.551	98.776			5mM Met	64.490	66.531	66.122		103.673	64.490	52.245		
	5mM Met	0.158	0.163	0.162			5mM Met	52.245	48.163	50.204		97.551	66.531	48.163		
		64.490	66.531	66.122								98.776	66.122	50.204		
	5mM Met	0.128	0.118	0.123												
		52.245	48.163	50.204												
**LNCaP**	**48 hrs**				Avg		Control	101.376	98.538	100.086		Control	5mM Met	5mM Met+20uM Apigenin		
	Control	0.393	0.382	0.388	0.388		5mM Met	47.721	51.591	50.817		101.376	47.721	26.053		
		101.376	98.538	100.086			5mM Met	26.053	27.859	28.375		98.538	51.591	27.859		
	5mM Met	0.185	0.200	0.197								100.086	50.817	28.375		
		47.721	51.591	50.817												
	5mM Met	0.101	0.108	0.110												
		26.053	27.859	28.375												
**LNCaP**	**72hr**				Avg		Control	98.313	101.687	100.000		Control	5mM Met	5mM Met+20uM Apigenin		
	Control	0.408	0.422	0.415	0.415		5mM Met	34.458	32.530	37.108		98.313	34.458	9.157		
		98.313	101.687	100.000			5mM Met	9.157	12.530	11.566		101.687	32.530	12.530		
	5mM Met	0.143	0.135	0.154								100.000	37.108	11.566		
		34.458	32.530	37.108												
	5mM Met	0.038	0.052	0.048												
		9.157	12.530	11.566												
**LNCaP**	**96hr**				Avg											
	Control	0.403	0.406	0.445	0.418		Control	96.411	97.129	106.459		Control	96.411	97.129	106.459	
		96.411	97.129	106.459			5mM Met	32.057	35.885	35.407		5mM Met	32.057	35.885	35.407	
	5mM Met	0.134	0.150	0.148			5mM Met	8.852	11.244	12.919		5mM Met	8.852	11.244	12.919	
		32.057	35.885	35.407												
	5mM Met	0.037	0.047	0.054												
		8.852	11.244	12.919												

Figure 4 (F) MIA PaCa-2 MTT assay

**MIA PaC**	**24 hrs**				Avg							
	Control	0.264	0.276	0.273	0.271	Control	97.417	101.845	100.738	Control	5mM Met	5mM Met+20uM Apigenin
		97.417	101.845	100.738		5mM Met	66.052	62.362	64.207	97.417	66.052	50.185
	5mM Met	0.179	0.169	0.174		5mM Met	50.185	54.613	53.506	101.845	62.362	54.613
		66.052	62.362	64.207						100.738	64.207	53.506
	5mM Met	0.136	0.148	0.145								
		50.185	54.613	53.506								
**MIA PaC**	**48 hrs**				Avg	Control	99.190	100.405	100.405	Control	5mM Met	5mM Met+20uM Apigenin
	Control	0.408	0.413	0.413	0.411	5mM Met	54.700	55.429	58.347	99.190	54.700	38.169
		99.190	100.405	100.405		5mM Met	38.169	37.925	35.737	100.405	55.429	37.925
	5mM Met	0.225	0.228	0.240						100.405	58.347	35.737
		54.700	55.429	58.347								
	5mM Met	0.157	0.156	0.147								
		38.169	37.925	35.737								
**MIA PaC**	**72hr**				Avg	Control	99.784	98.060	102.155	Control	5mM Met	5mM Met+20uM Apigenin
	Control	0.463	0.455	0.474	0.464	5mM Met	46.983	47.845	47.414	99.784	46.983	23.922
		99.784	98.060	102.155		5mM Met	23.922	26.078	27.586	98.060	47.845	26.078
	5mM Met	0.218	0.222	0.220						102.155	47.414	27.586
		46.983	47.845	47.414								
	5mM Met	0.111	0.121	0.128								
		23.922	26.078	27.586								
**MIA PaC**	**96hr**				Avg							
	Control	0.486	0.489	0.528	0.501	Control	97.006	97.605	105.389	Control	5mM Met	5mM Met+20uM Apigenin
		97.006	97.605	105.389		5mM Met	29.541	32.735	32.335	97.006	29.541	13.174
	5mM Met	0.148	0.164	0.162		5mM Met	13.174	17.166	16.966	97.605	32.735	17.166
		29.541	32.735	32.335						105.389	32.335	16.966
	5mM Met	0.066	0.086	0.085								
		13.174	17.166	16.966								

Fig.5 A western blot analysis (HDF)

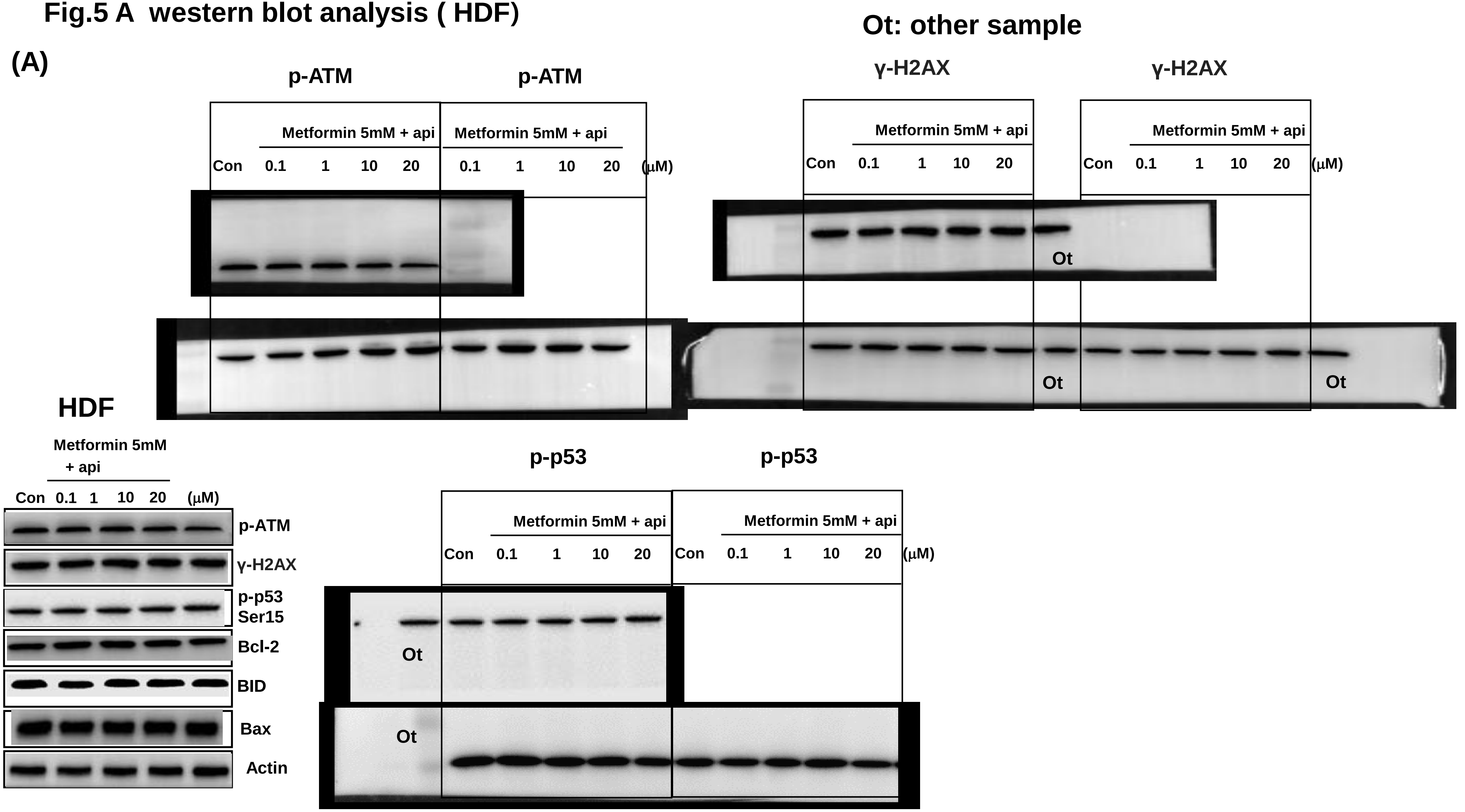

Fig.5 A western blot analysis (HDF)

Ot: other sample

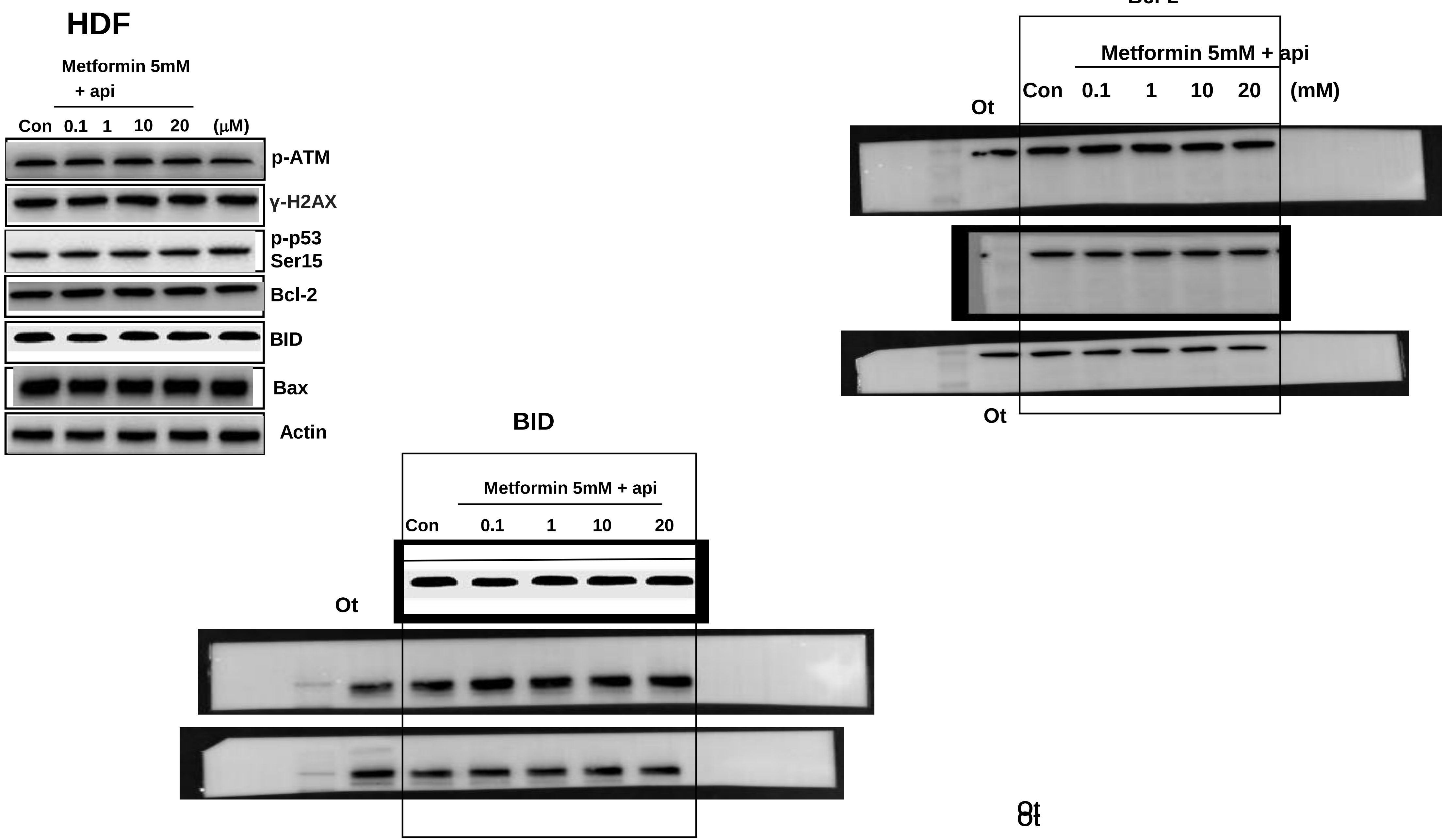

Fig.5 A western blot analysis (HDF)

Ot: other sample

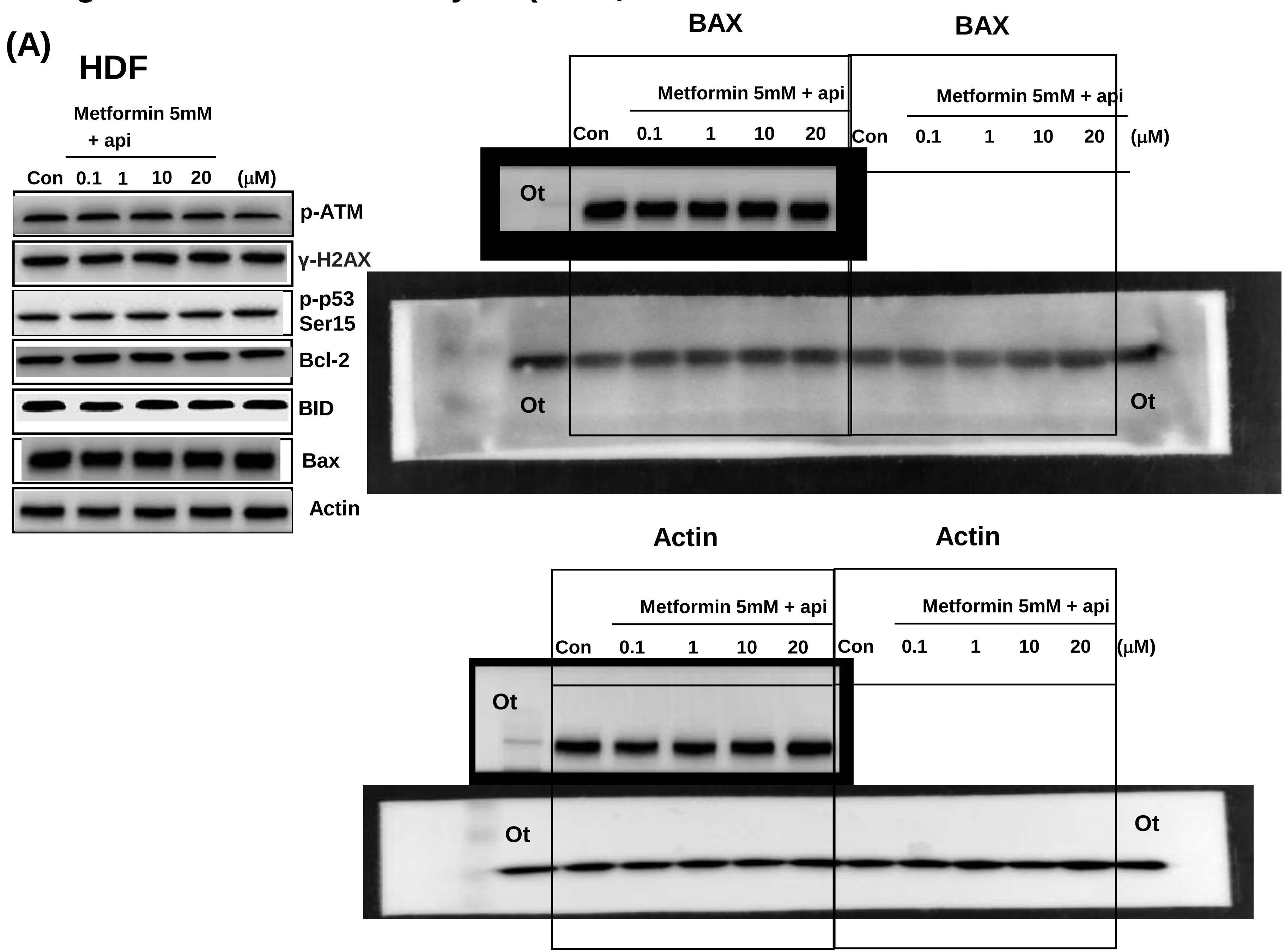

Fig.5 A western blot analysis (AsPC-1)

Ot: other sample

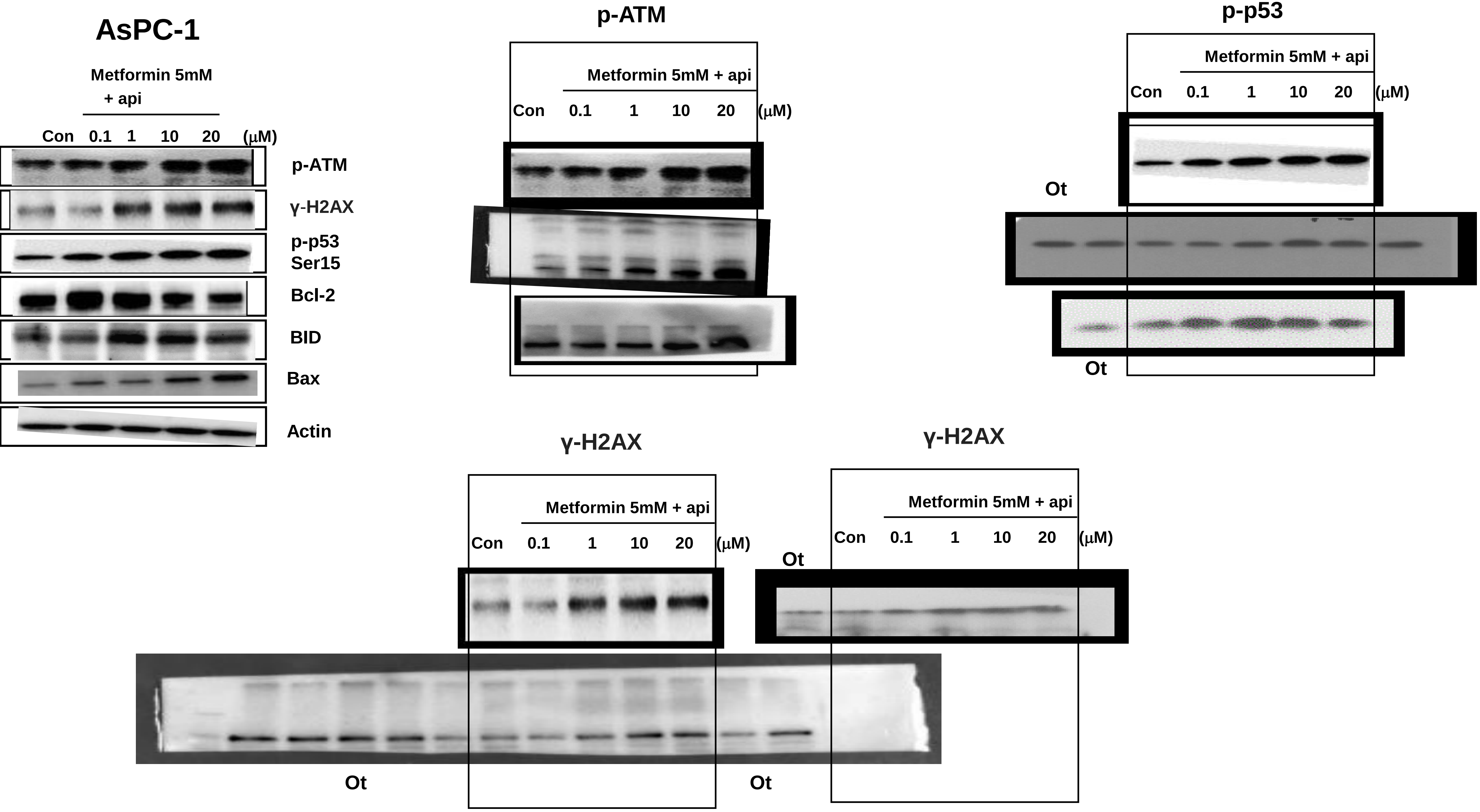

Fig.5 A western blot analysis (AsPC-1)

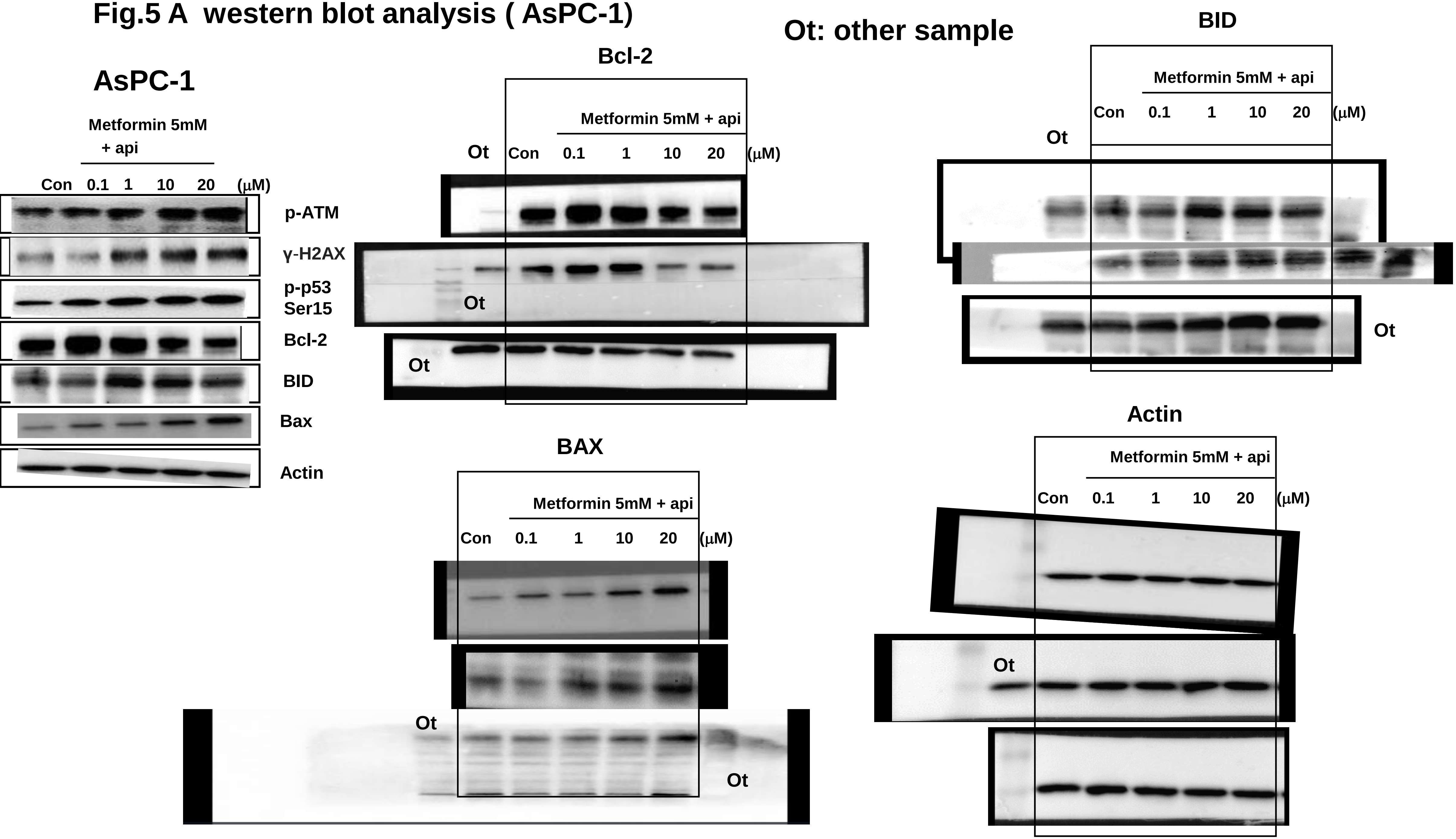

Fig.5 B western blot analysis (HDF)

Ot: other sample

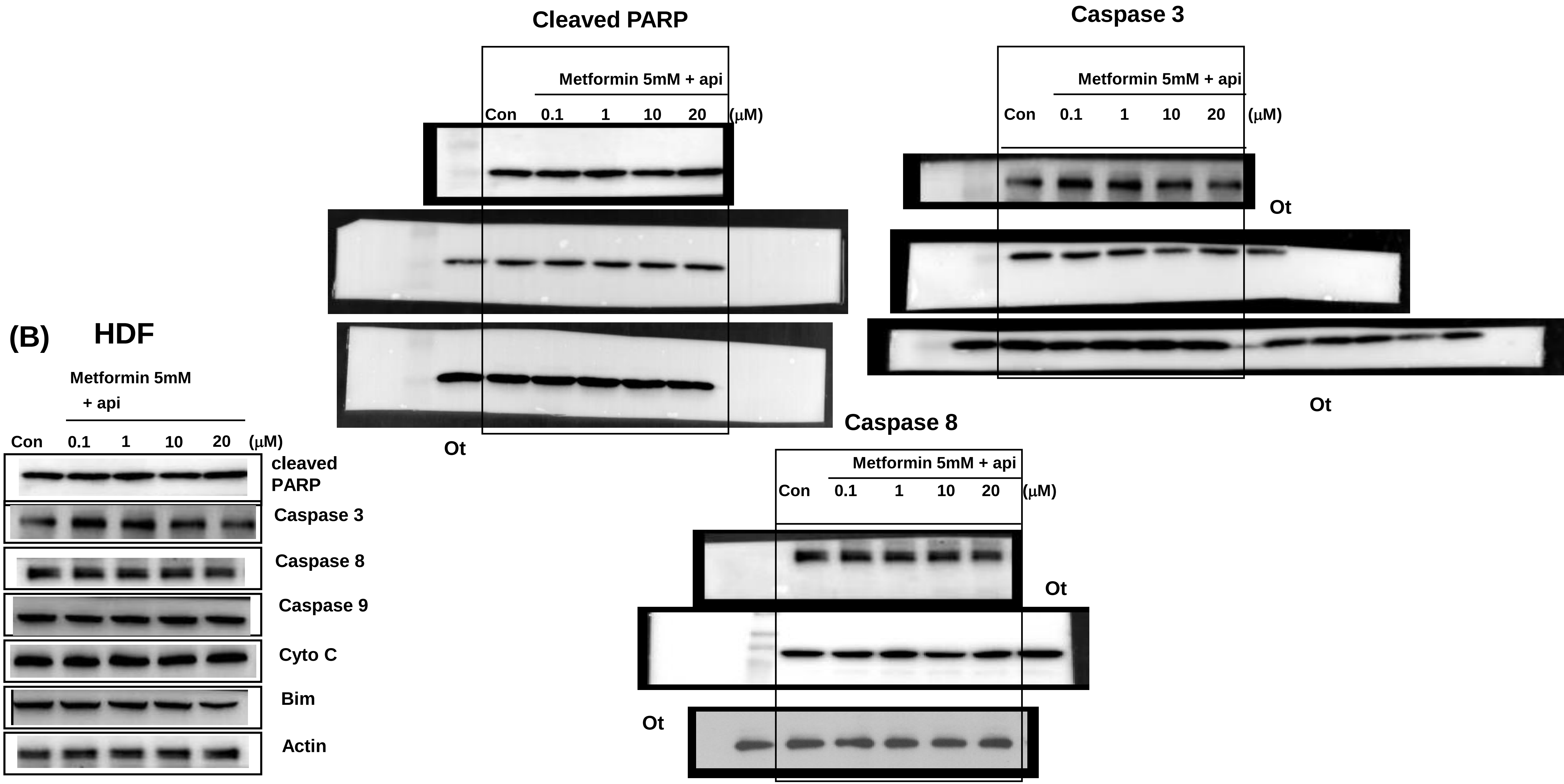

Fig.5 B western blot analysis (HDF)

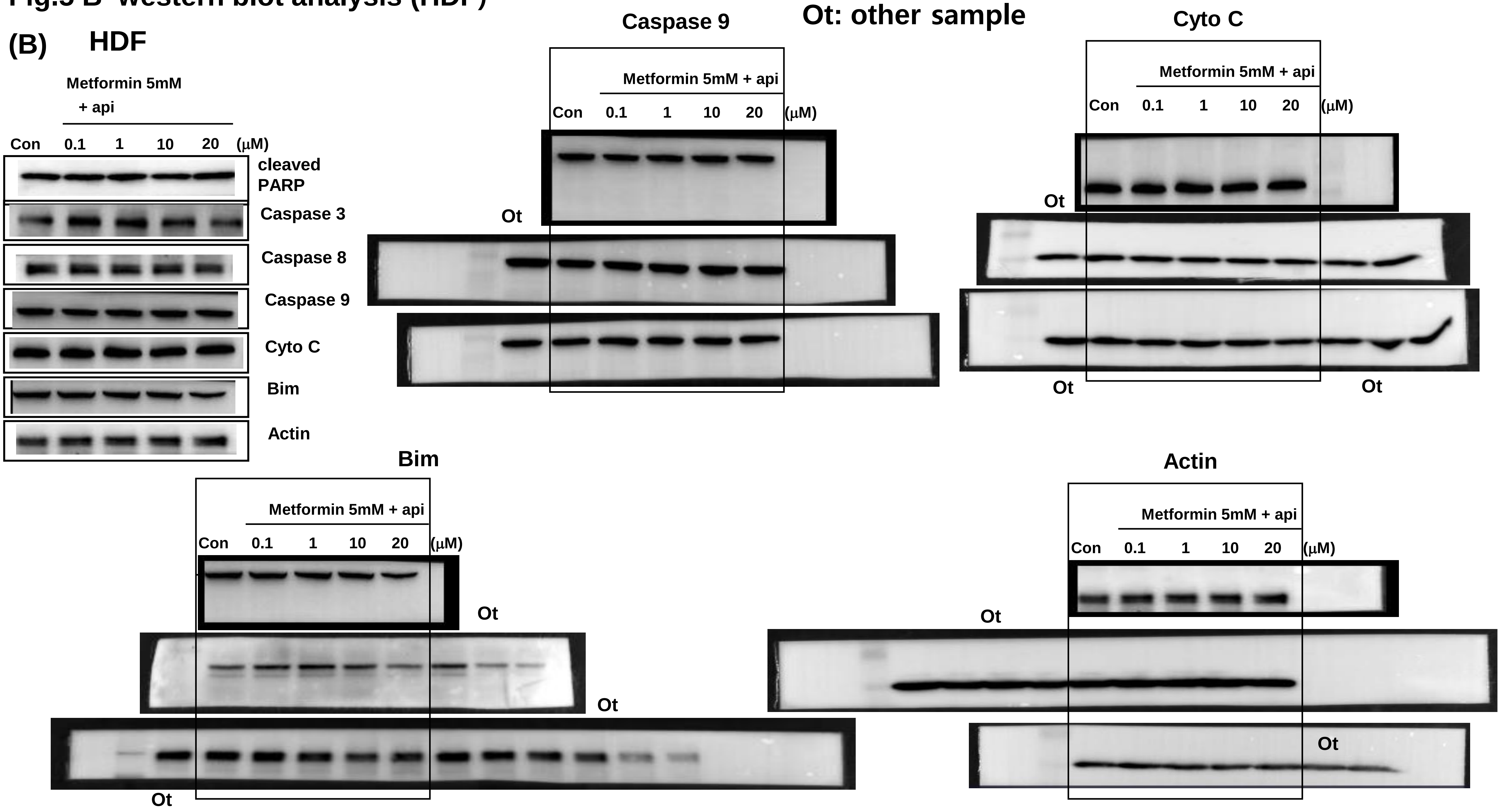

Fig.5 B western blot analysis (AsPC-1)

Ot: other sample

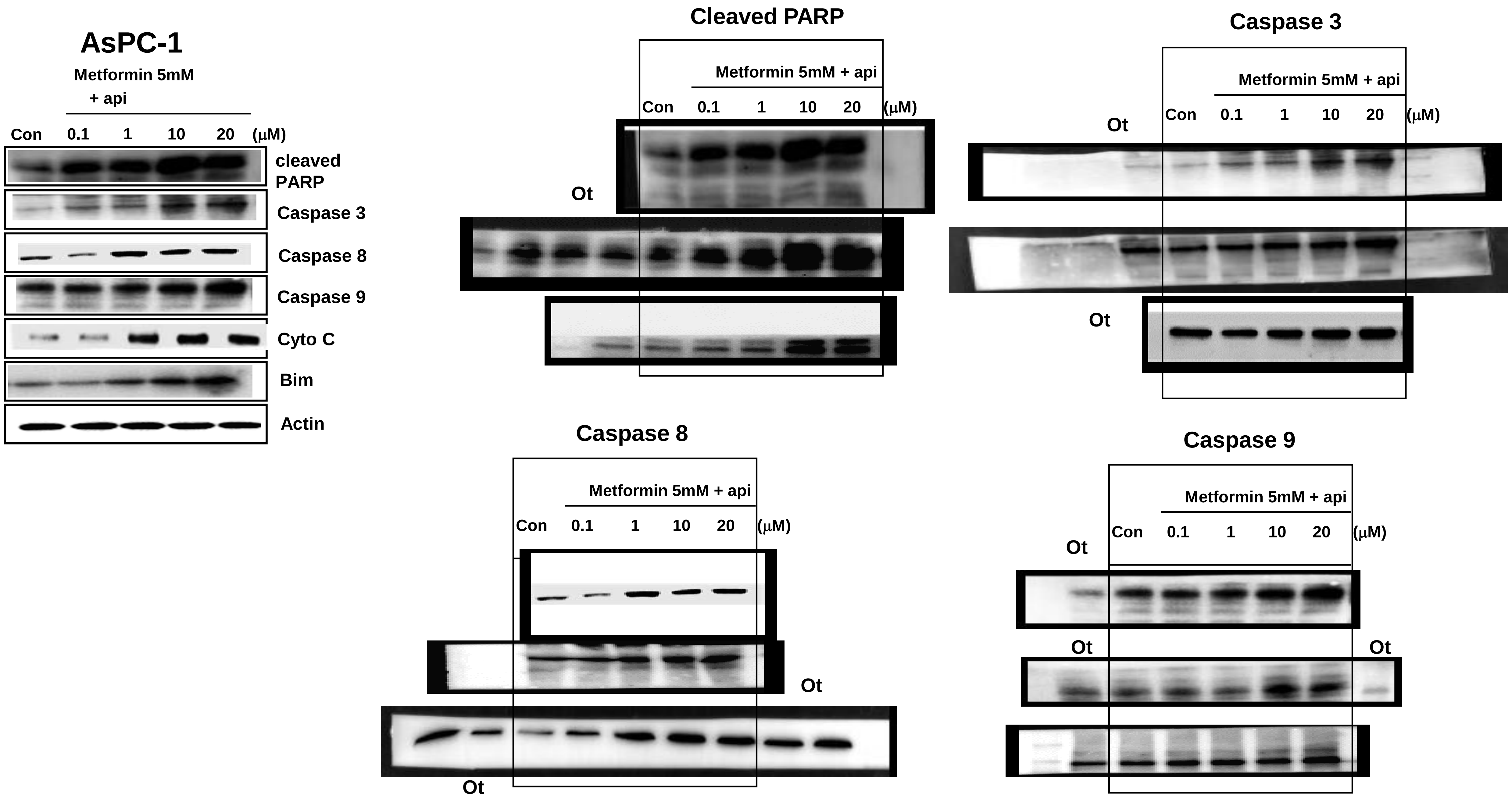

Fig.5 B western blot analysis (AsPC-1)

Ot: other sample

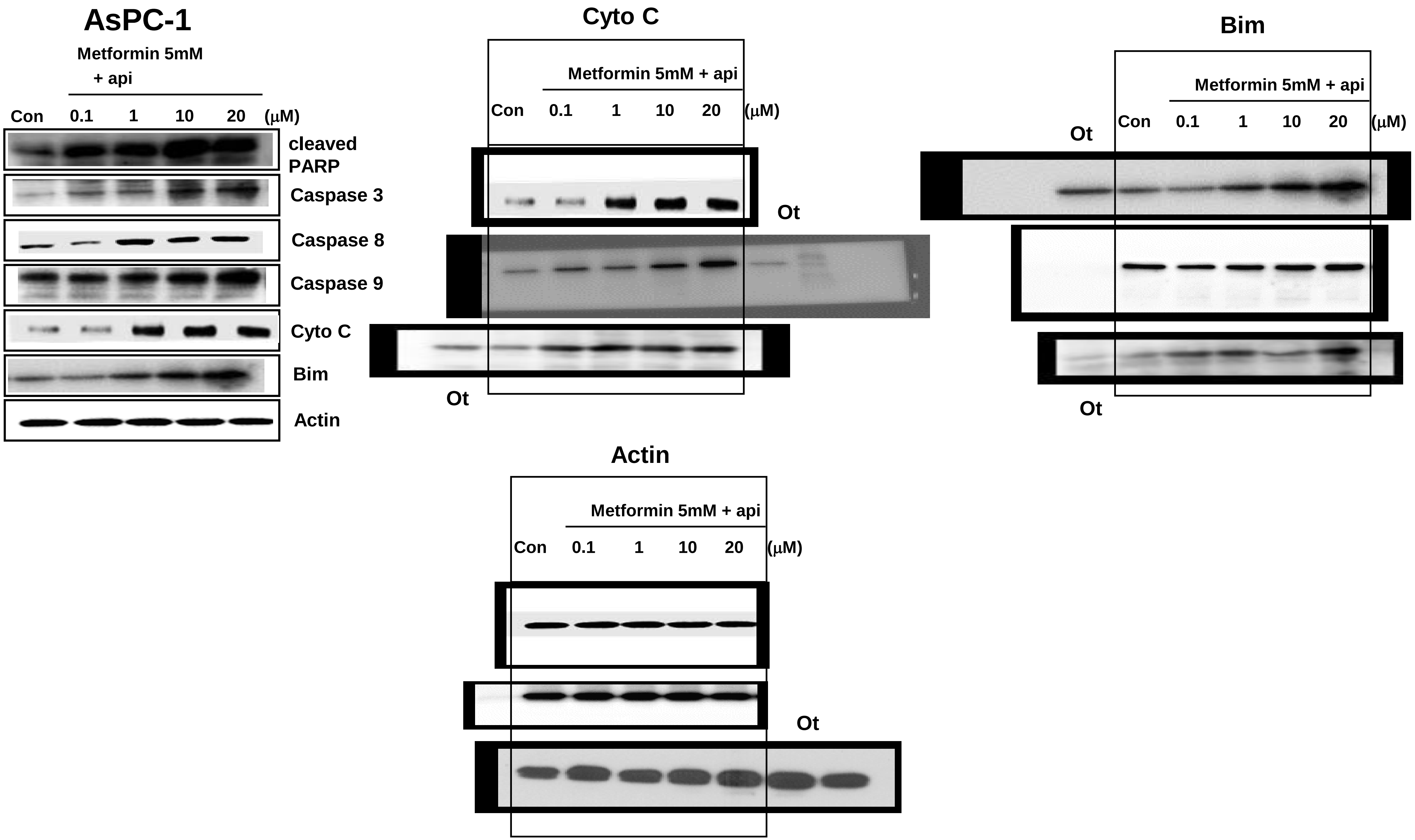

Fig.6 A western blot analysis (HDF)

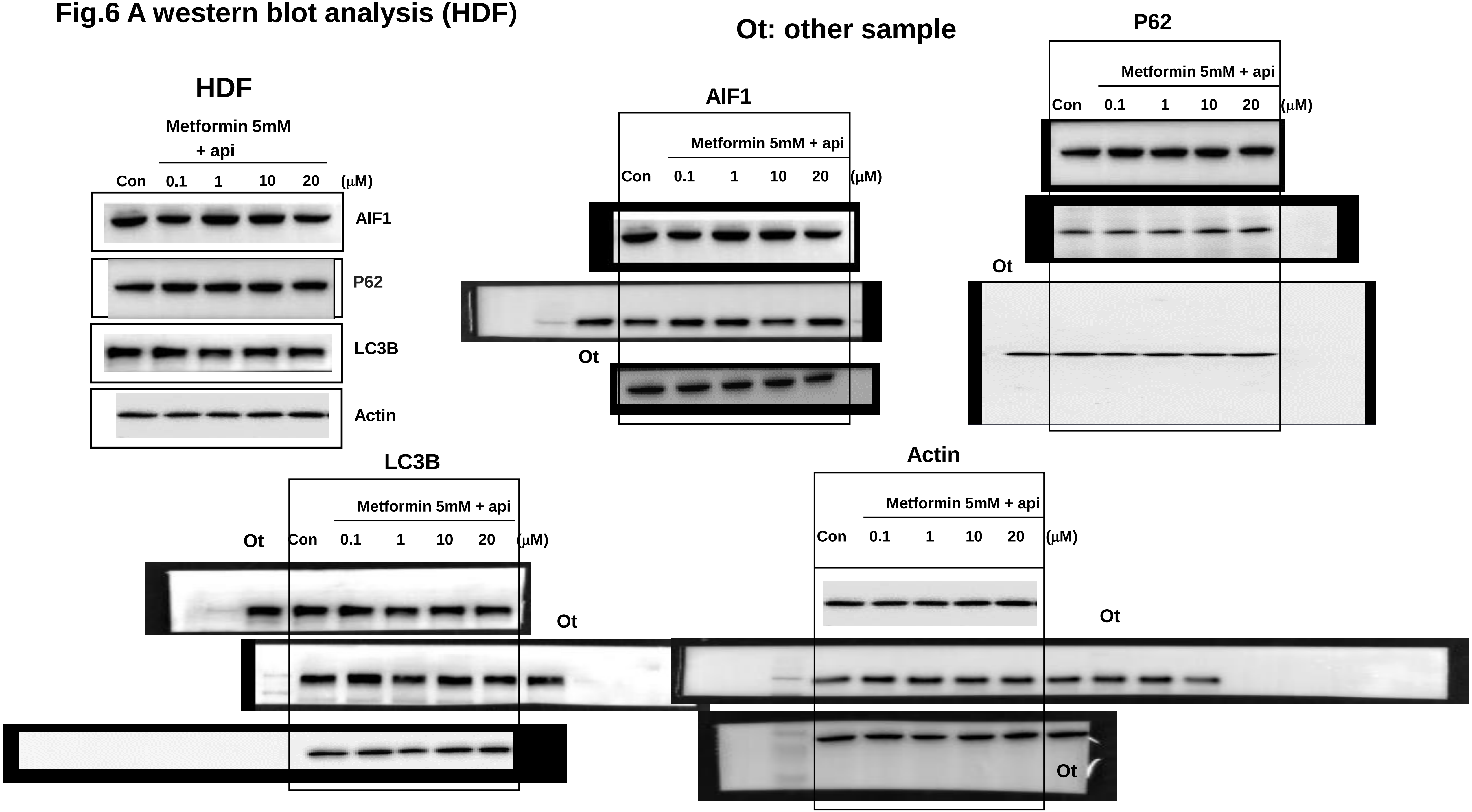

Fig.6 A western blot analysis (AsPC-1)

Ot: other sample

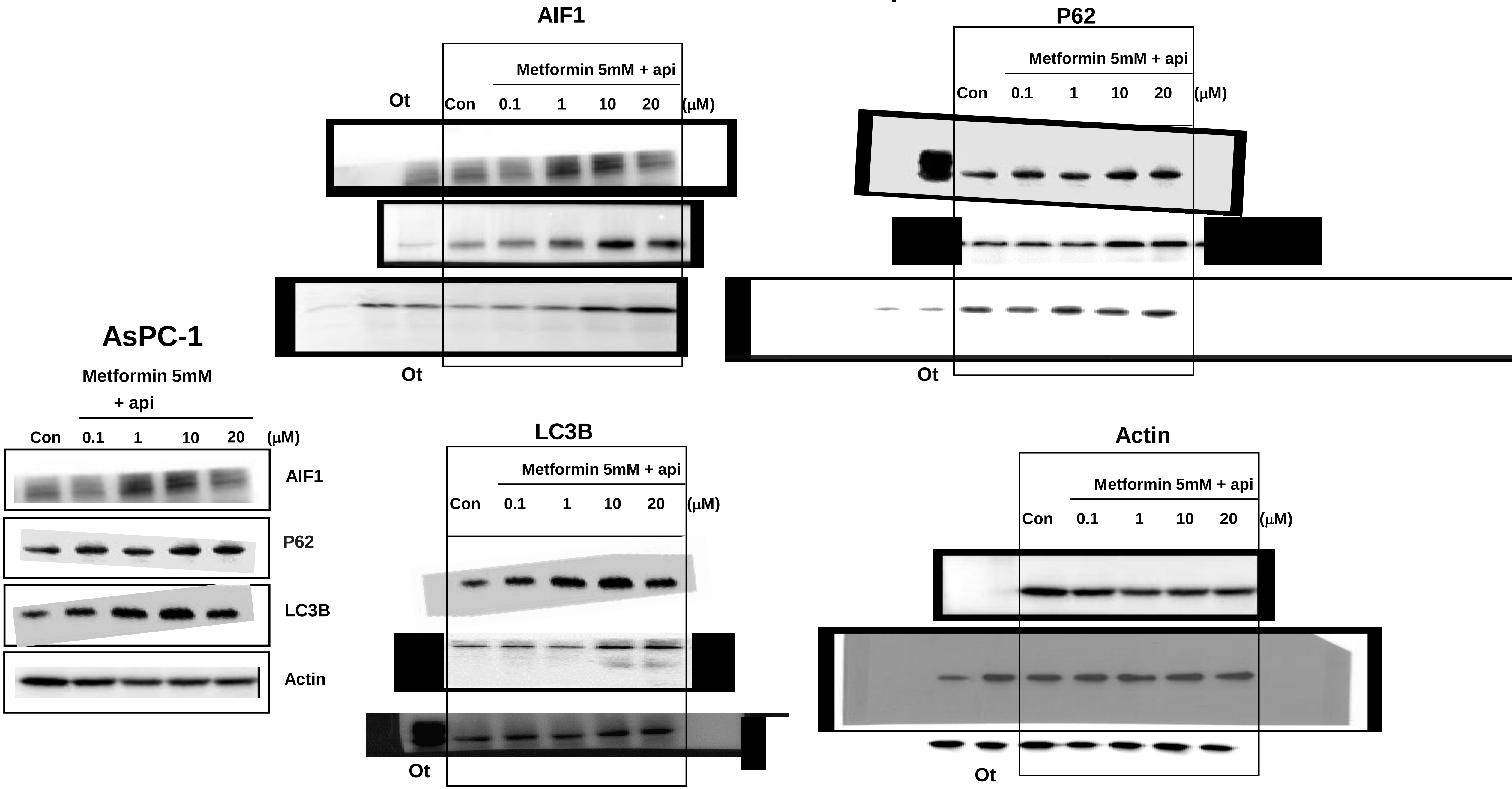

Fig.6 B western blot analysis (HDF)

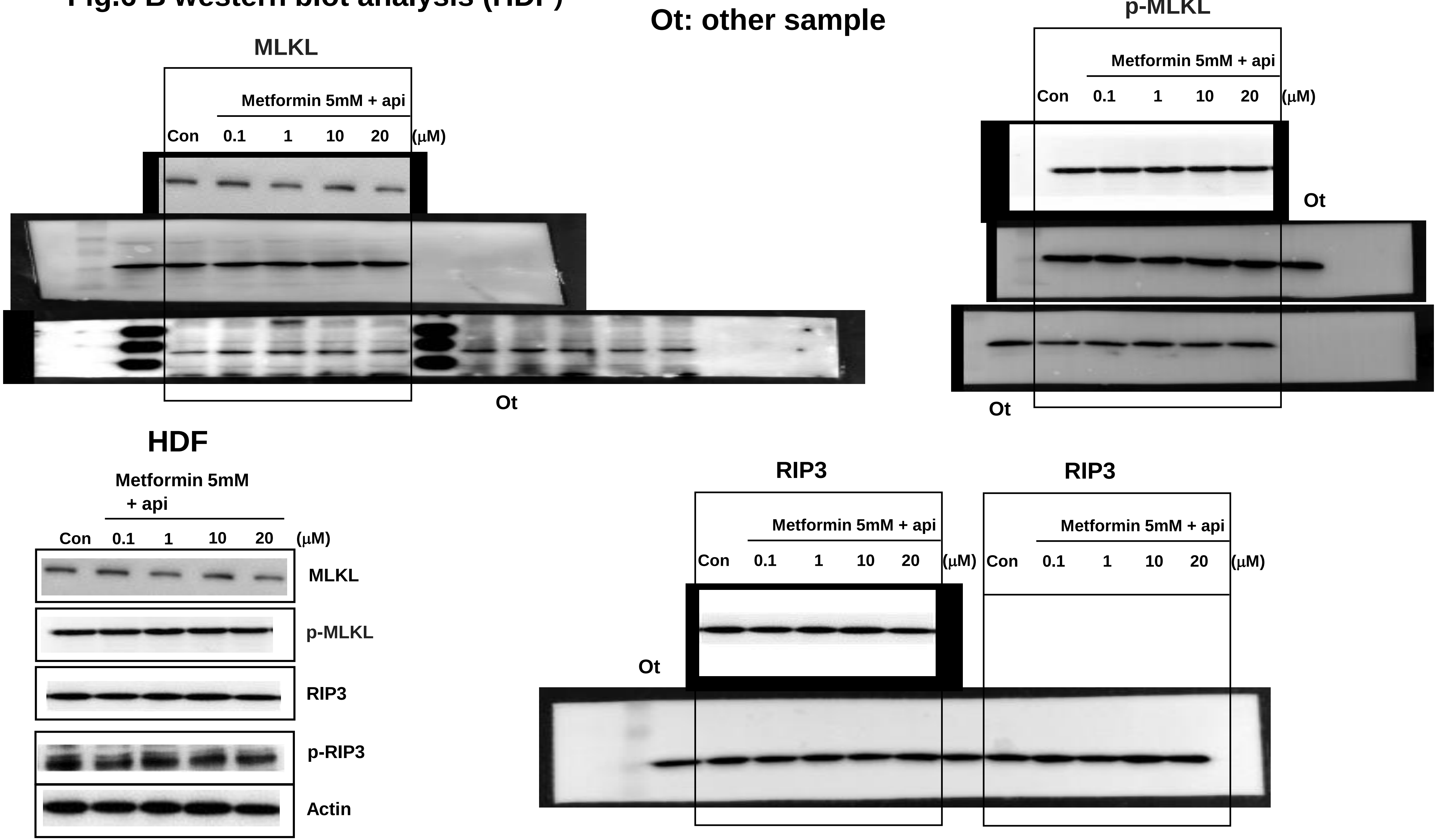

Fig.6 B western blot analysis (HDF)

Ot: other sample

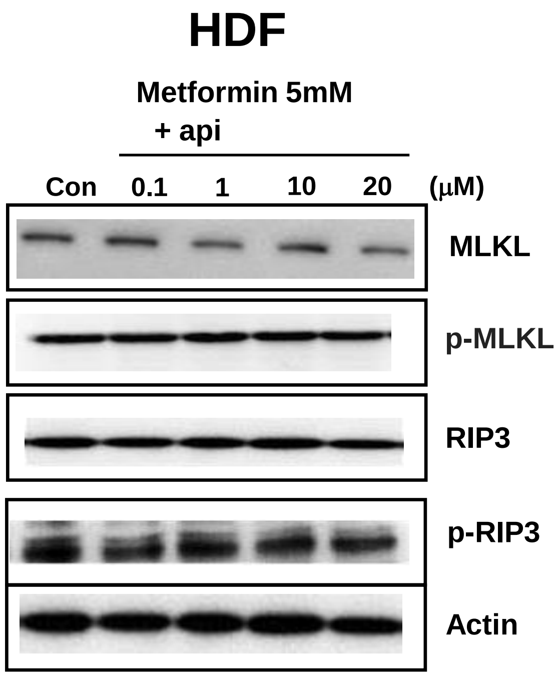

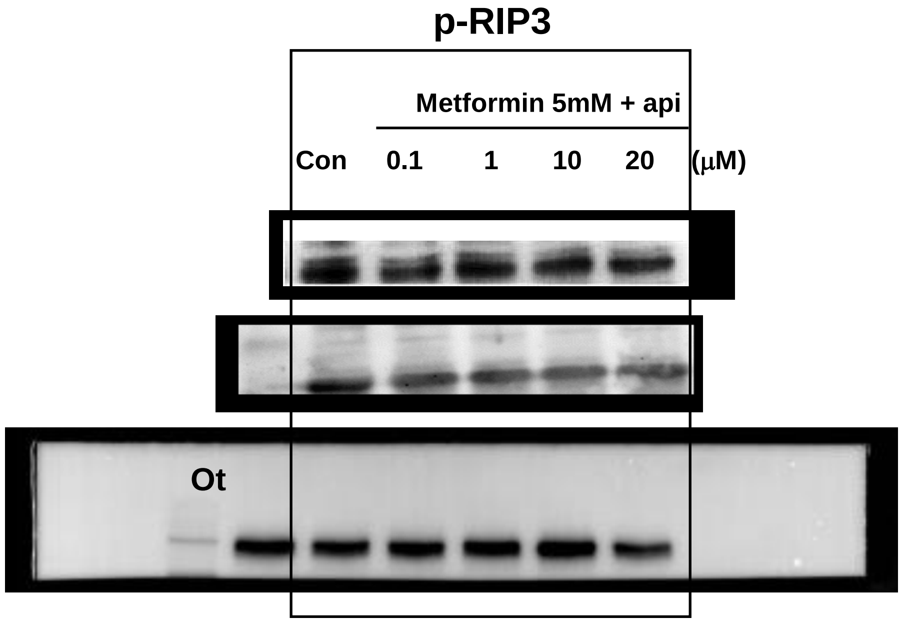

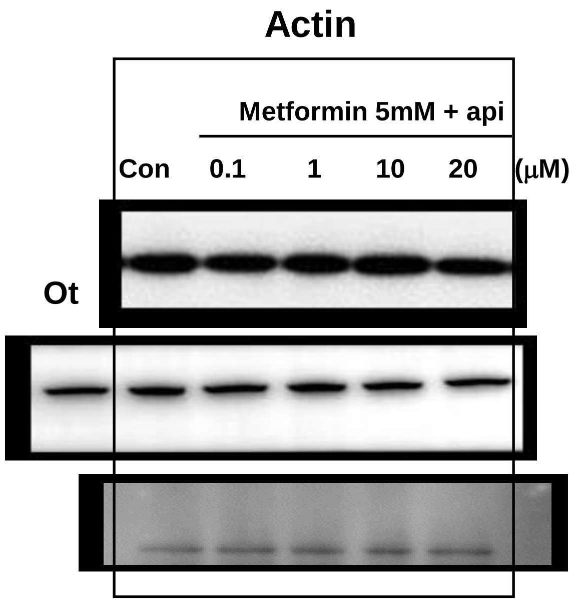

Fig.6 B western blot analysis (AsPC-1)

Ot: other sample

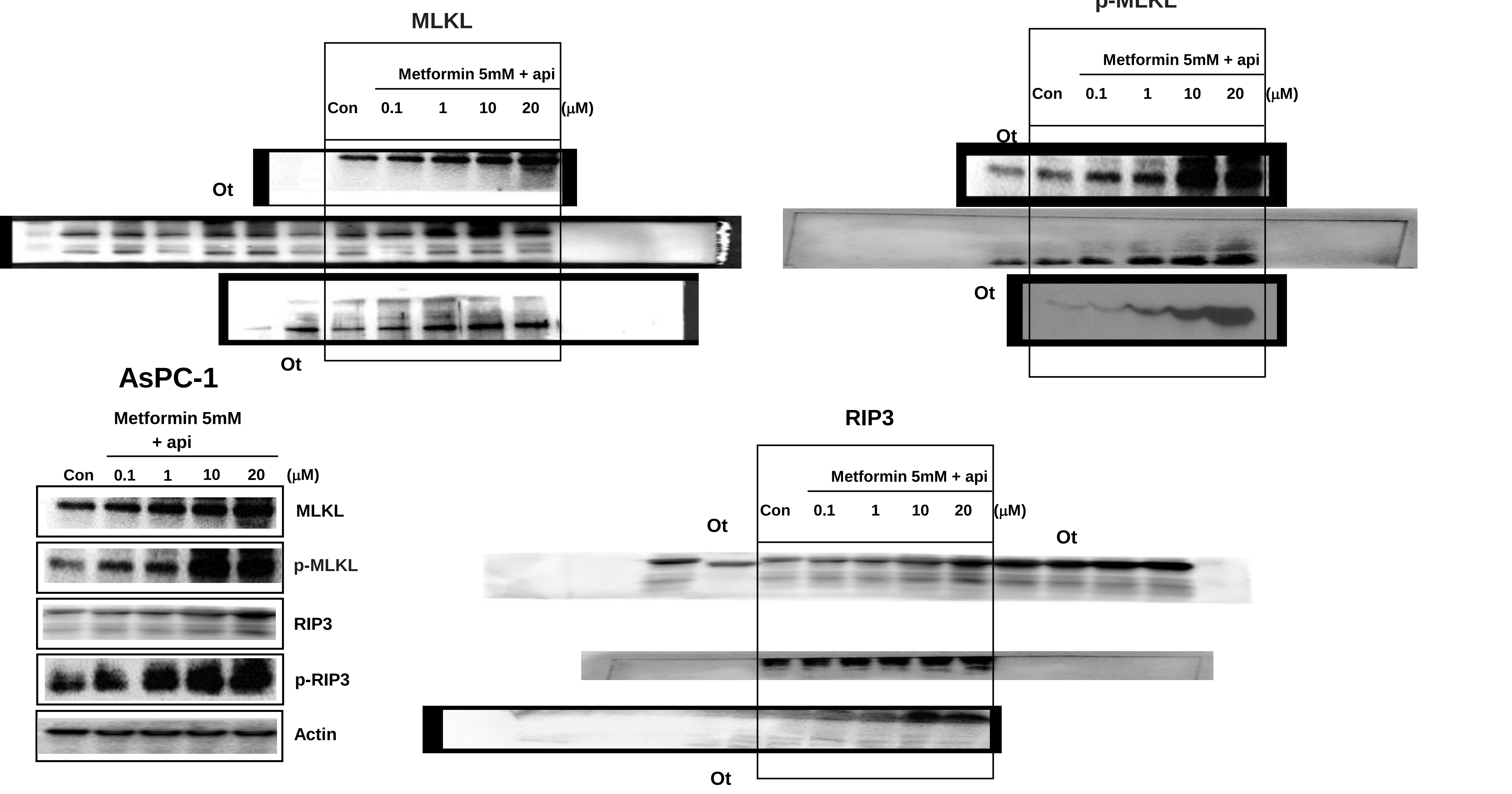

Fig.6 B western blot analysis (AsPC-1)

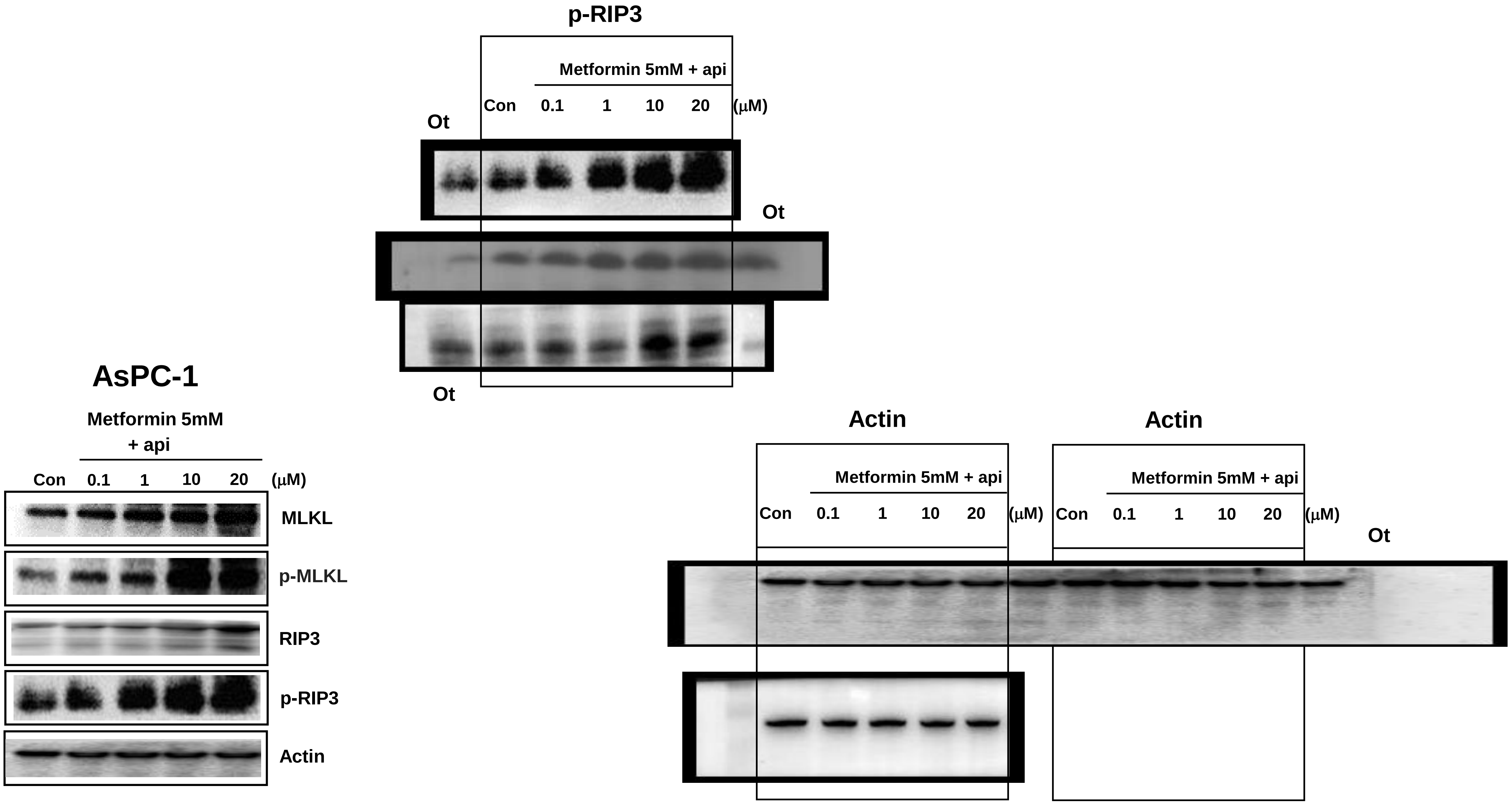

Figure 7A Animal study low dose raw data (tumor volume)

GROUP	NO.	DAY 0	DAY 7	DAY 14	DAY 21	DAY 28
Control	1	80	90	405	850	1258
	2	68	150	430	950	1200
	3	70	190	195	248	750
	4	69	145	150	290	658
	5	90	170	250	756	1400
	6	93	176	350	570	880
	7	85	125	175	425	950
	8	95	150	180	670	750
AVERAGE		81.25	149.50	266.88	594.88	980.75
SE		3.94	11.12	39.57	91.18	96.59
Metformin 75 mg/kg	1	95	130	350	750	1450
	2	85	80	240	350	780
	3	84	130	50	75	126
	4	70	80	48	280	370
	5	85	120	420	680	1250
	6	75	90	120	400	980
	7	78	99	240	680	850
AVERAGE		81.71	104.14	209.71	459.29	829.43
SE		3.08	8.42	54.65	94.77	175.49
Apigenin 5 mg/kg	1	85	95	150	650	960
	2	82	65	180	530	840
	3	80	120	250	450	760
	4	91	150	196	340	890
	5	83	96	138	490	780
	6	75	120	240	570	975
	7	73	115	225	670	800
AVERAGE		81.29	108.71	197.00	528.57	857.86
SE		2.30	10.07	16.49	43.56	32.55
Met+Api (75+5 mg/kg)	1	80	62.5	85	120	170
	2	90	87	110	160	200
	3	82	76	130	140	310
	4	78	102	180	240	300
	5	83	90	175	268	520
	6	80	85	115	380	850
	7	75	95	200	420	920
AVERAGE		81.14	85.36	142.14	246.86	467.14
SE		1.78	4.89	16.21	44.52	116.16

Figure 7B Animal study low dose raw data (tumor weight)

	Control	Metformin 75 mg/kg	Apigenin 5 mg/kg	Met 75 + Api 5 mg/kg
1	450	420	432	305
2	412	445	298	270
3	421	382	420	273
4	320	371	430	281
5	505	320	330	332
6	480	280	342	255
7	432	452	428	250
8	412			
AVERAGE	429.00	381.43	382.86	280.86
SE	19.53	24.24	21.67	10.91

Figure 7D Animal study high dose raw data (tumor volume)

GROUP	NO.	DAY 1	DAY 8	DAY 15	DAY 22	DAY 28
Control	1	80	75	600	1267.5	1436.5
	2	67.5	126	445.5	650	1568
	3	67.5	75	171.5	220.5	864
	4	67.5	108	108	288	405
	5	92.5	144	320	786.5	1912.5
	6	92.5	126	364.5	352	700
	7	80	126	196	320	786.5
	8	92.5	144	108	288	600
AVERAGE		80.00	115.50	289.19	521.56	1034.06
SE		4.09	9.72	62.08	127.63	189.20
Metformin 125 mg/kg	1	113	144	405	665.5	1912.5
	2	80	75	171.5	144	320
	3	80	126	32	62.5	126
	4	67.5	75	18	0	0
	5	80	108	600	786.5	1152
	6	67.5	87.5	32	0	32
	7	80	75	32	0	0
	8	80	87.5	13.5	0	0
AVERAGE		81.00	97.25	163.00	207.31	442.81
SE		4.99	9.26	78.43	115.13	251.26
Apigenin 40 mg/kg	1	80	48	0	32	62.5
	2	80	48	18	0	0
	3	82.5	56	22.5	0	4
	4	92.5	62.5	22.5	0	0
	5	80	48	22.5	320	550
	6	80	87.5	75	245	550
	7	80	87.5	112.5	0	0
	8	80	48	18	0	0
AVERAGE		81.88	60.69	36.38	74.63	145.81
SE		1.55	6.13	13.27	46.08	88.52
Met+Api (125+40 mg/kg)	1	80	62.5	0	13.5	100
	2	92.5	32	0	0	0
	3	80	32	0	0	0
	4	80	40	0	0	4
	5	80	75	0	40	196
	6	80	13.5	6	4	10
	7	67.5	62.5	18	0	0
	8	80	18	0	0	0
AVERAGE		80.00	41.94	3.00	7.19	38.75
SE		2.36	7.93	2.27	4.97	25.54

Figure 7E Animal study high dose raw data (tumor weight)

	Control	Metformin 125 mg/kg	Apigenin 40 mg/kg	Met 125 + Api 40 mg/kg
1	712	799	78	98
2	480	476	0	0
3	363	232	7	11
4	315	0	0	0
5	665	576	408	138
6	318	116	402	30
7	355	0	0	0
8	218	0	0	0
AVERAGE	428.25	274.88	111.88	34.63
SE	62.40	108.53	64.65	18.93

Cellular ROS level in L132 cells (normal human epithelial cells , human lung origin) treated with metformin, apigenin and metformin/apigenin.

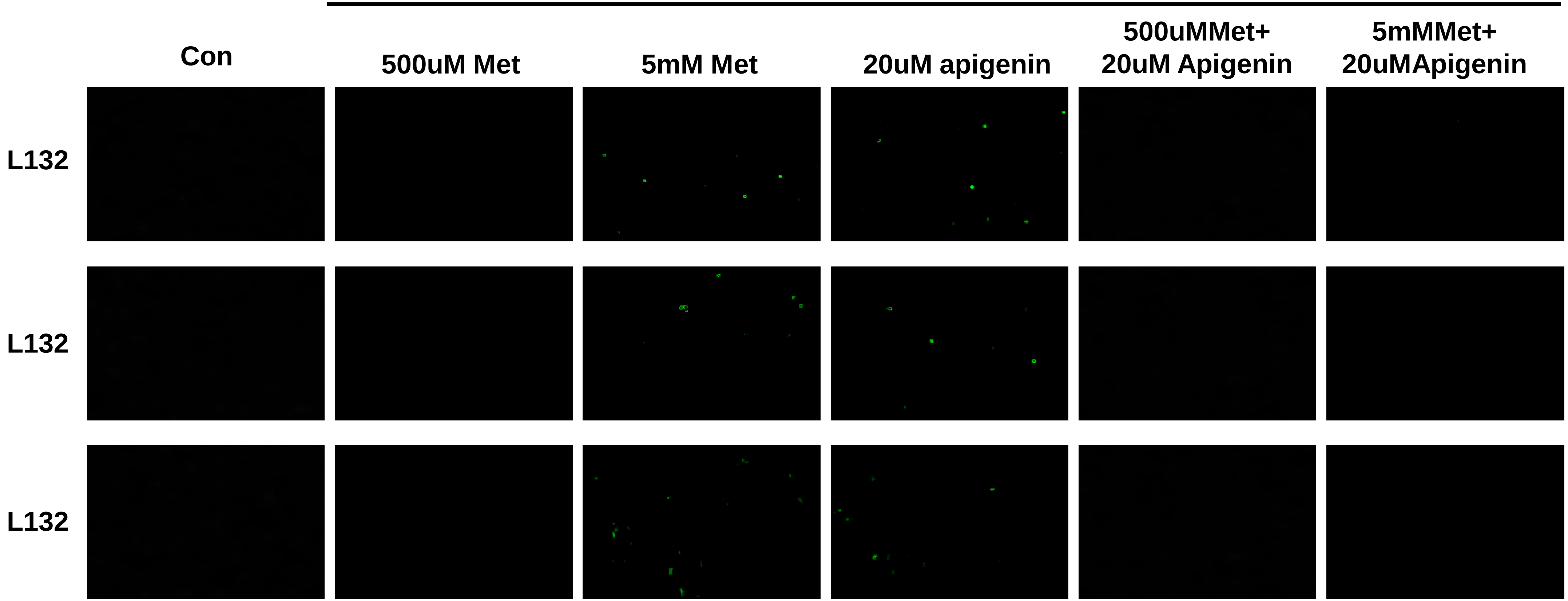

MTT Assay in L132 cells (normal human epithelial cells , human lung origin) treated with metformin, apigenin and metformin/apigenin

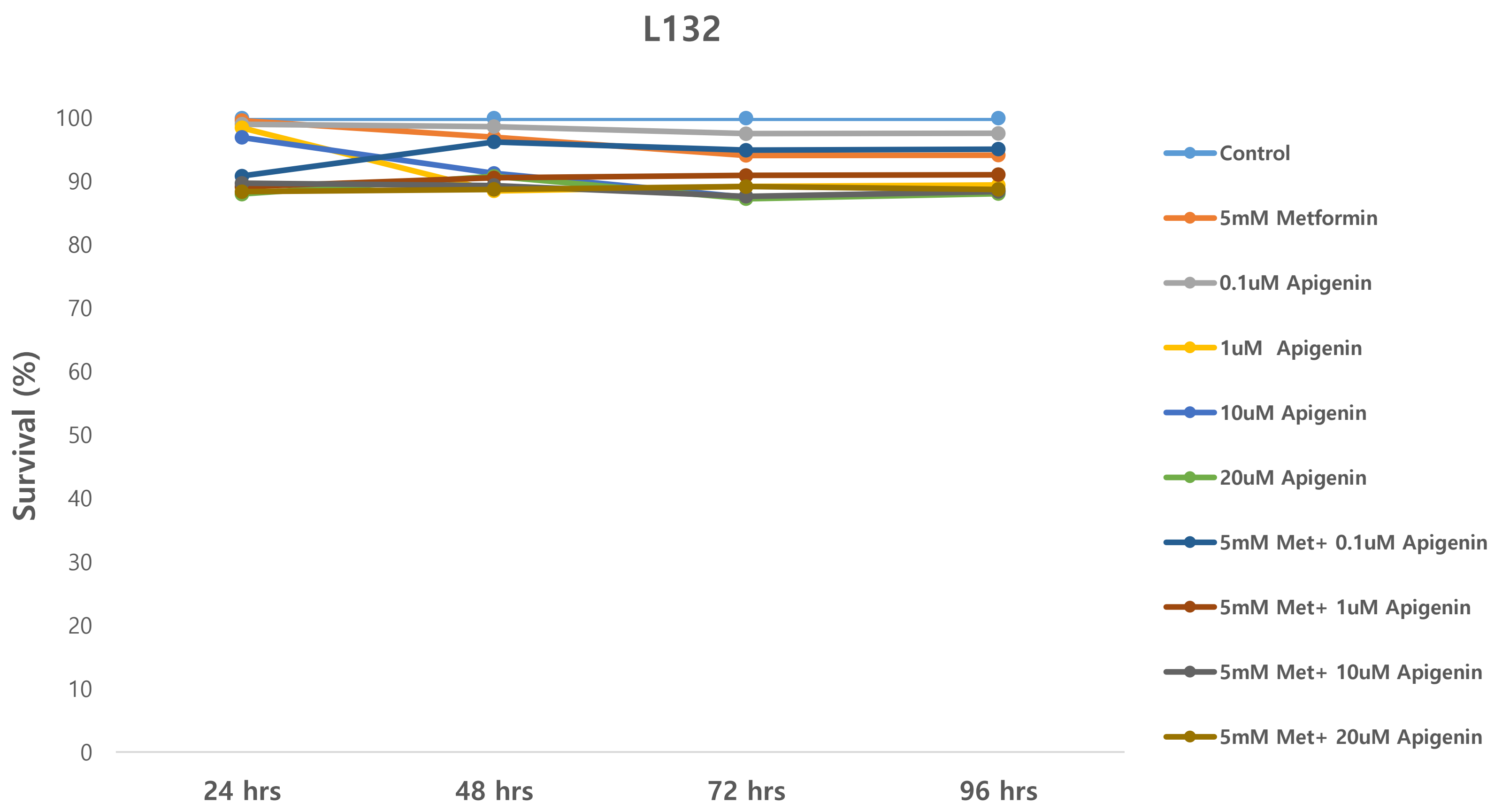

Analysis of mRNA levels(OCT1,2,3, OCTN 1 and 2 and MATE1) by Real time RT-qPCR

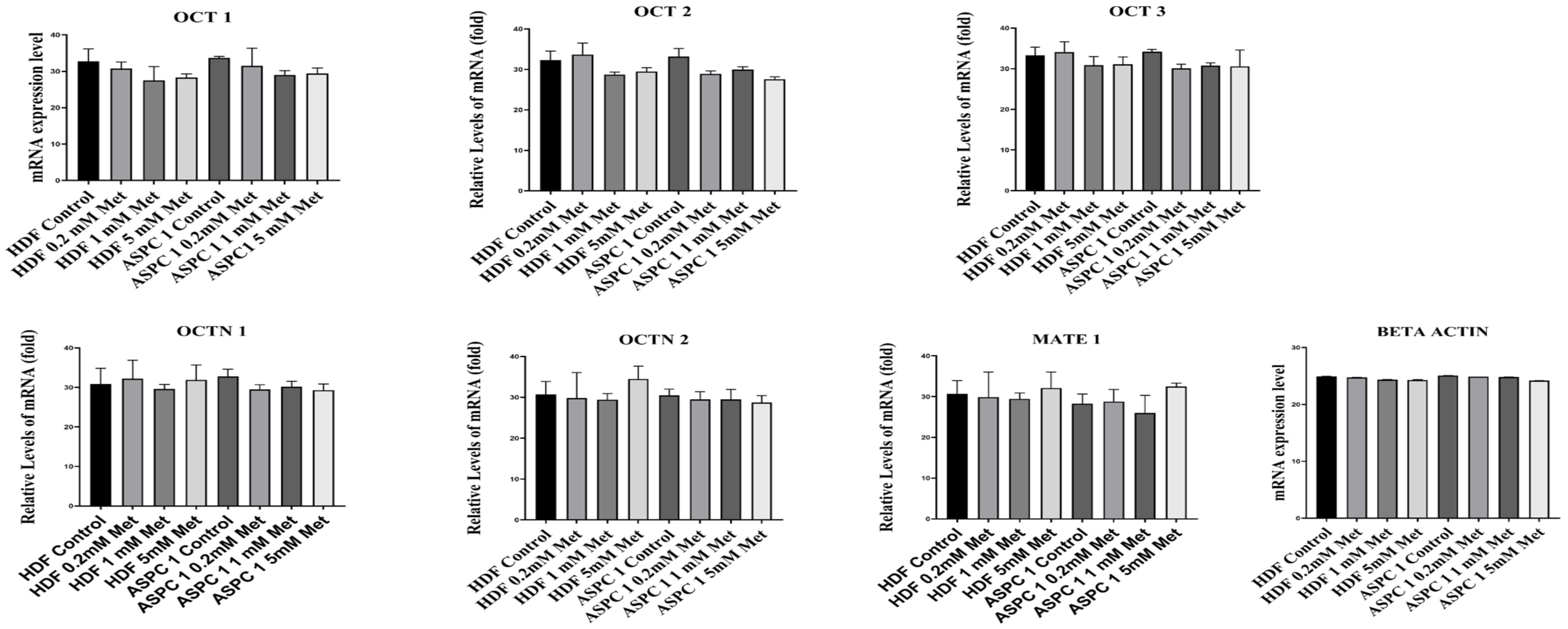

HDF and AsPC-1 cells were treated with different concentrations of metformin (0.2, 1 and 5 mM) for 24 hr and mRNA was isolated. 0.5 µg of mRNA was used for real time RT-qPCR.

Multiple Exposure Images-Western Blot

Fig. 2B western blot analysis (HDF)

Ot: other sample

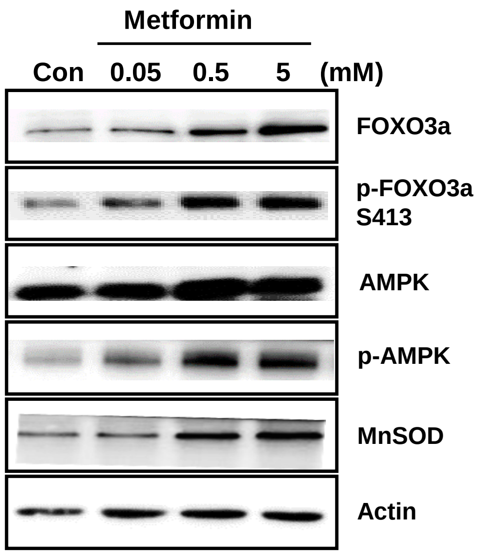

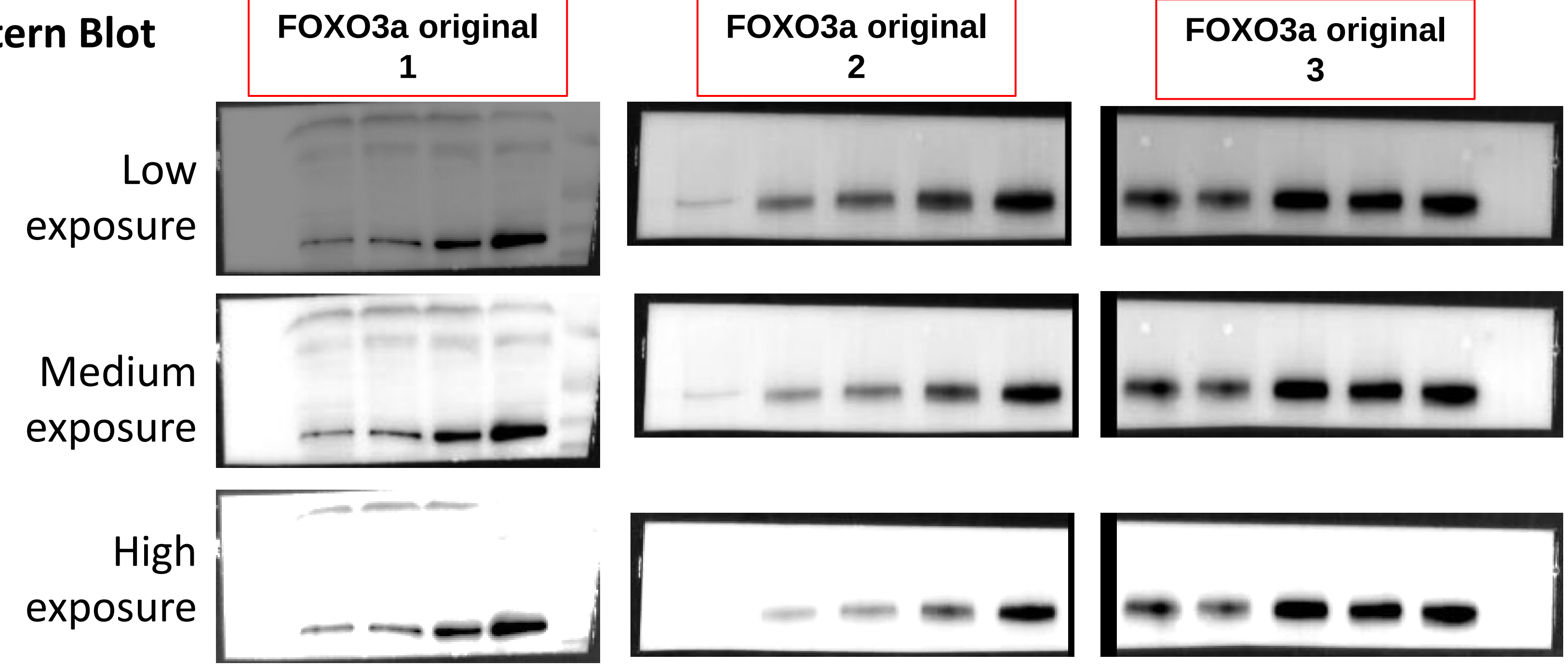

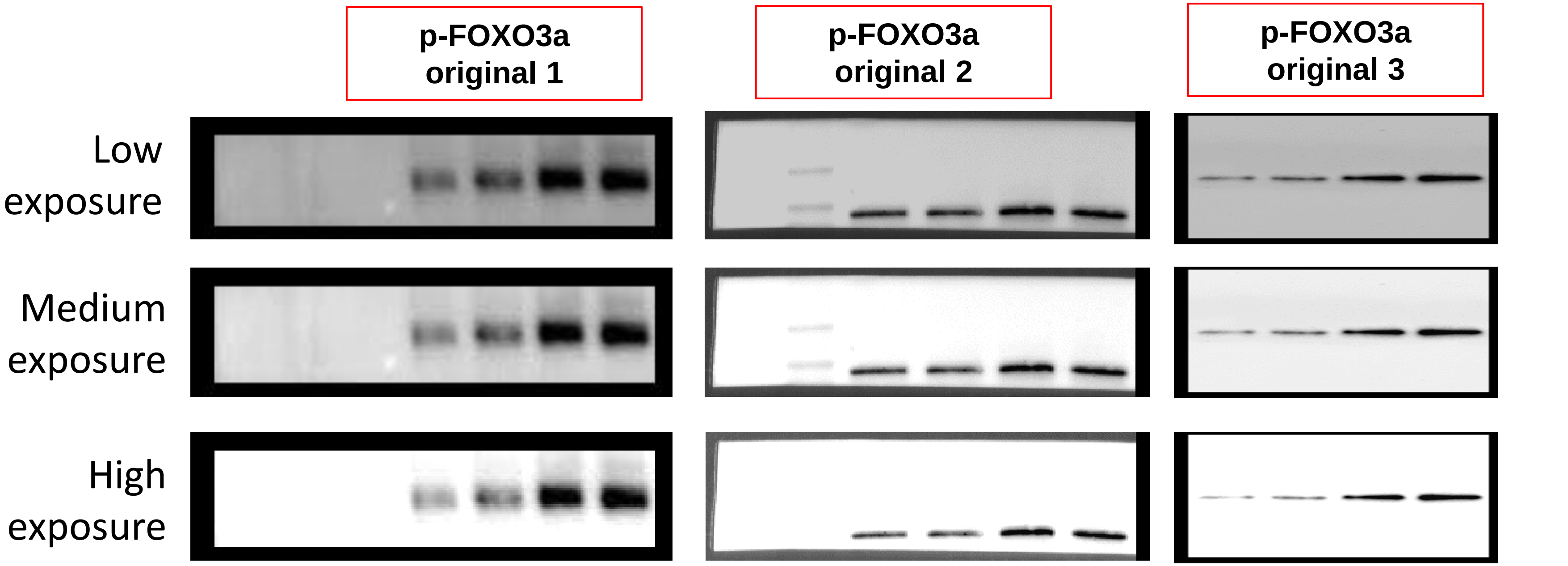

Multiple Exposure Images-Western Blot

Fig. 2B western blot analysis (HDF)

Ot: other sample

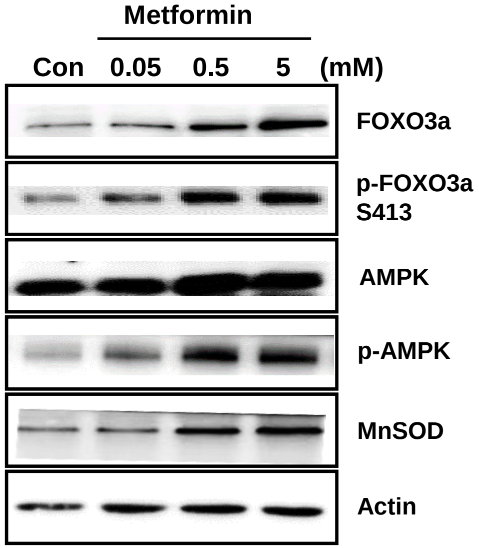

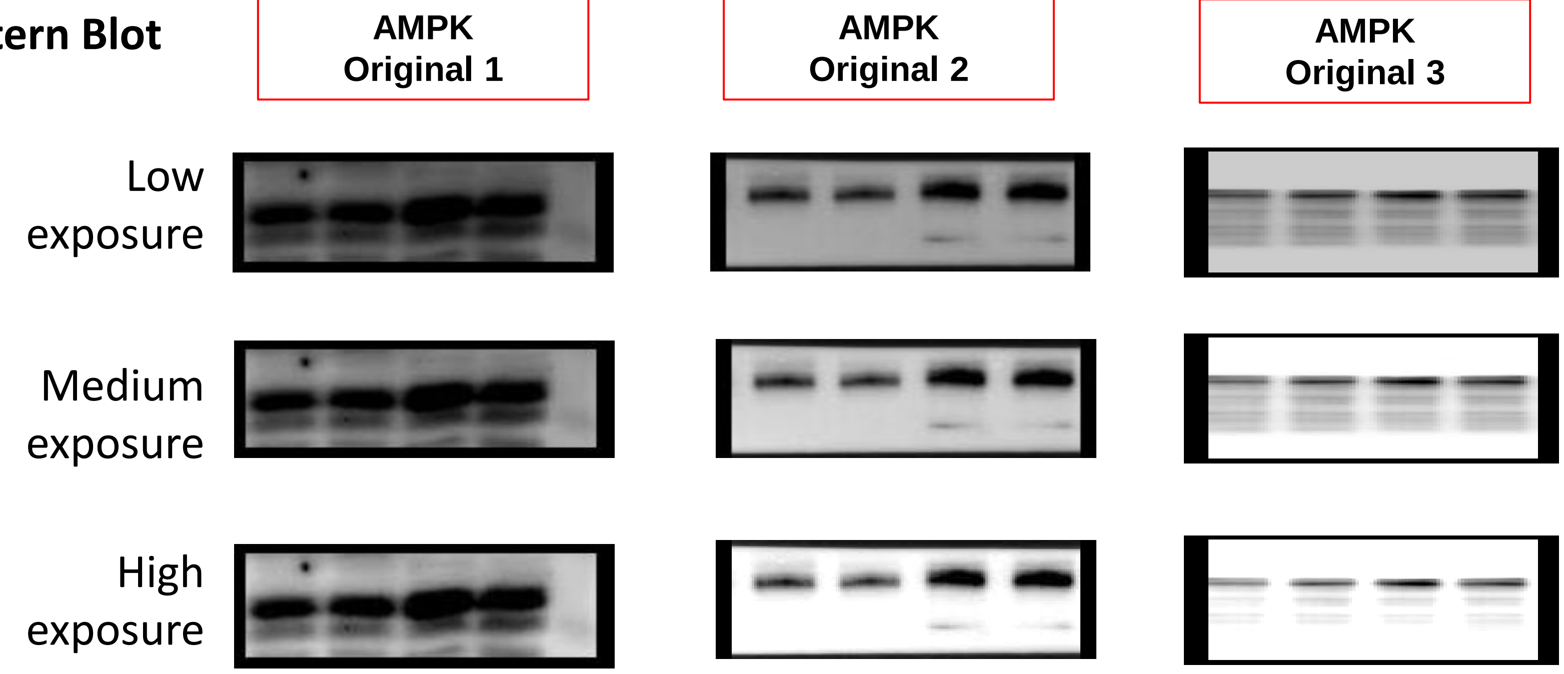

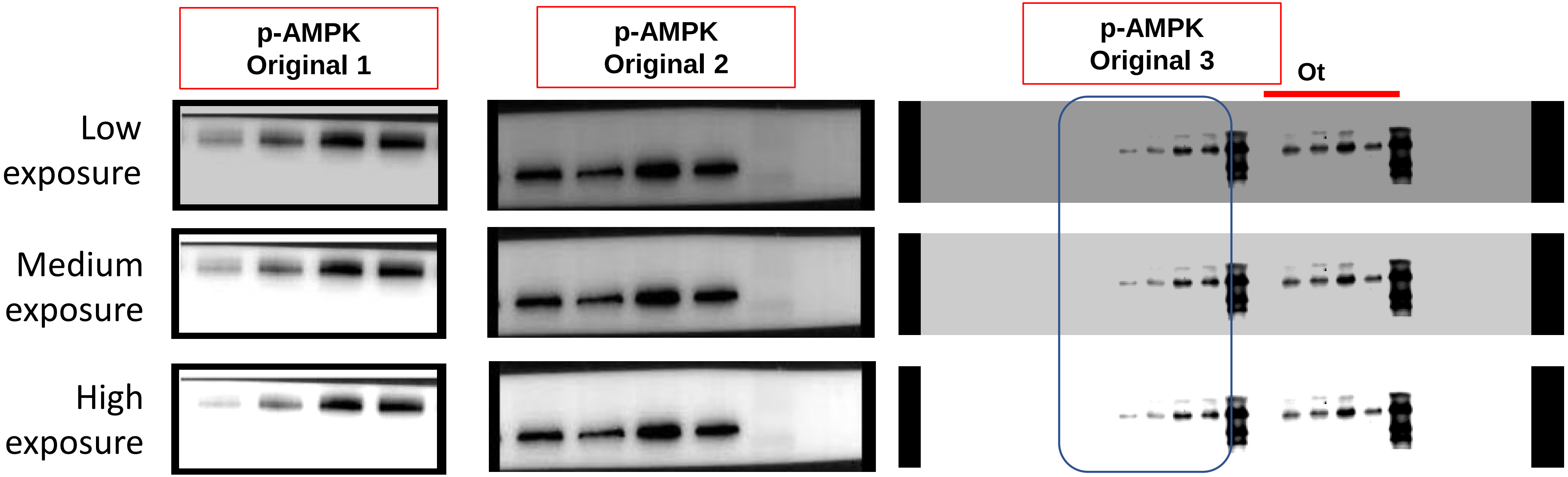

Multiple Exposure Images-Western Blot

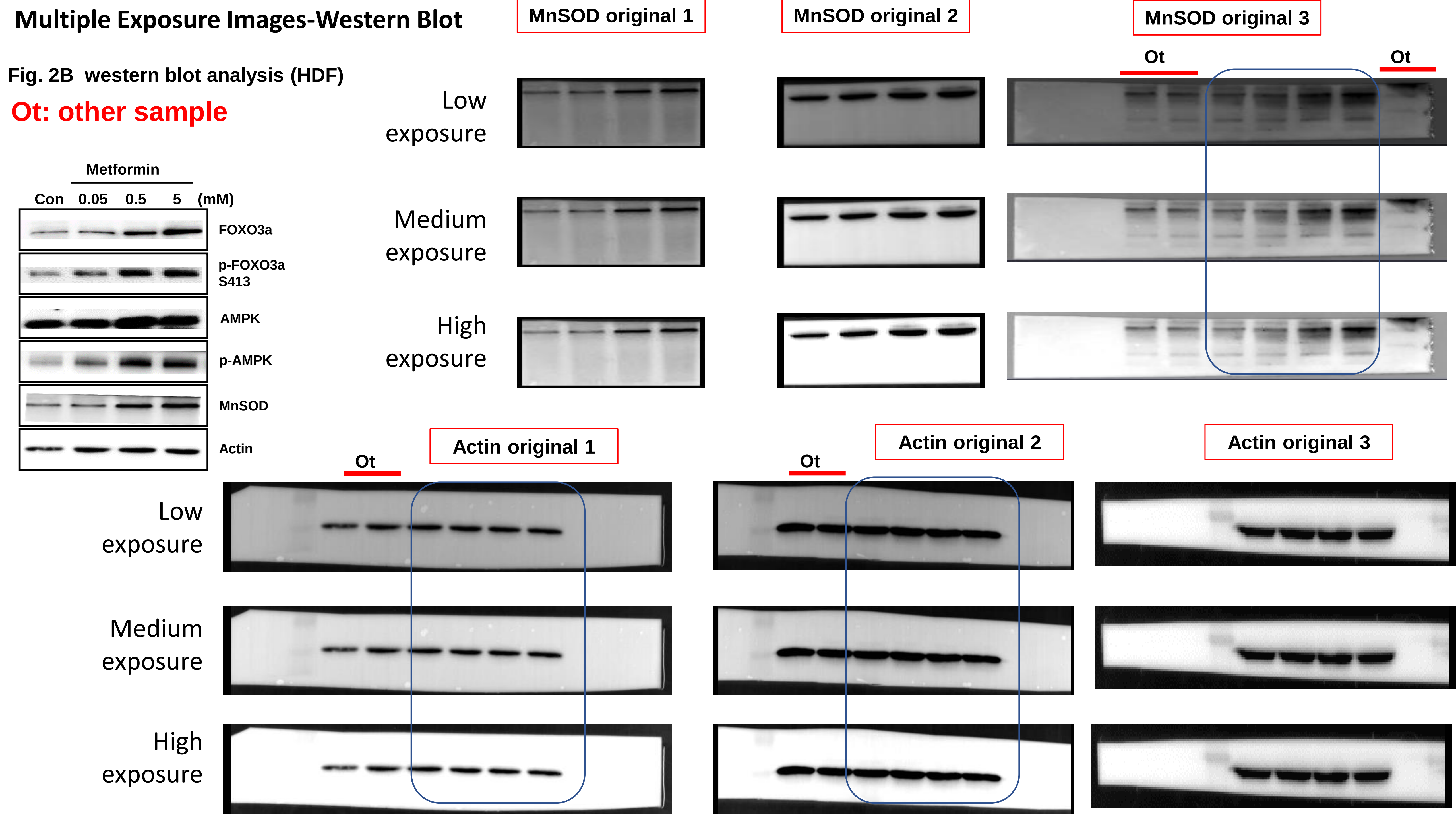

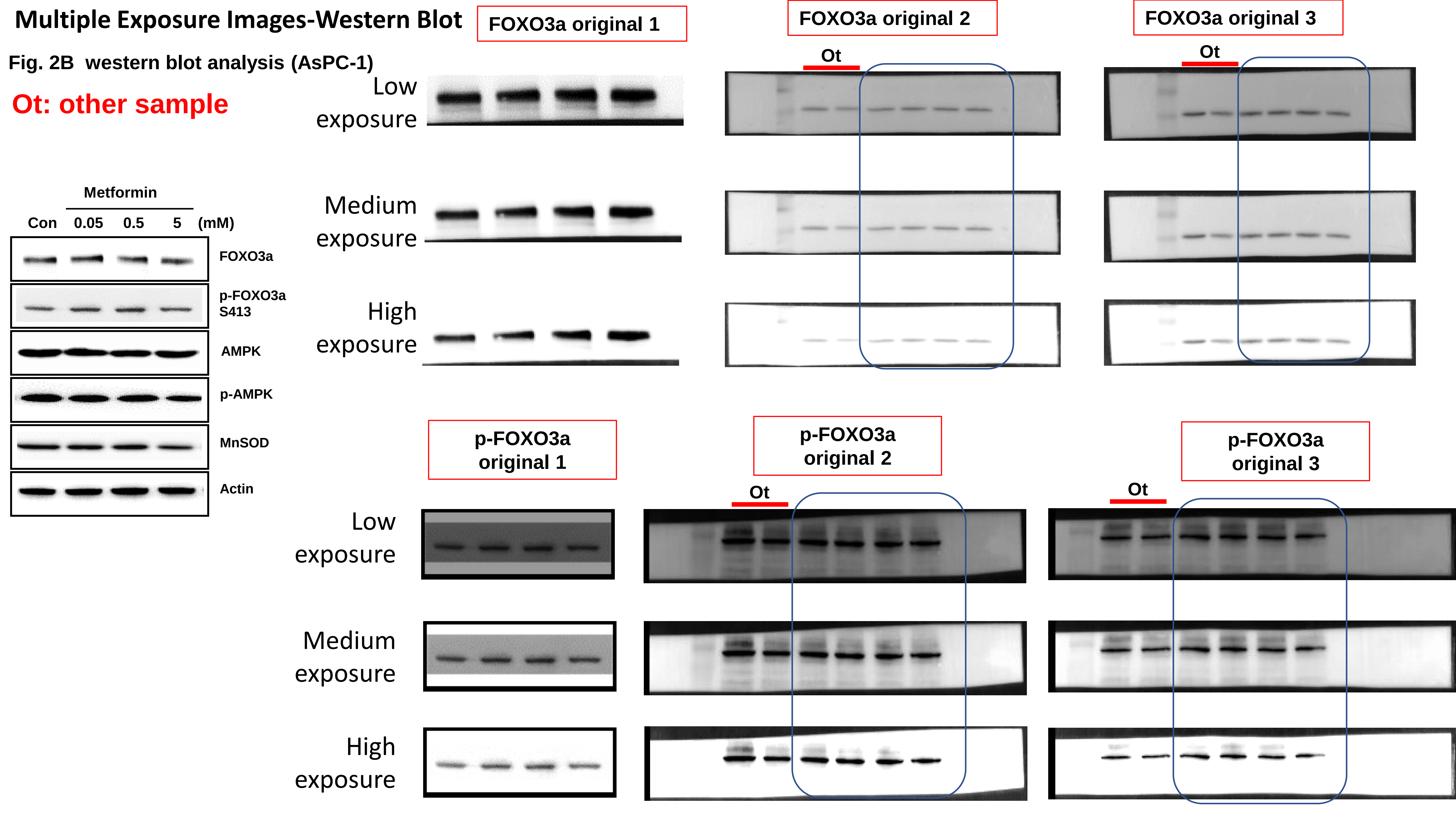
Multiple Exposure Images-Western Blot
Fig. 2B western blot analysis (AsPC-1)
Ot: other sample
Metformin
Con
0.05
0.5
5
(mM)
FOXO3a
p-FOXO3a S413
AMPK
p-AMPK
MnSOD
Actin
FOXO3a original 1
FOXO3a original 2
FOXO3a original 3
Ot
Ot
Low exposure
Medium exposure
High exposure
p-FOXO3a original 1
p-FOXO3a original 2
p-FOXO3a original 3
Ot
Ot
Low exposure
Medium exposure
High exposure

Multiple Exposure Images-Western Blot

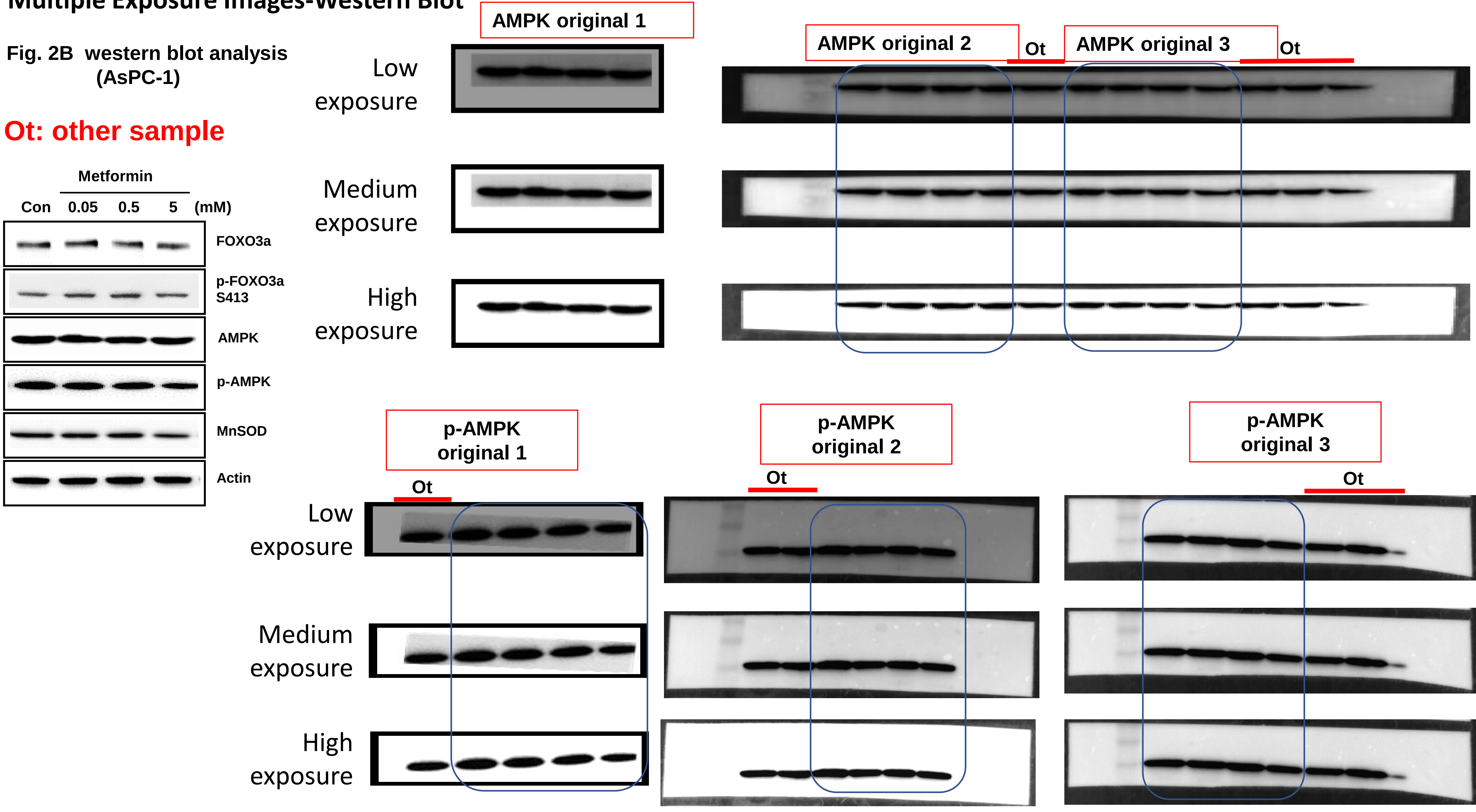

Multiple Exposure Images-Western Blot

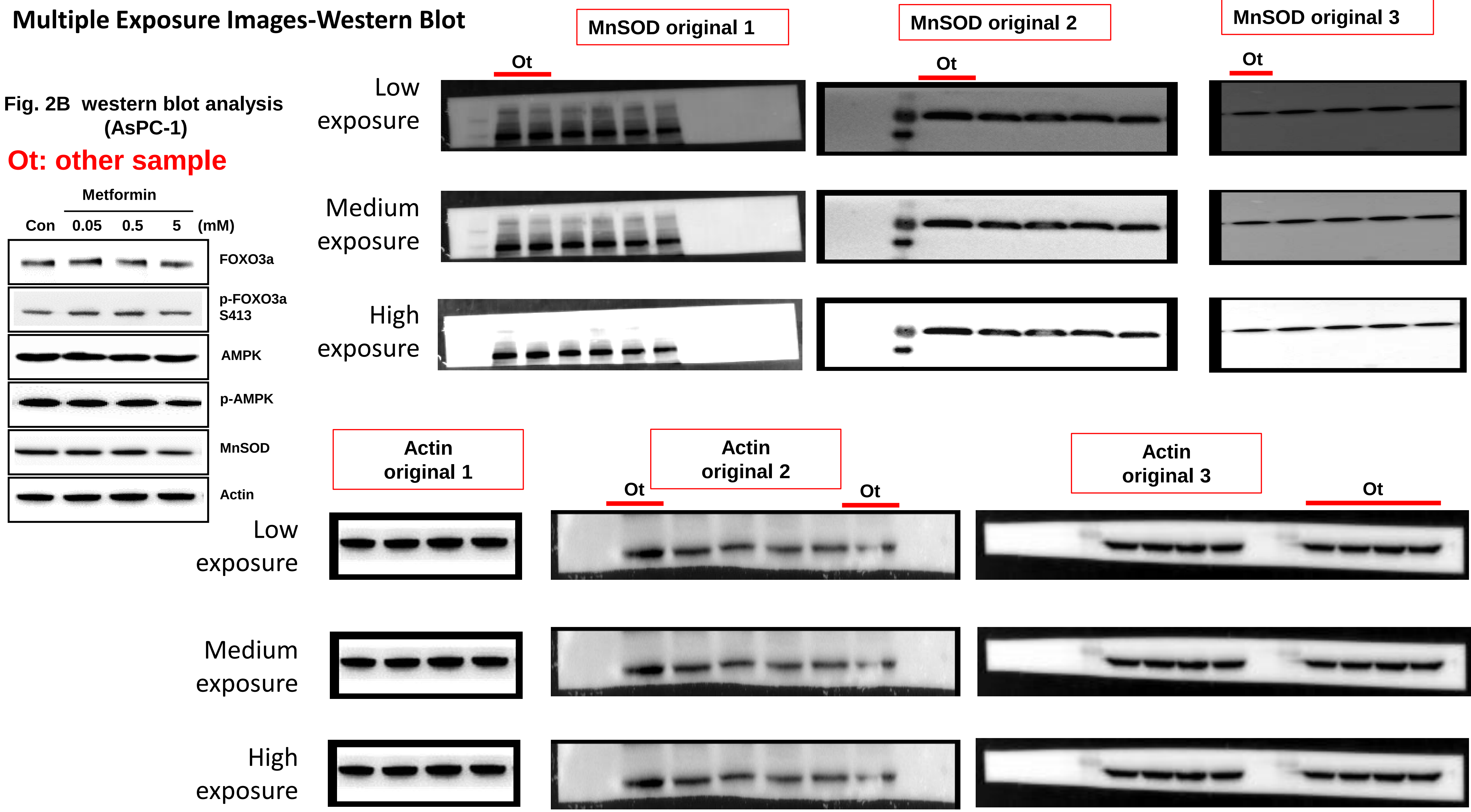

Multiple Exposure Images-Western Blot

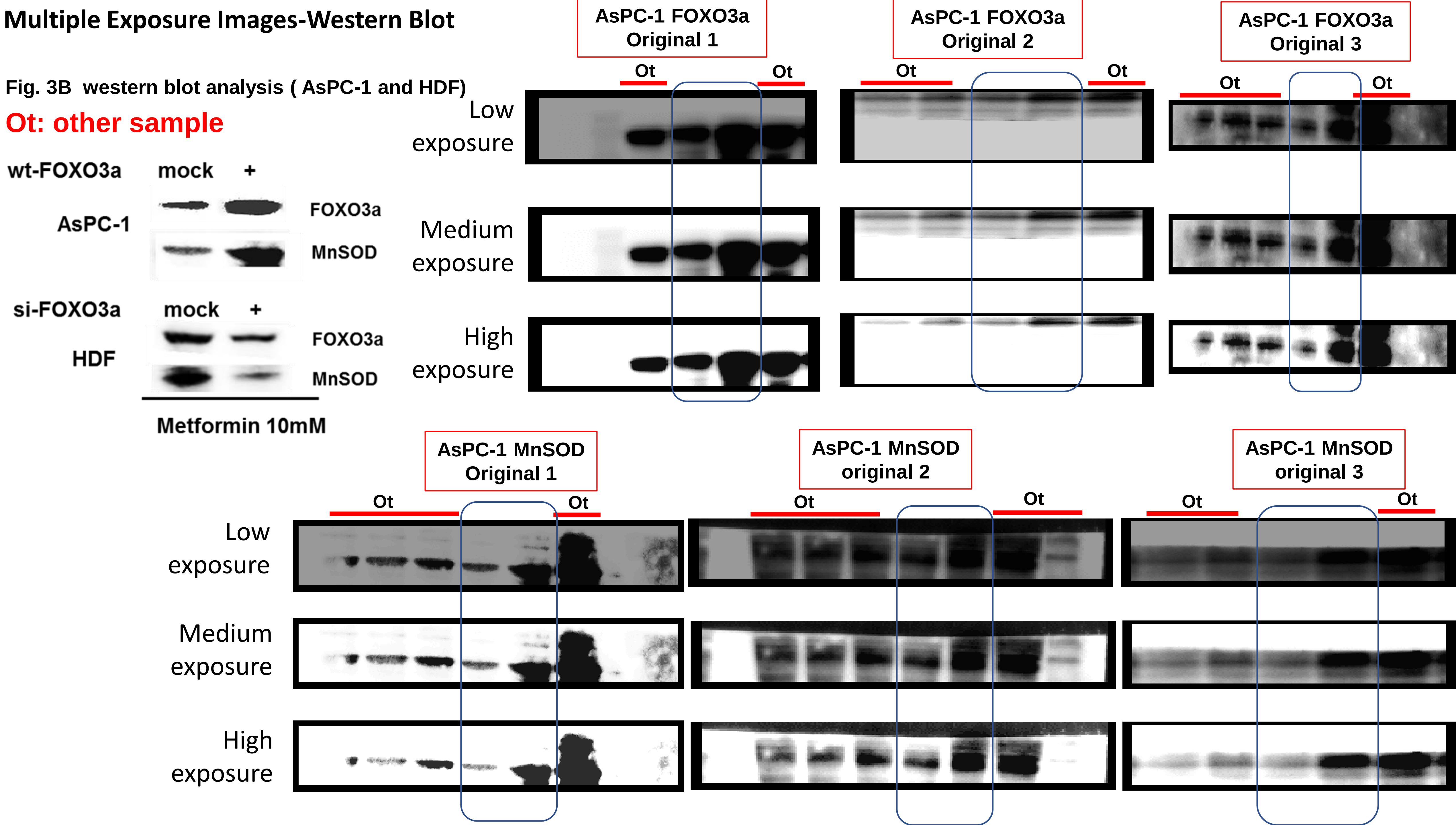

Multiple Exposure Images-Western Blot

Fig. 3B western blot analysis (AsPC-1 and HDF)

Ot: other sample

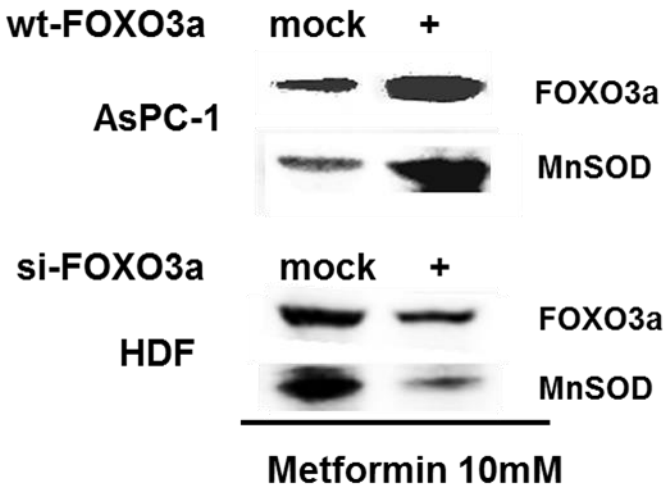

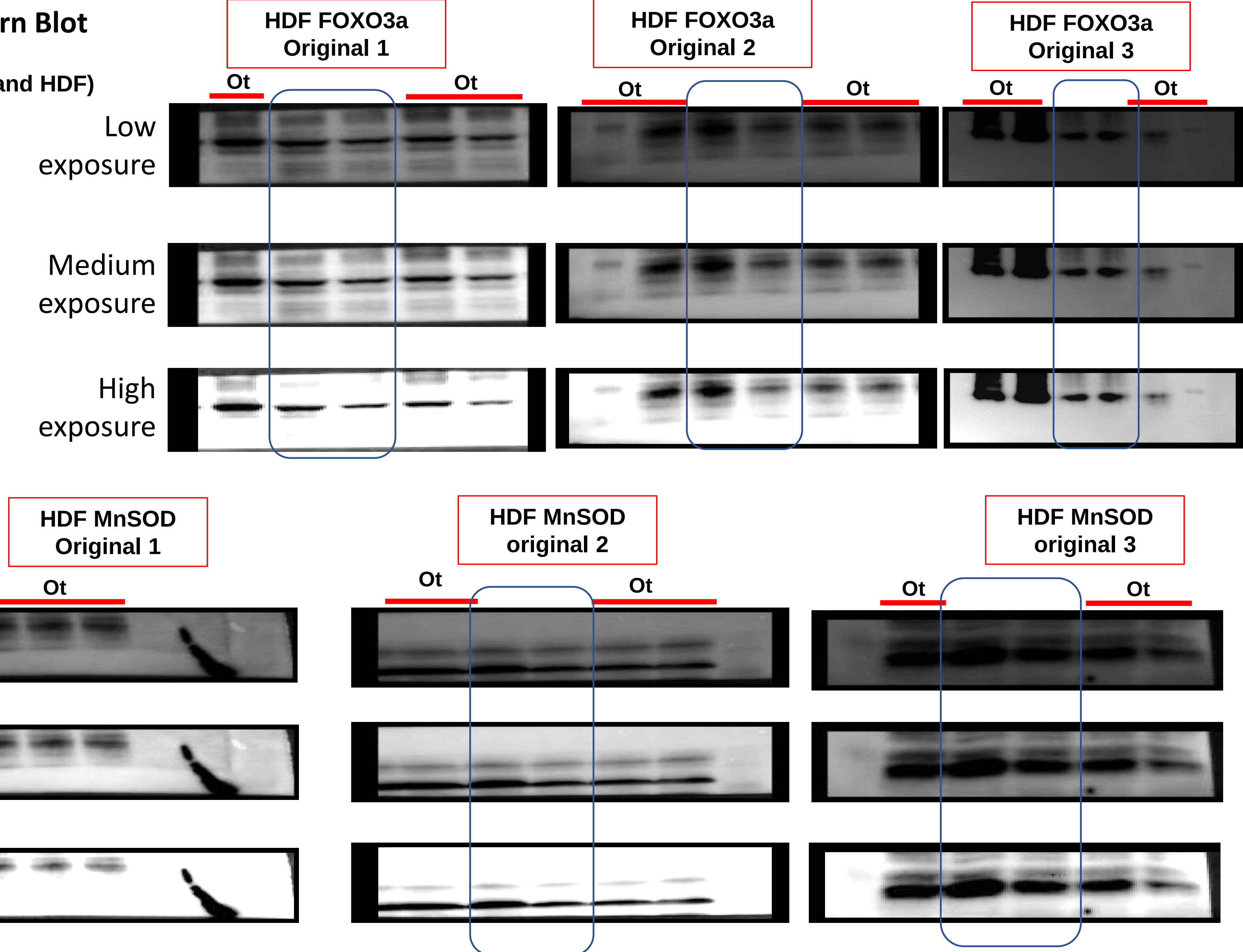

Multiple Exposure Images-Western Blot

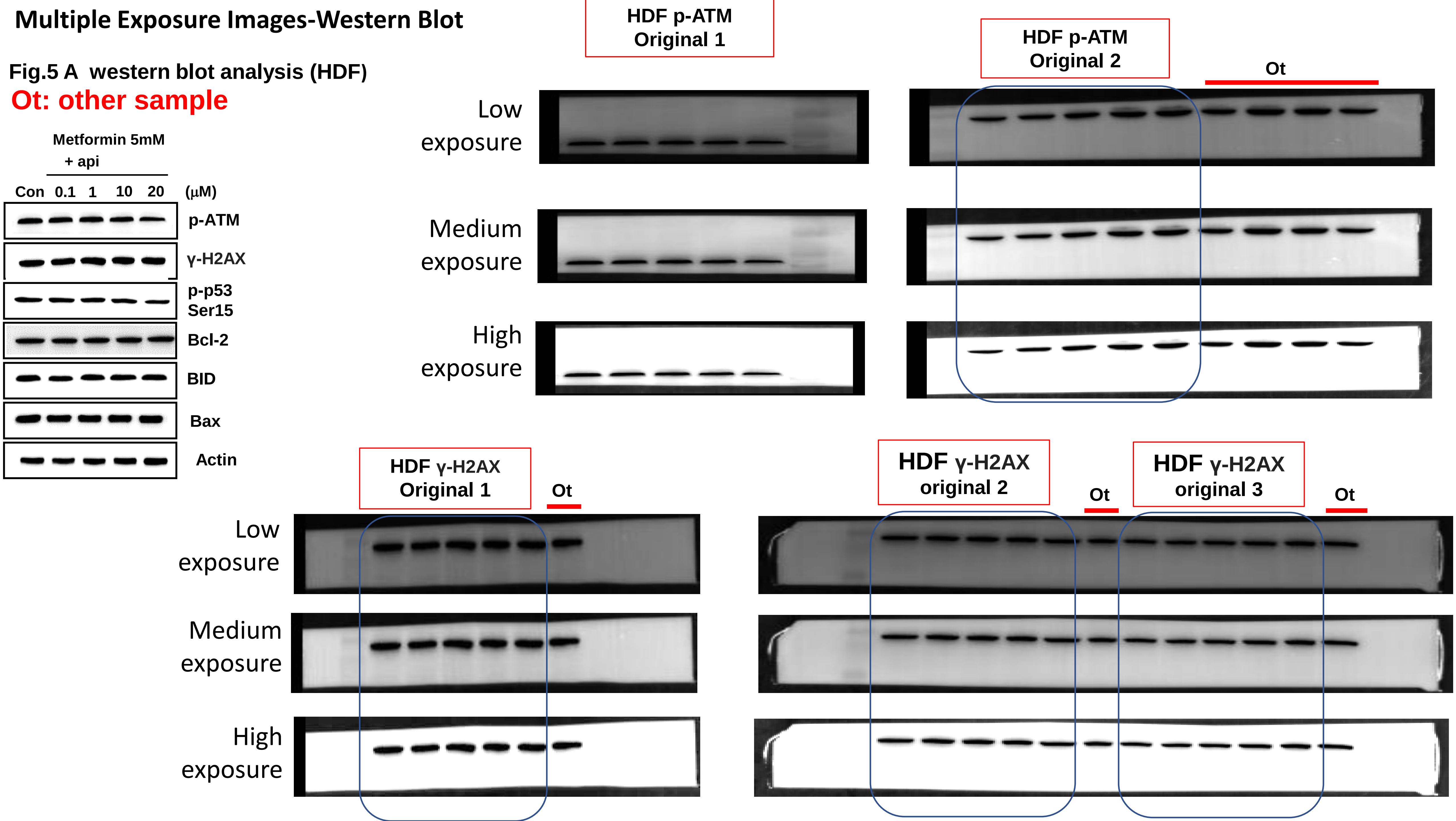

Multiple Exposure Images-Western Blot

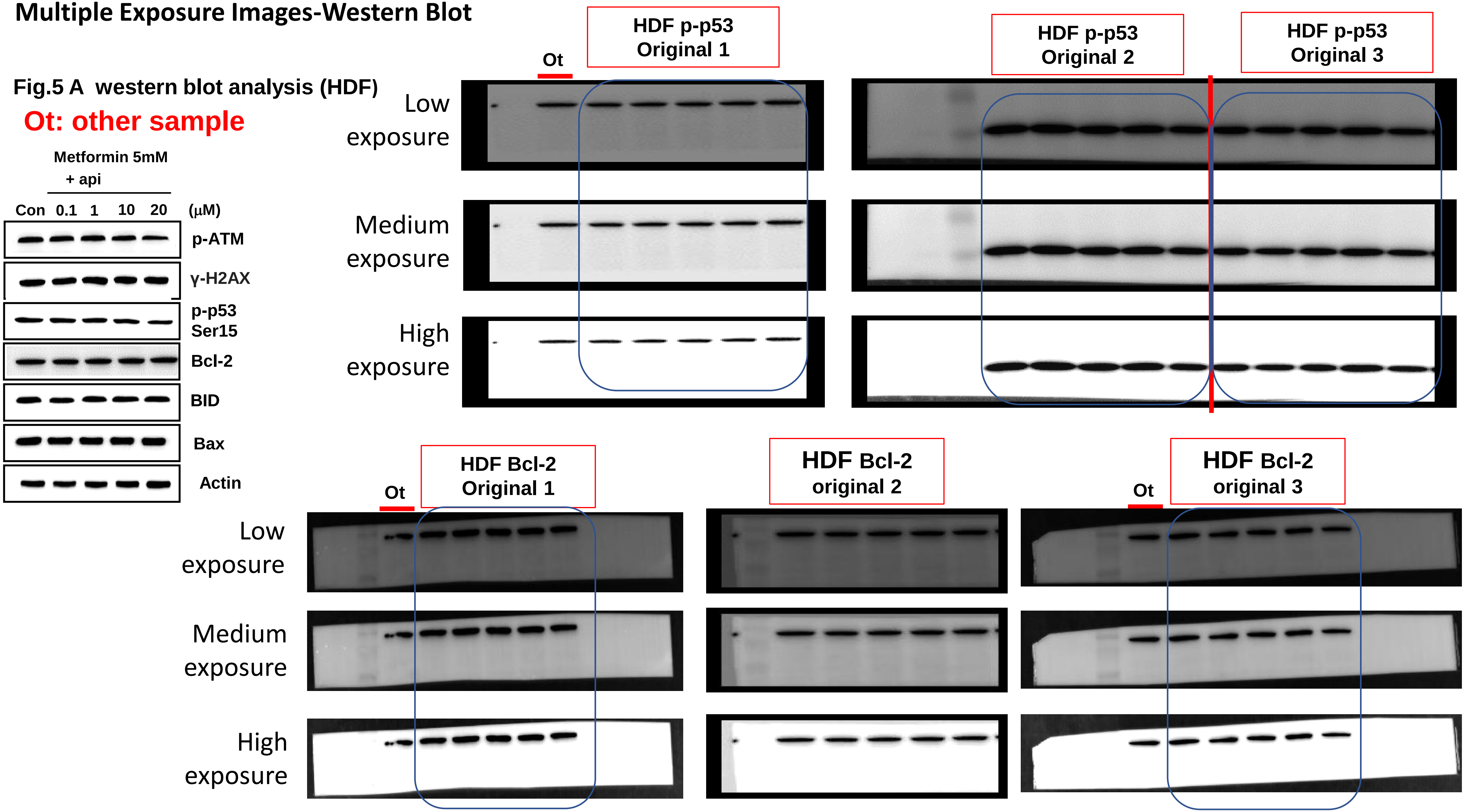

Multiple Exposure Images-Western Blot

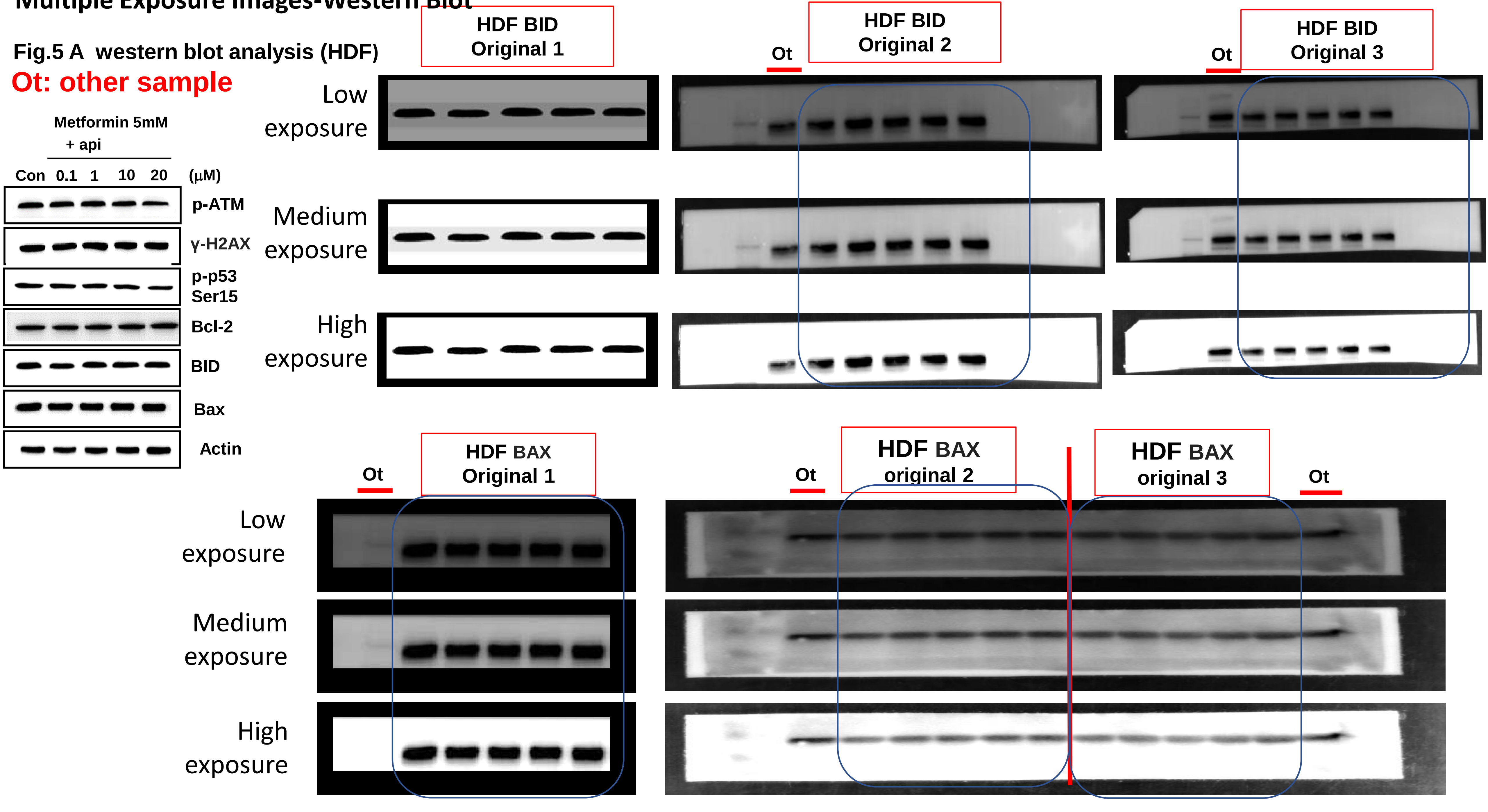

Multiple Exposure Images-Western Blot

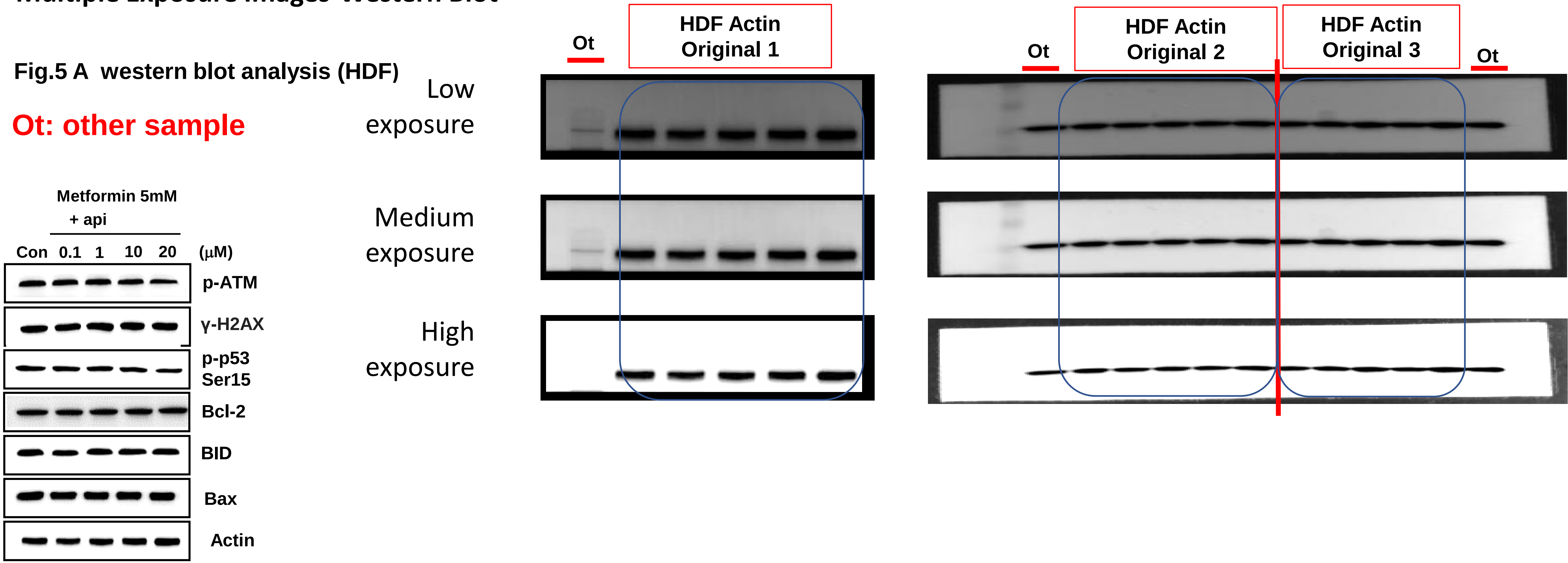

Multiple Exposure Images-Western Blot

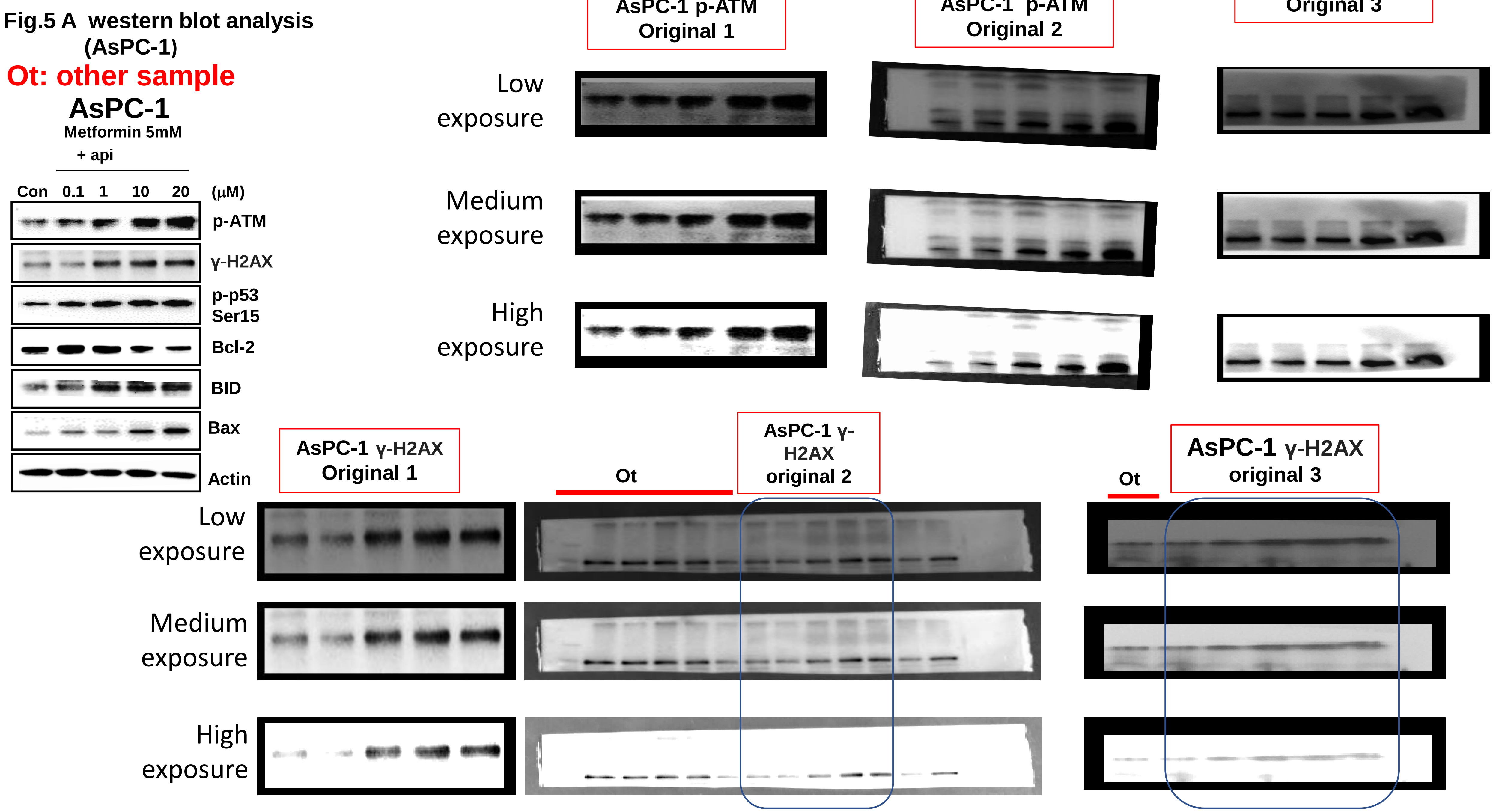

Multiple Exposure Images-Western Blot

Fig.5 A western blot analysis (AsPC-1)

Ot: other sample

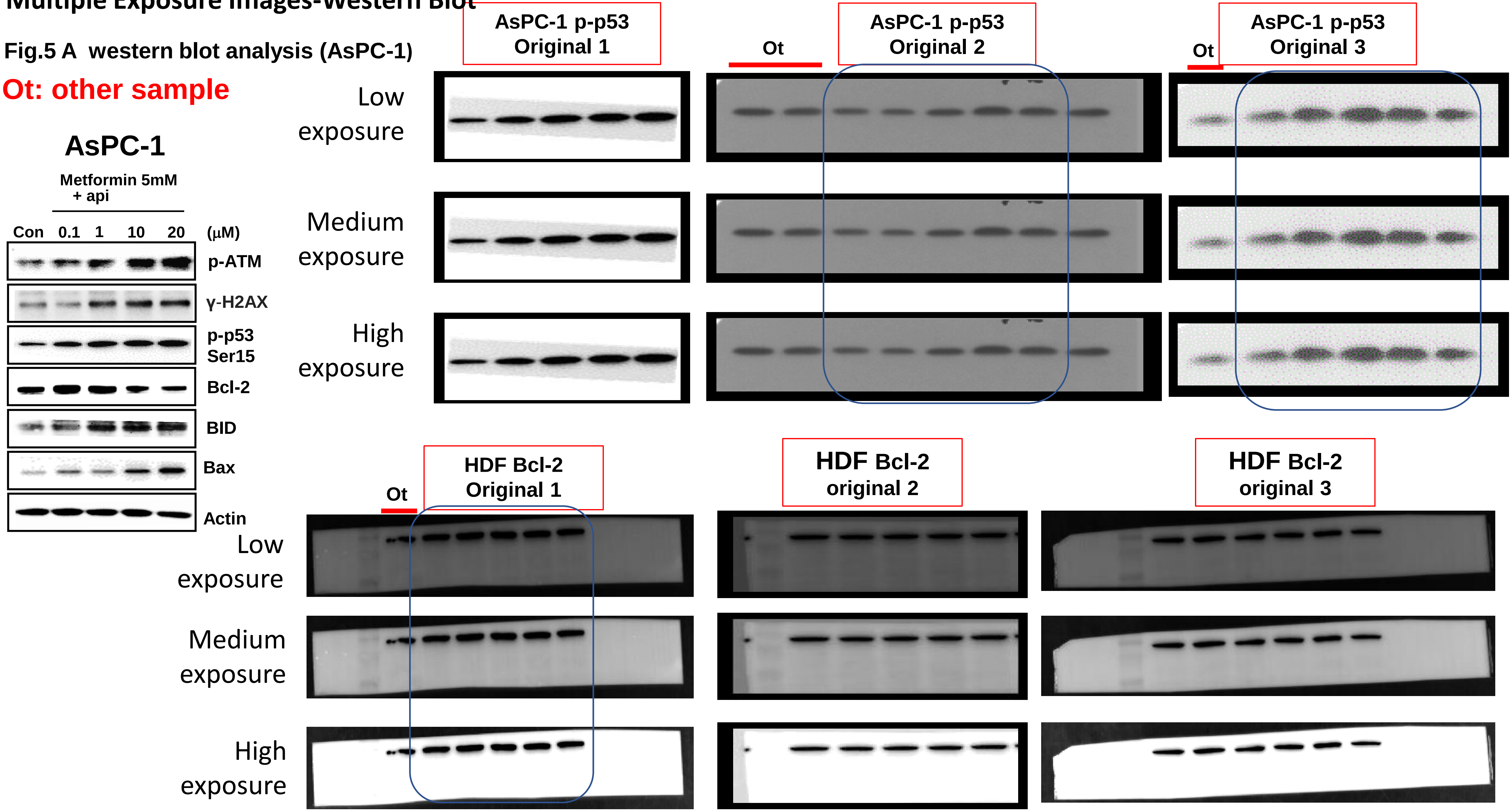

Multiple Exposure Images-Western Blot

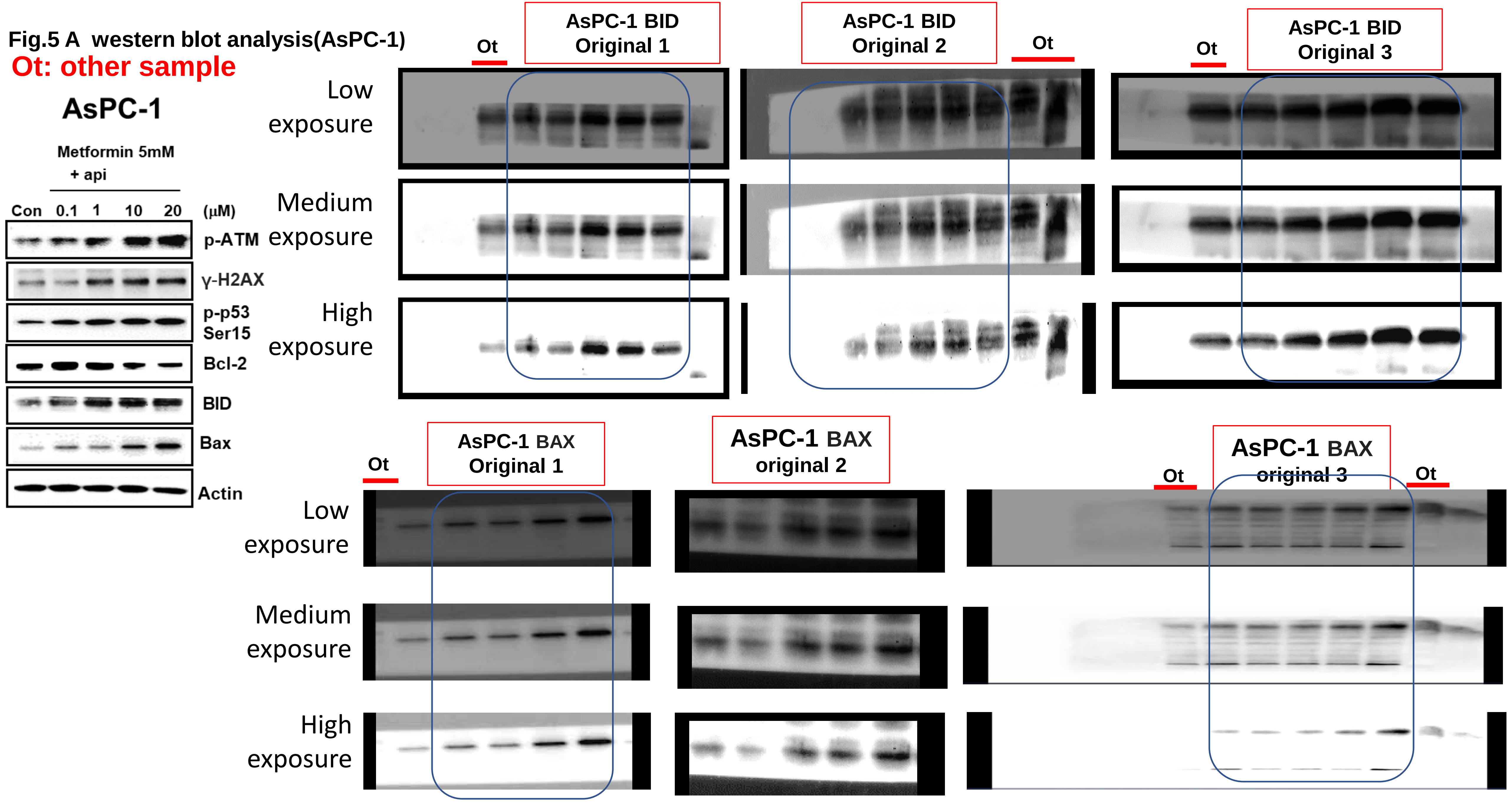

Multiple Exposure Images-Western Blot

Fig.5 A western blot analysis (AsPC-1)

Ot: other sample

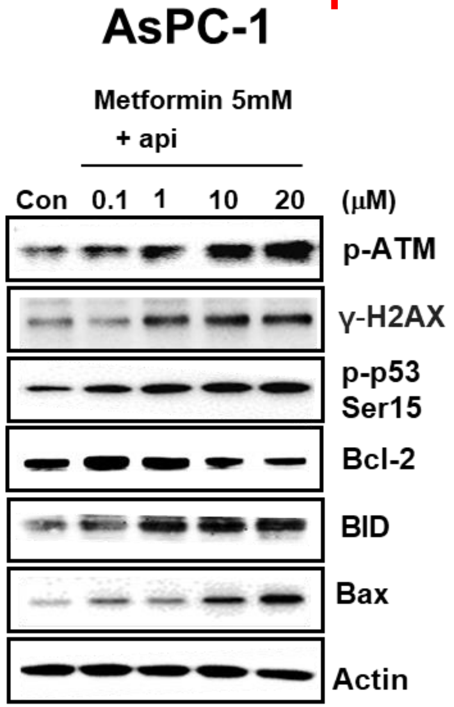

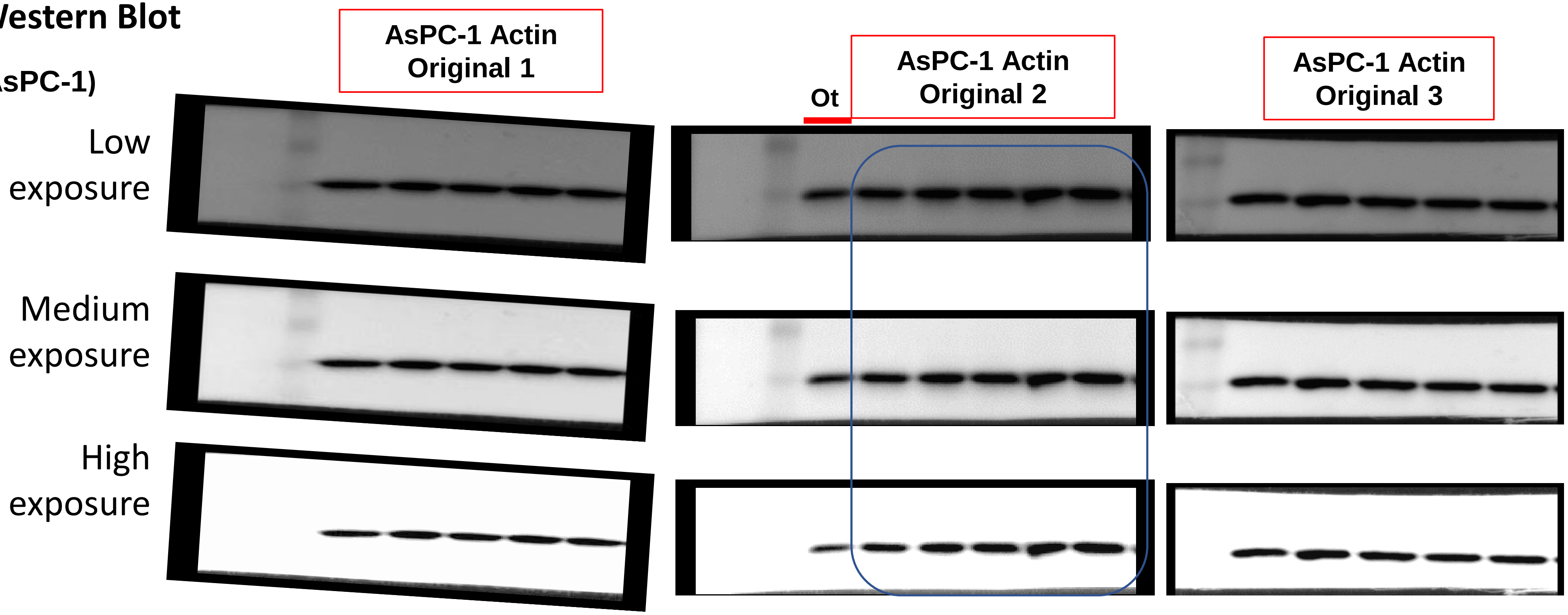

Multiple Exposure Images-Western Blot

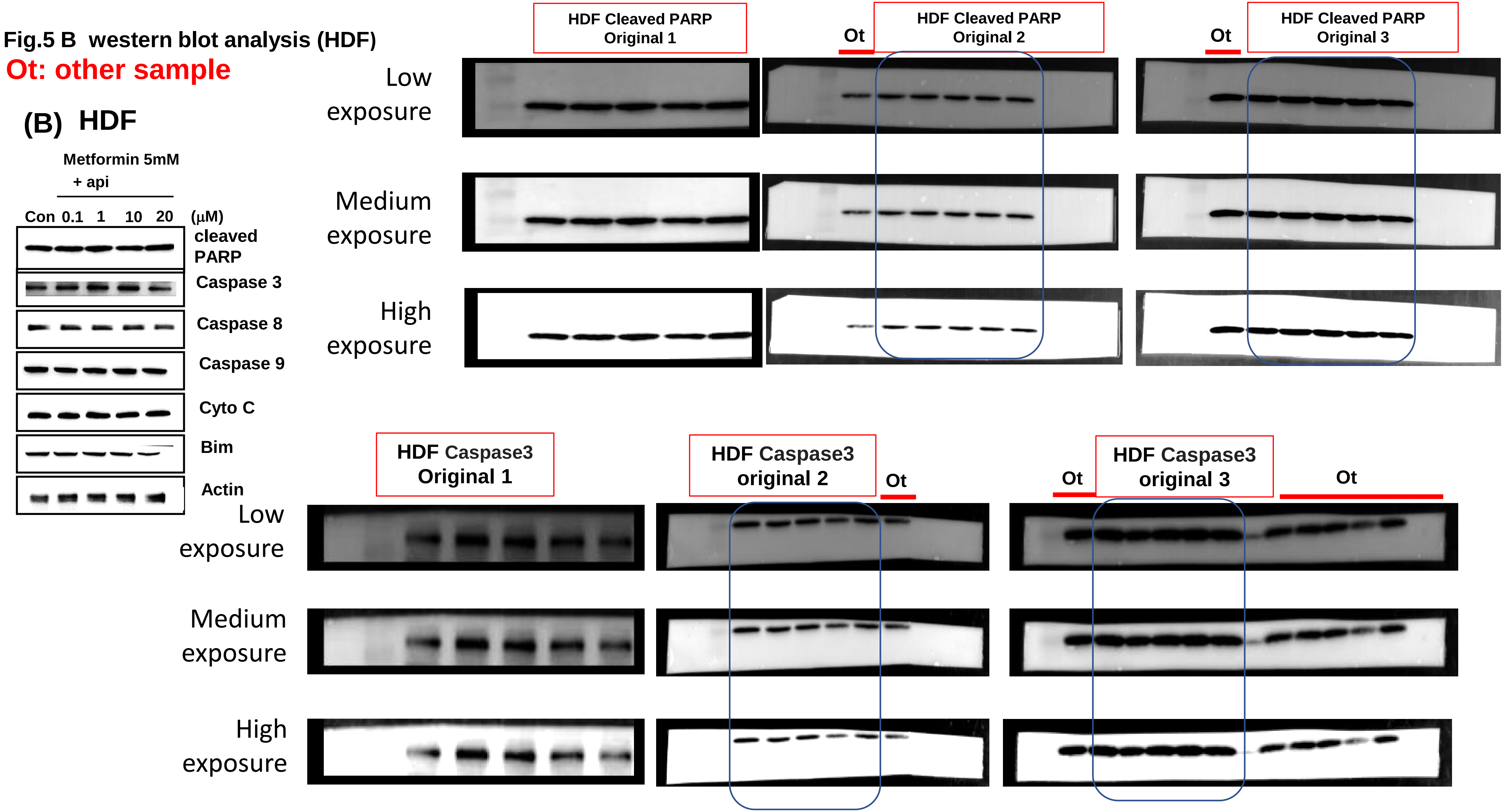

Multiple Exposure Images-Western Blot

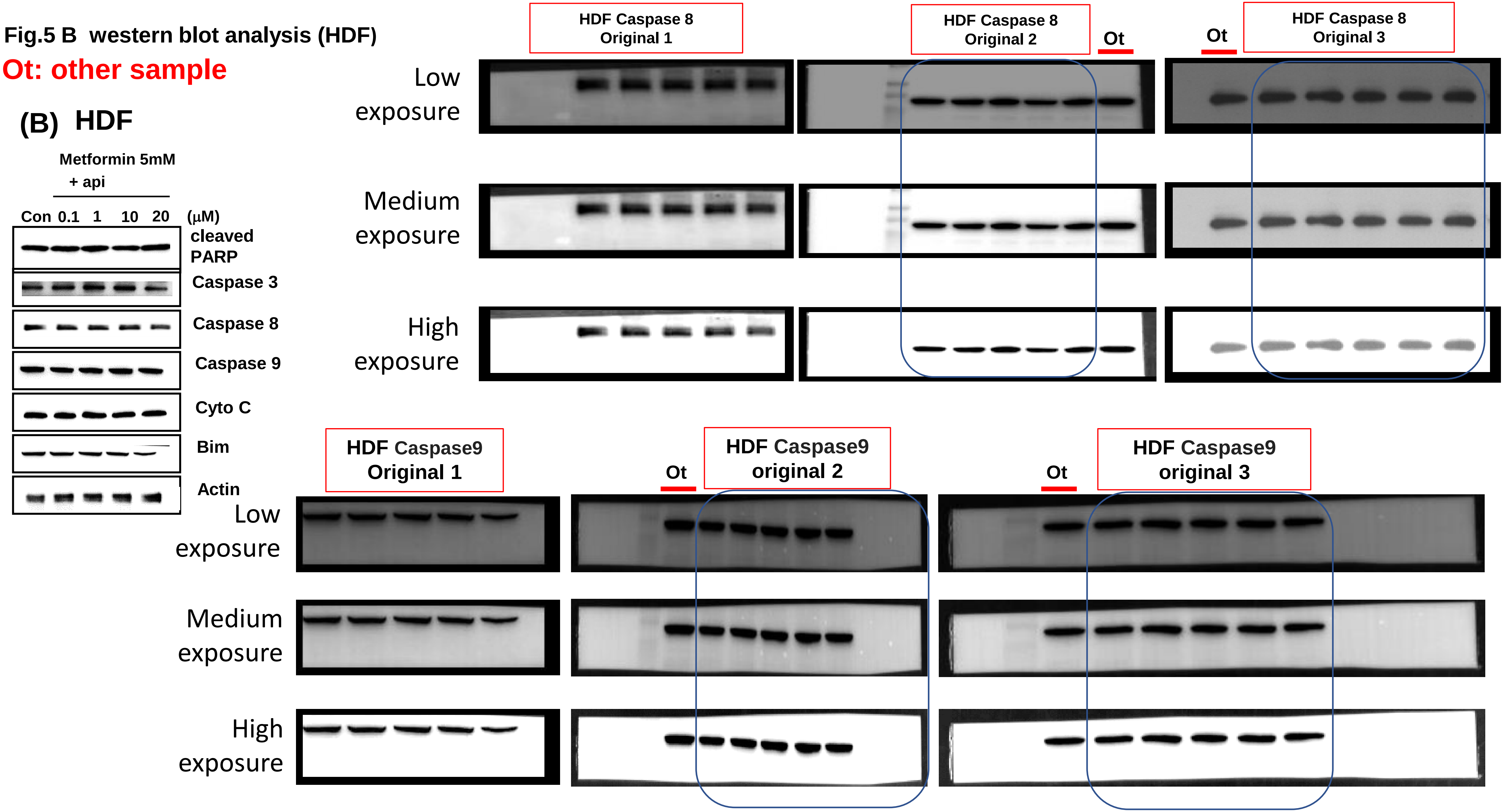

Multiple Exposure Images-Western Blot

Fig.5 B western blot analysis (HDF)

Ot: other sample

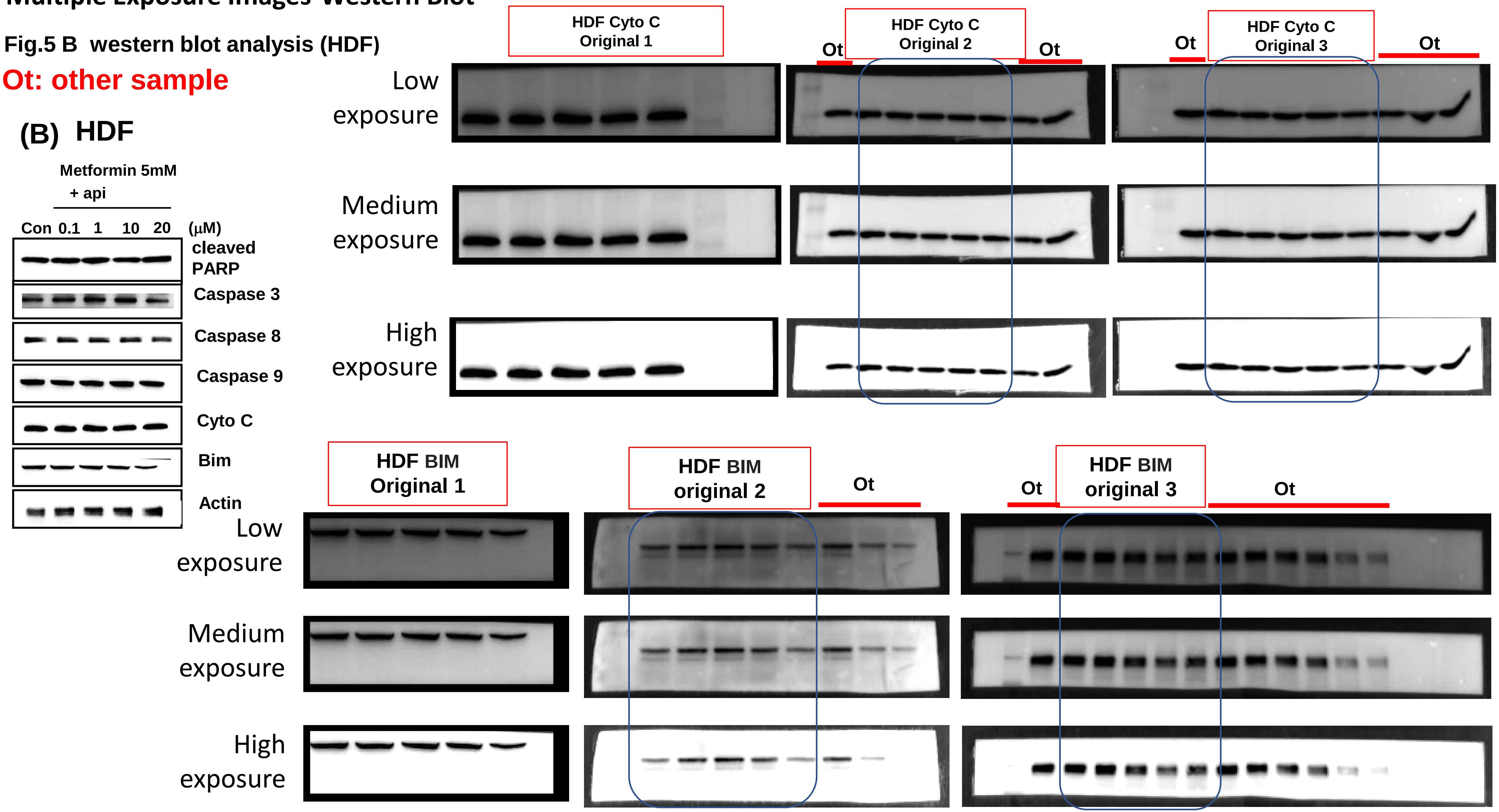

Multiple Exposure Images-Western Blot

Fig.5 B western blot analysis (HDF)

Ot: other sample

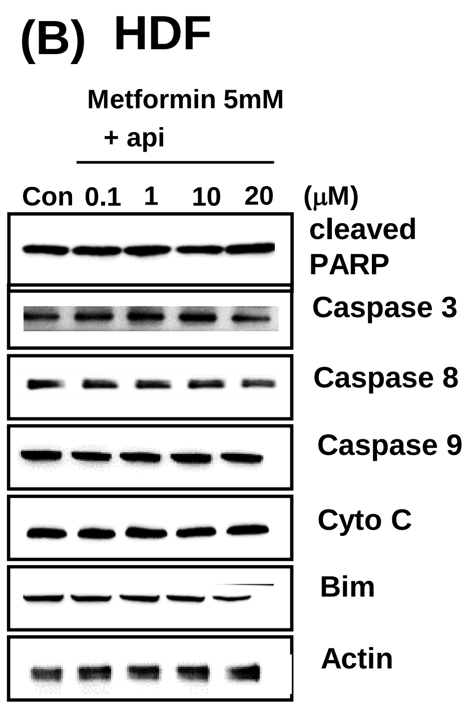

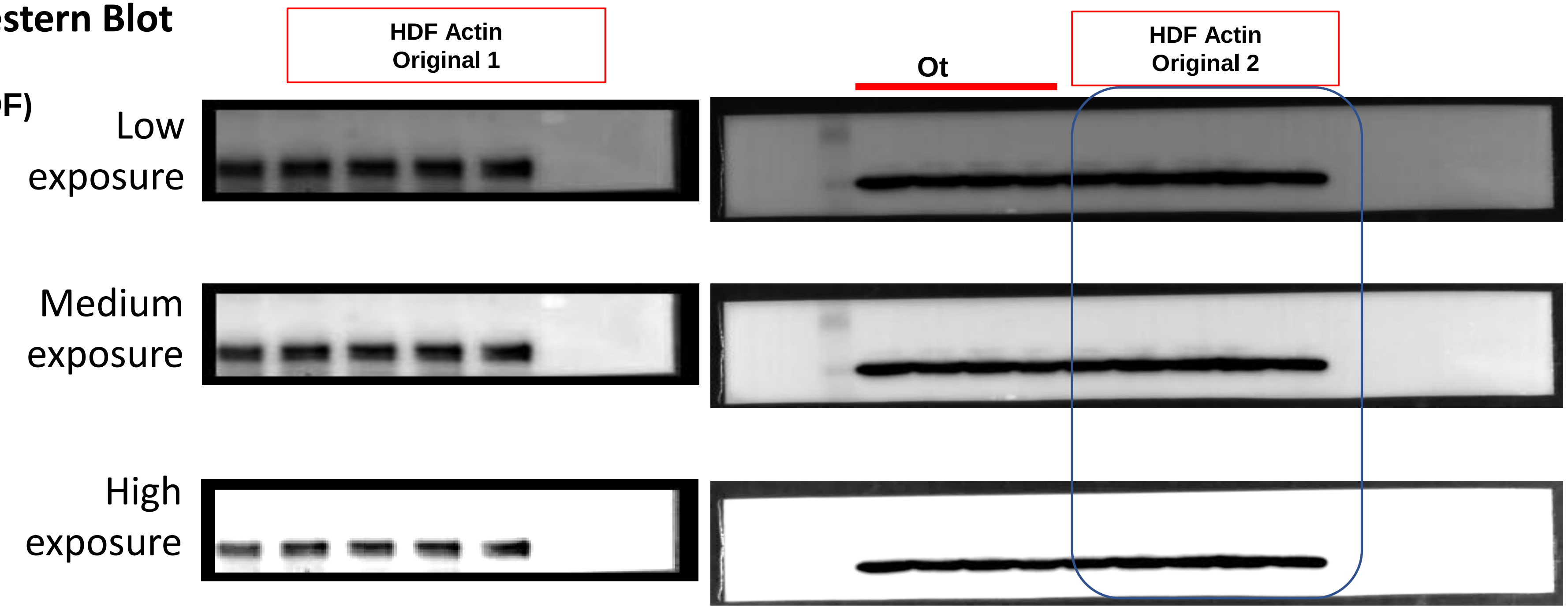

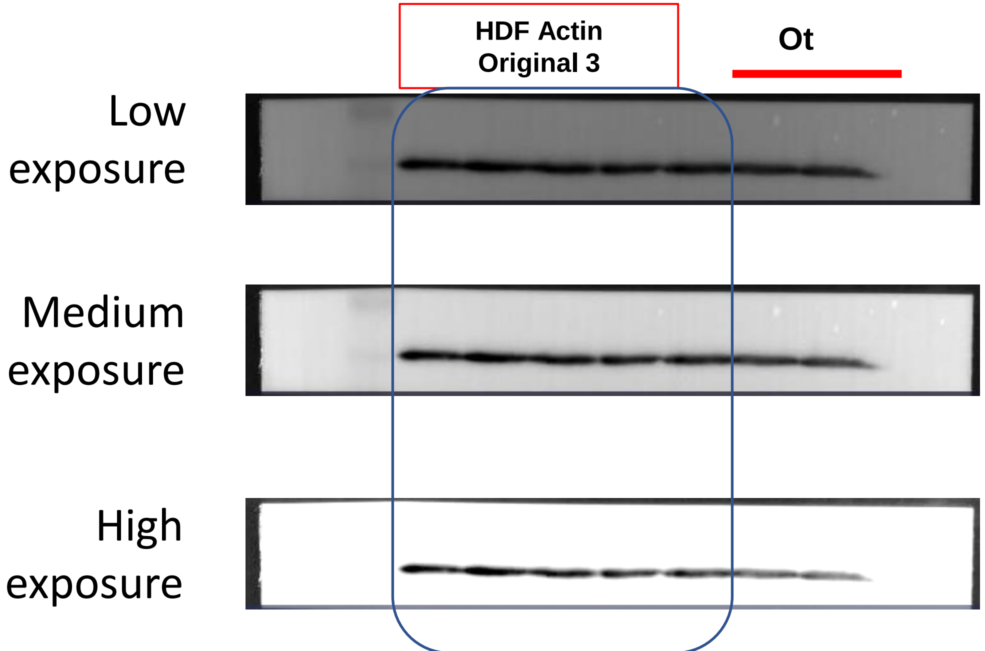

Multiple Exposure Images-Western Blot

Fig.5 B western blot analysis (AsPC-1)

Ot: other sample

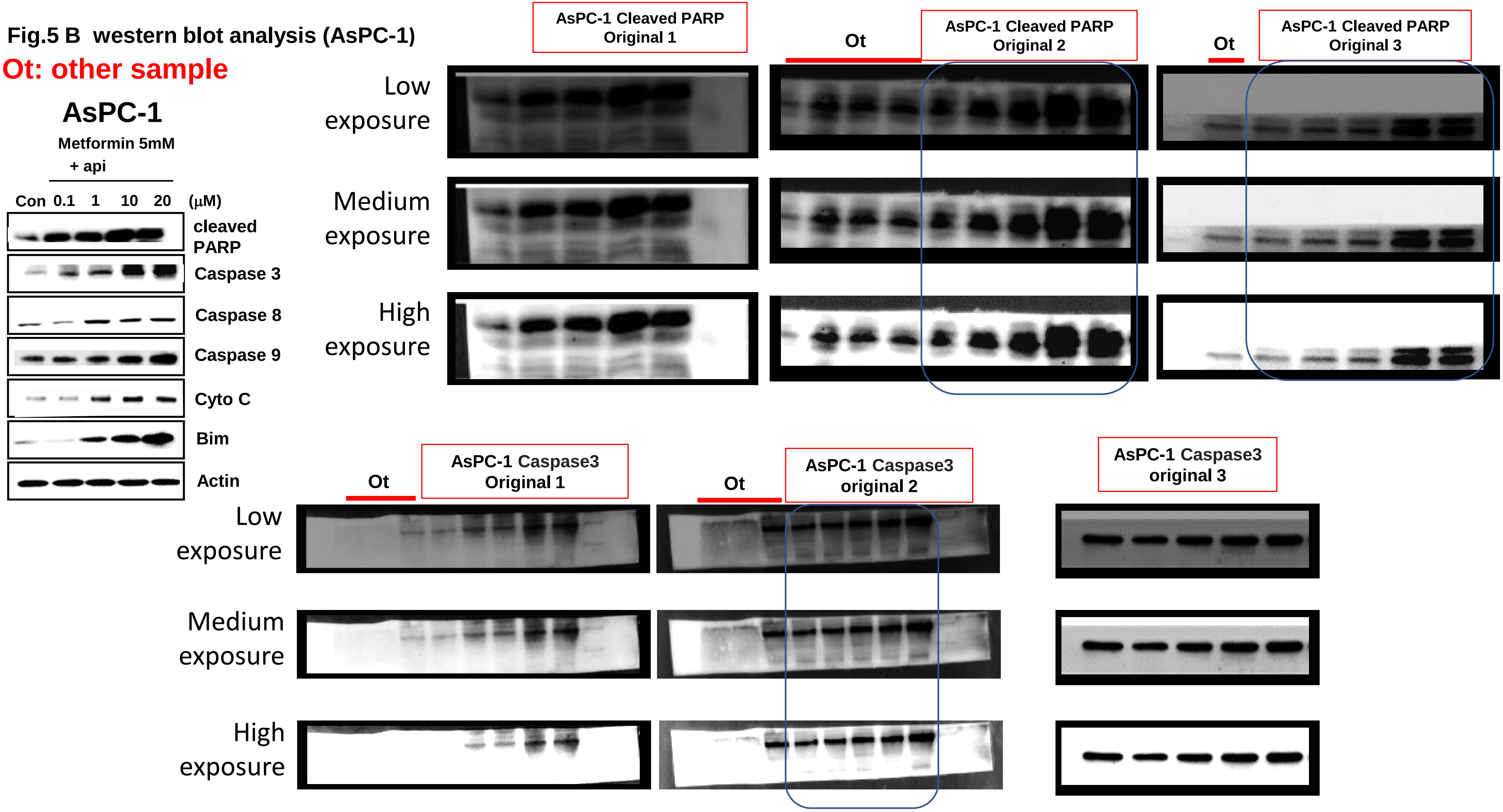

Multiple Exposure Images-Western Blot

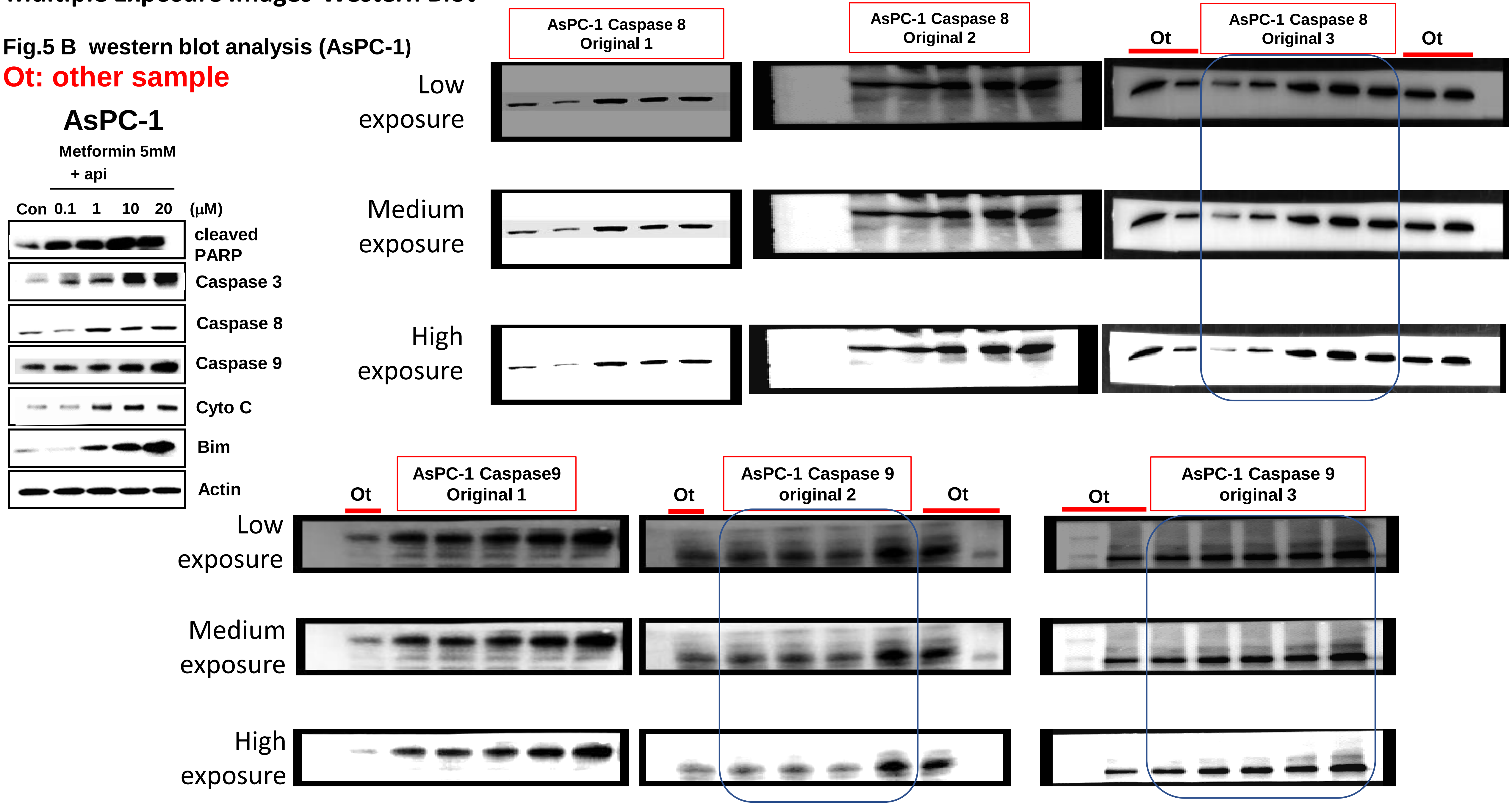

Multiple Exposure Images-Western Blot

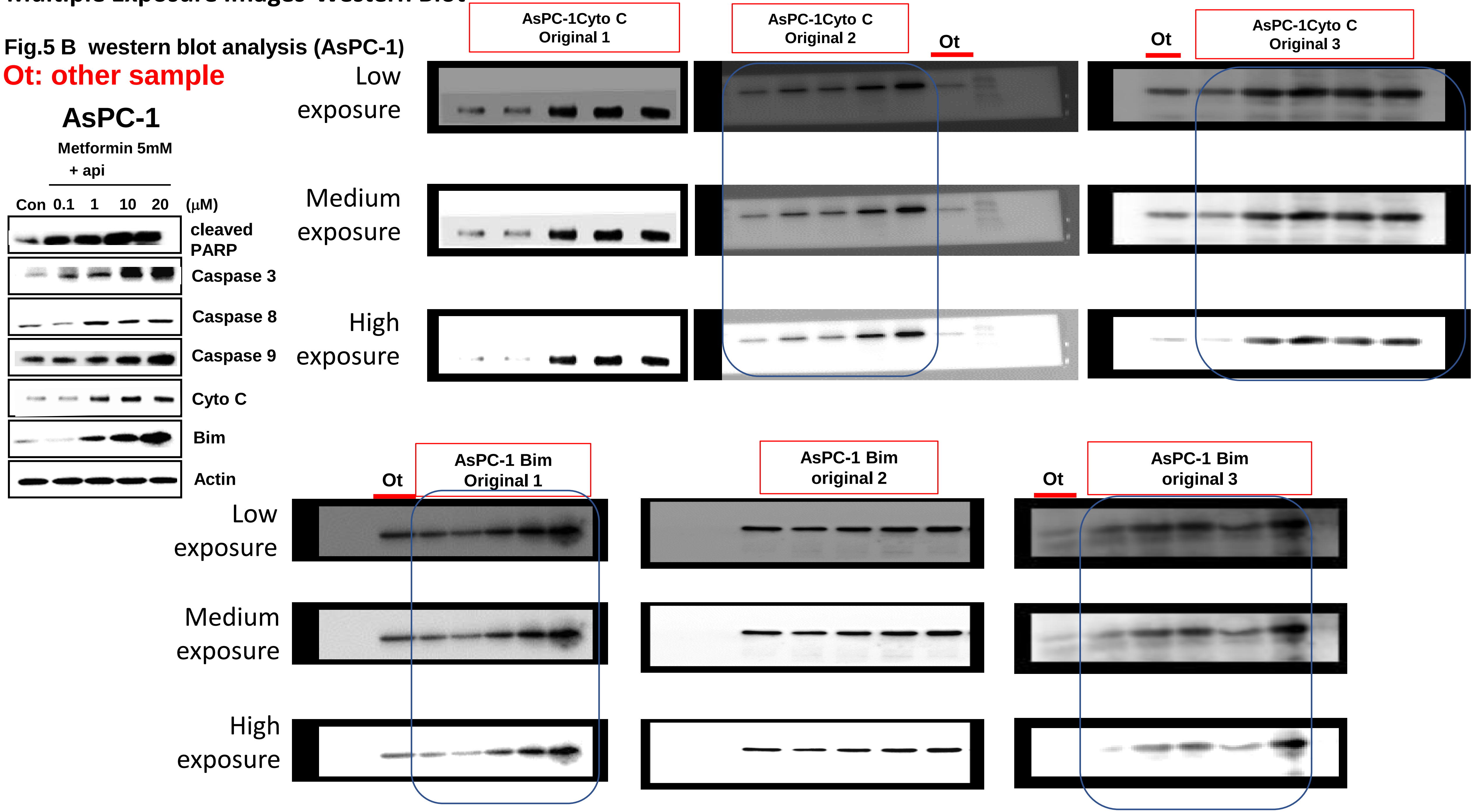

Multiple Exposure Images-Western Blot

Fig.5 B western blot analysis (AsPC-1)

Ot: other sample

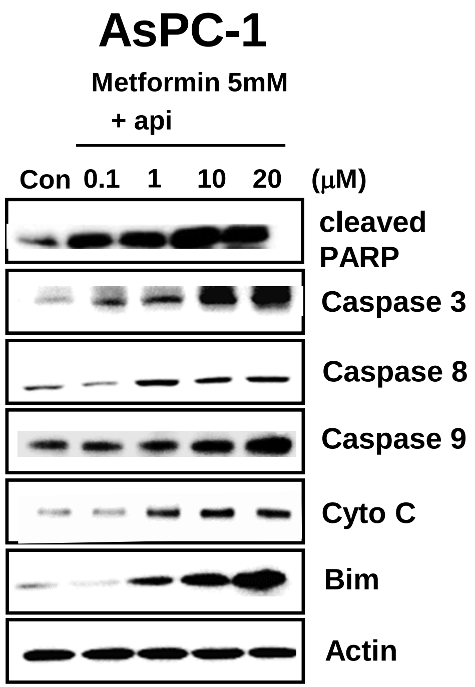

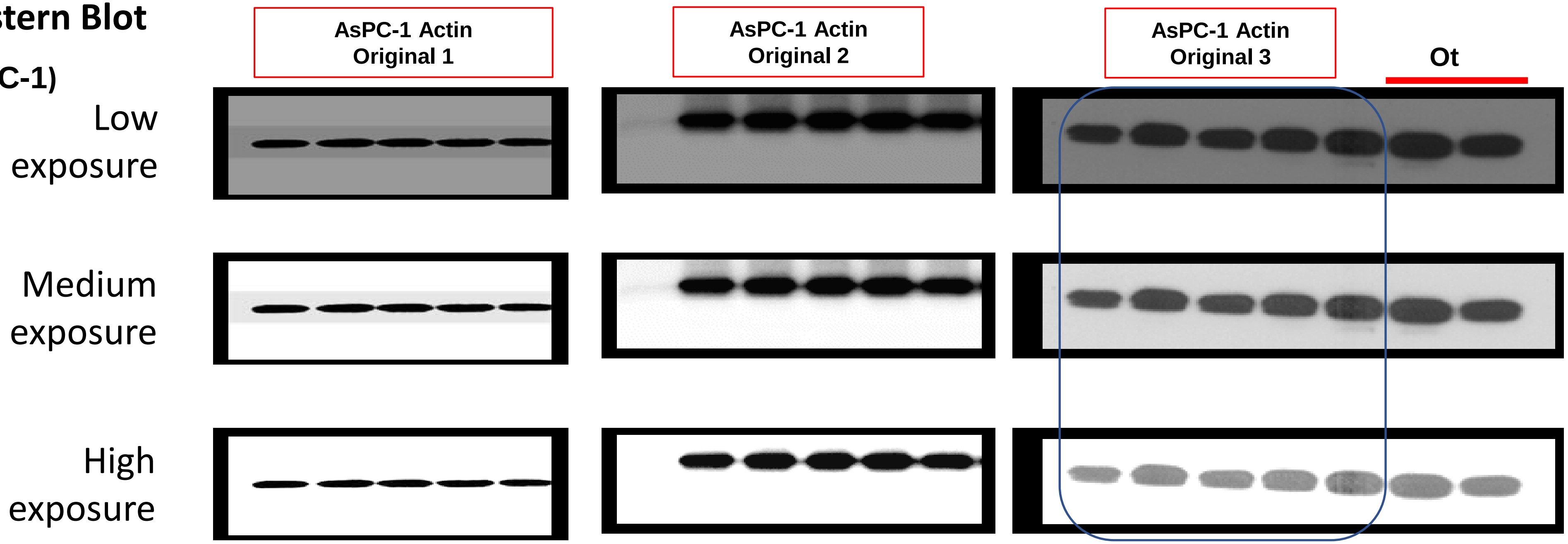

Multiple Exposure Images-Western Blot

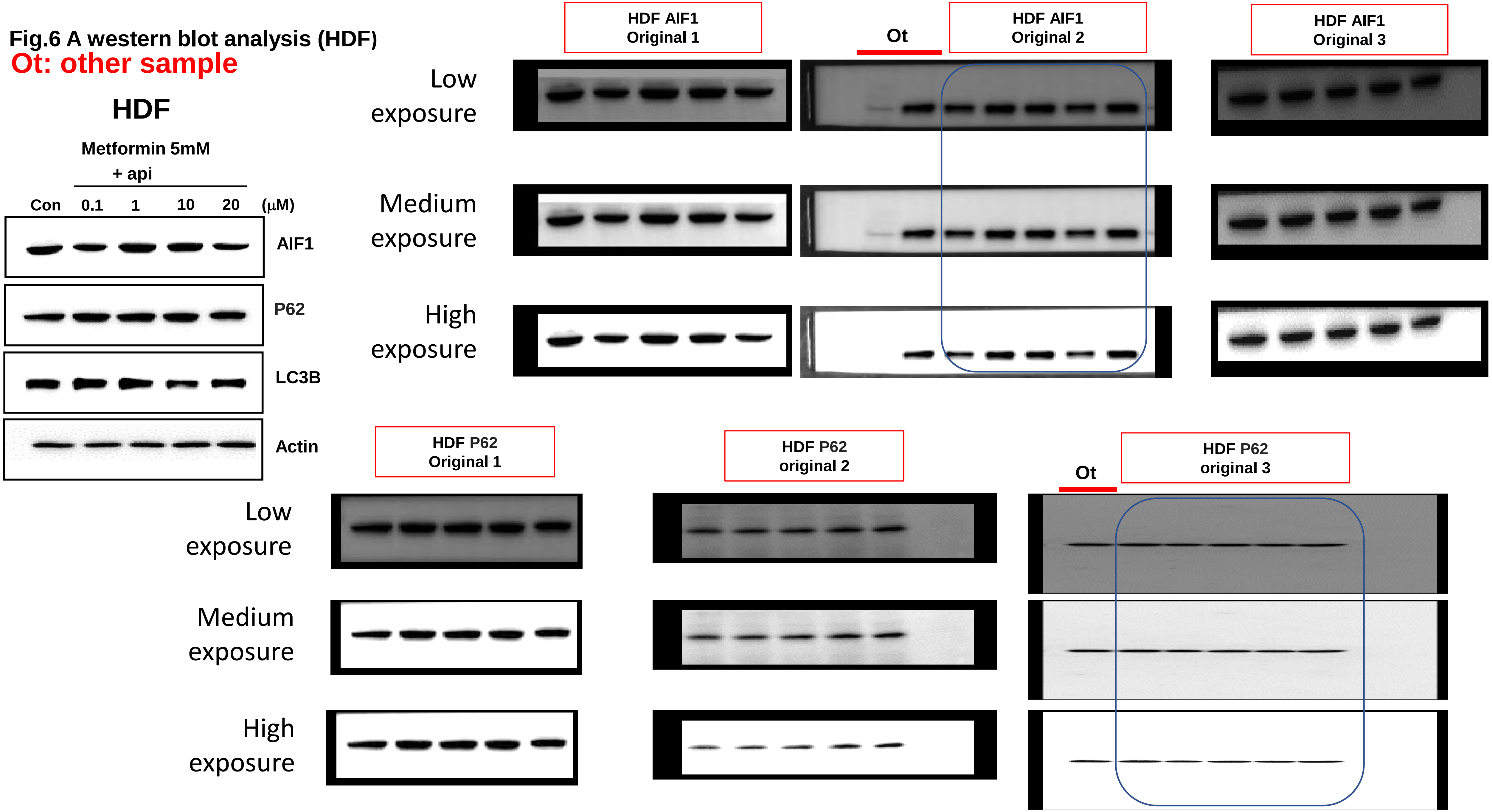

Multiple Exposure Images-Western Blot

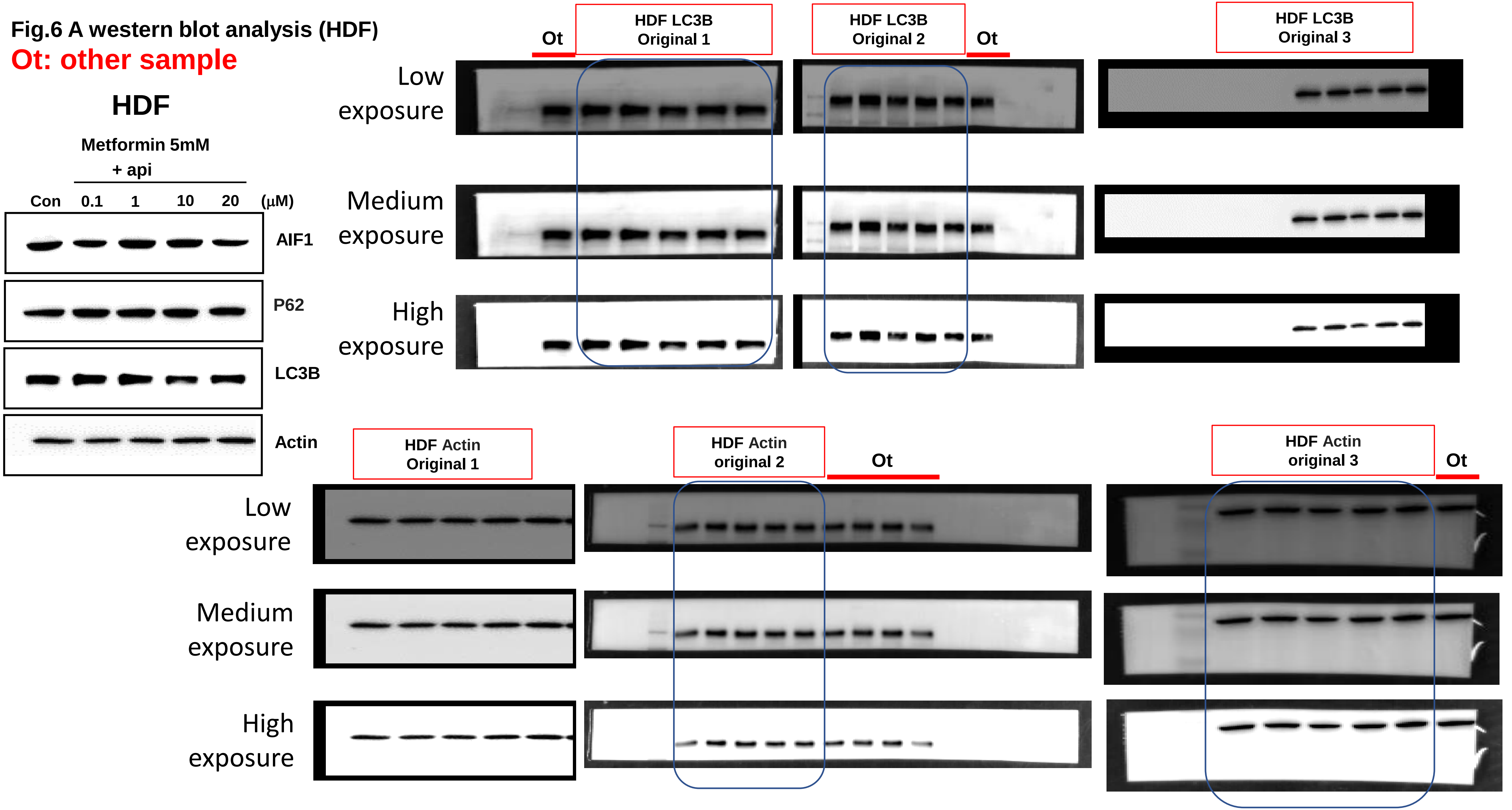

Multiple Exposure Images-Western Blot

Fig.6 A western blot analysis (AsPC-1)

Ot: other sample

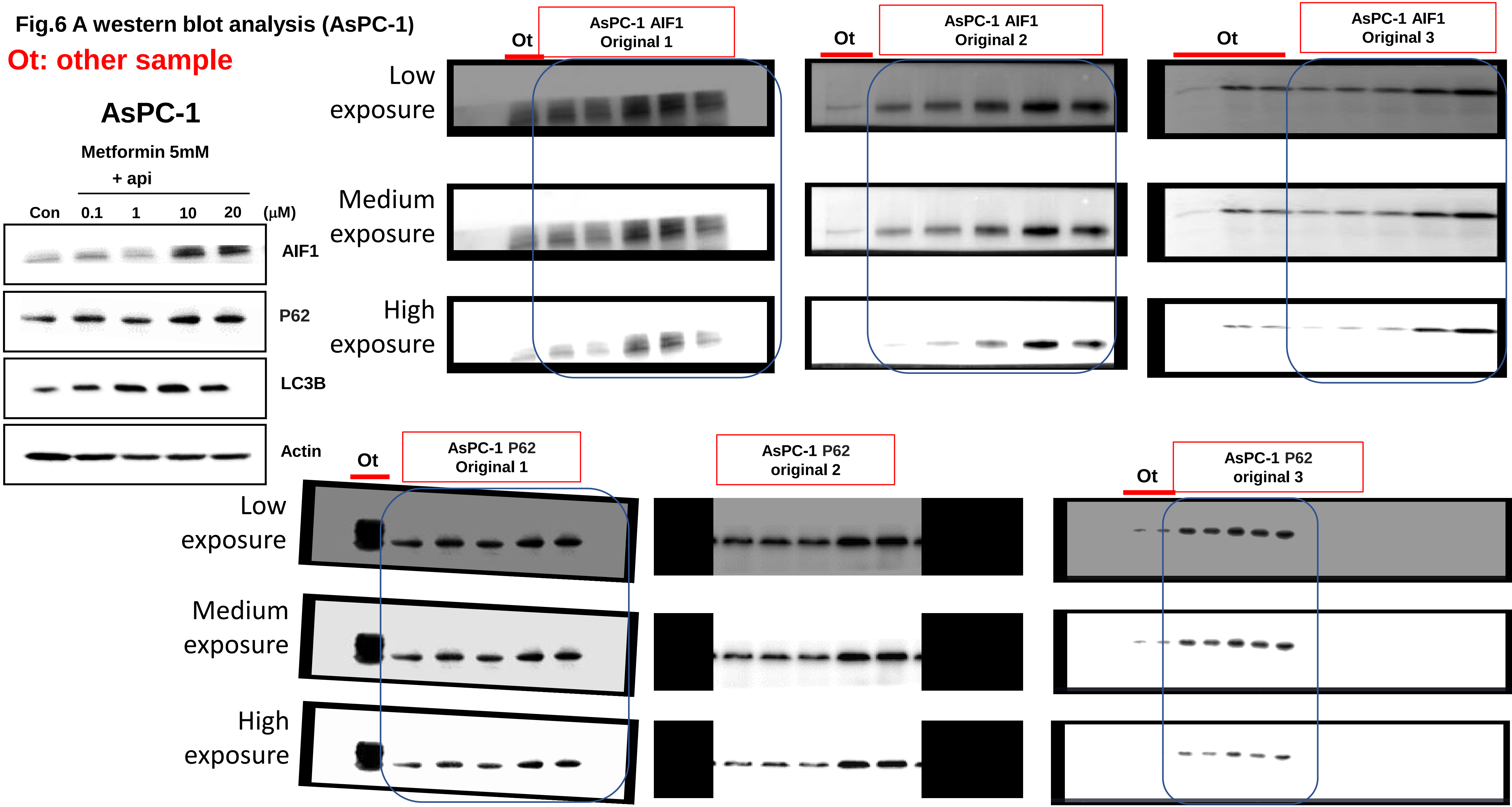

Multiple Exposure Images-Western Blot

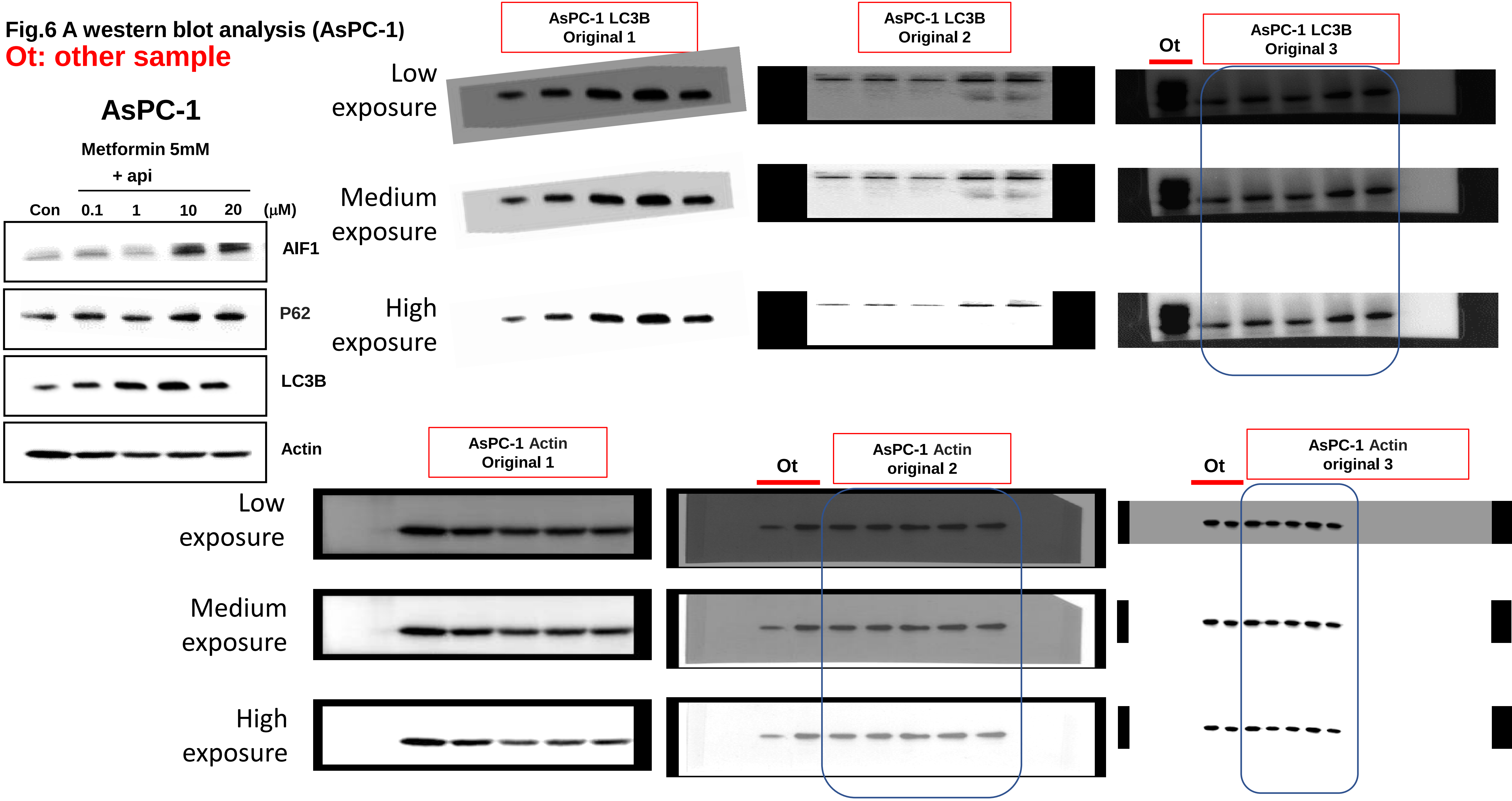

Multiple Exposure Images-Western Blot

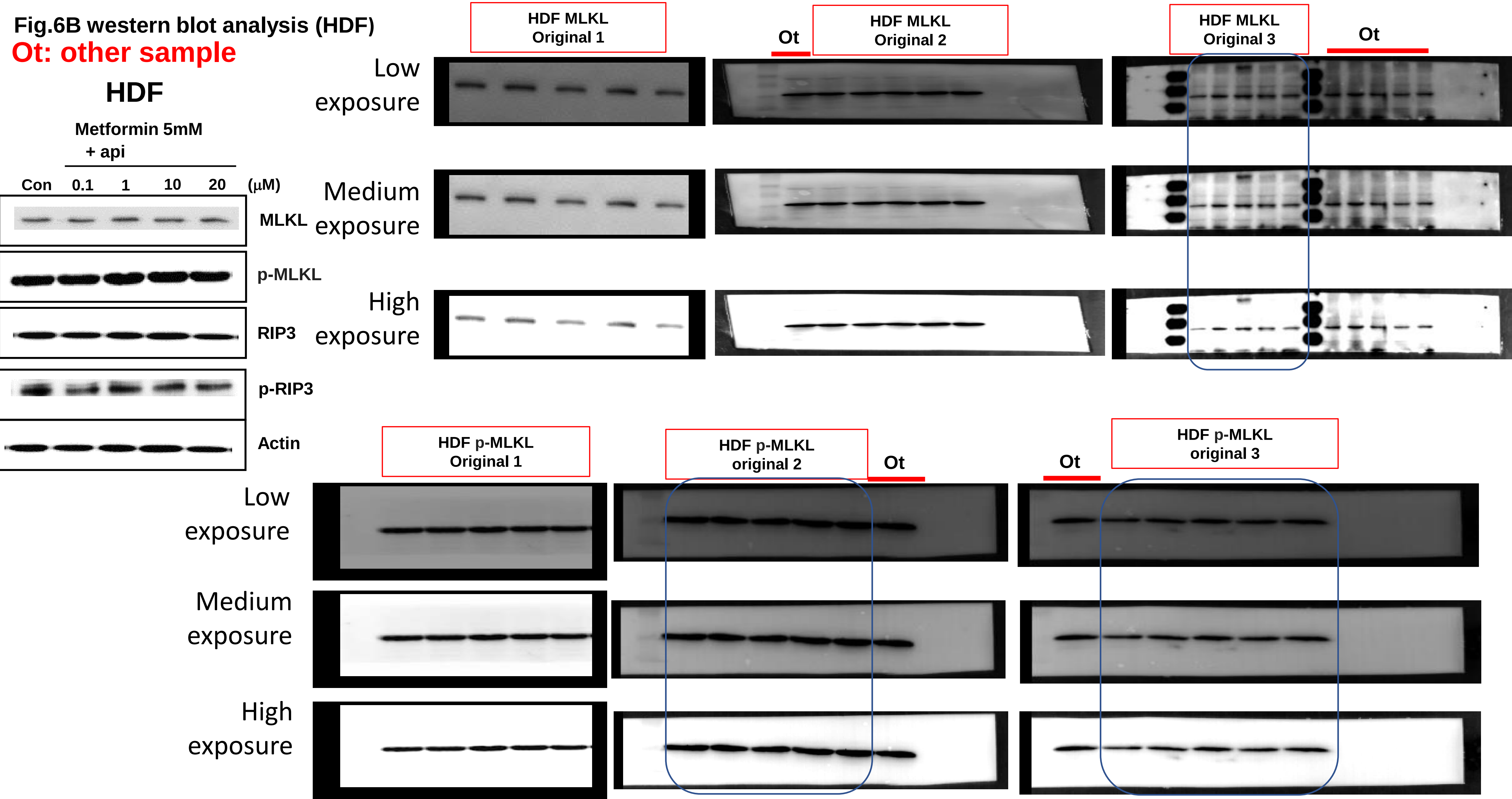

Multiple Exposure Images-Western Blot

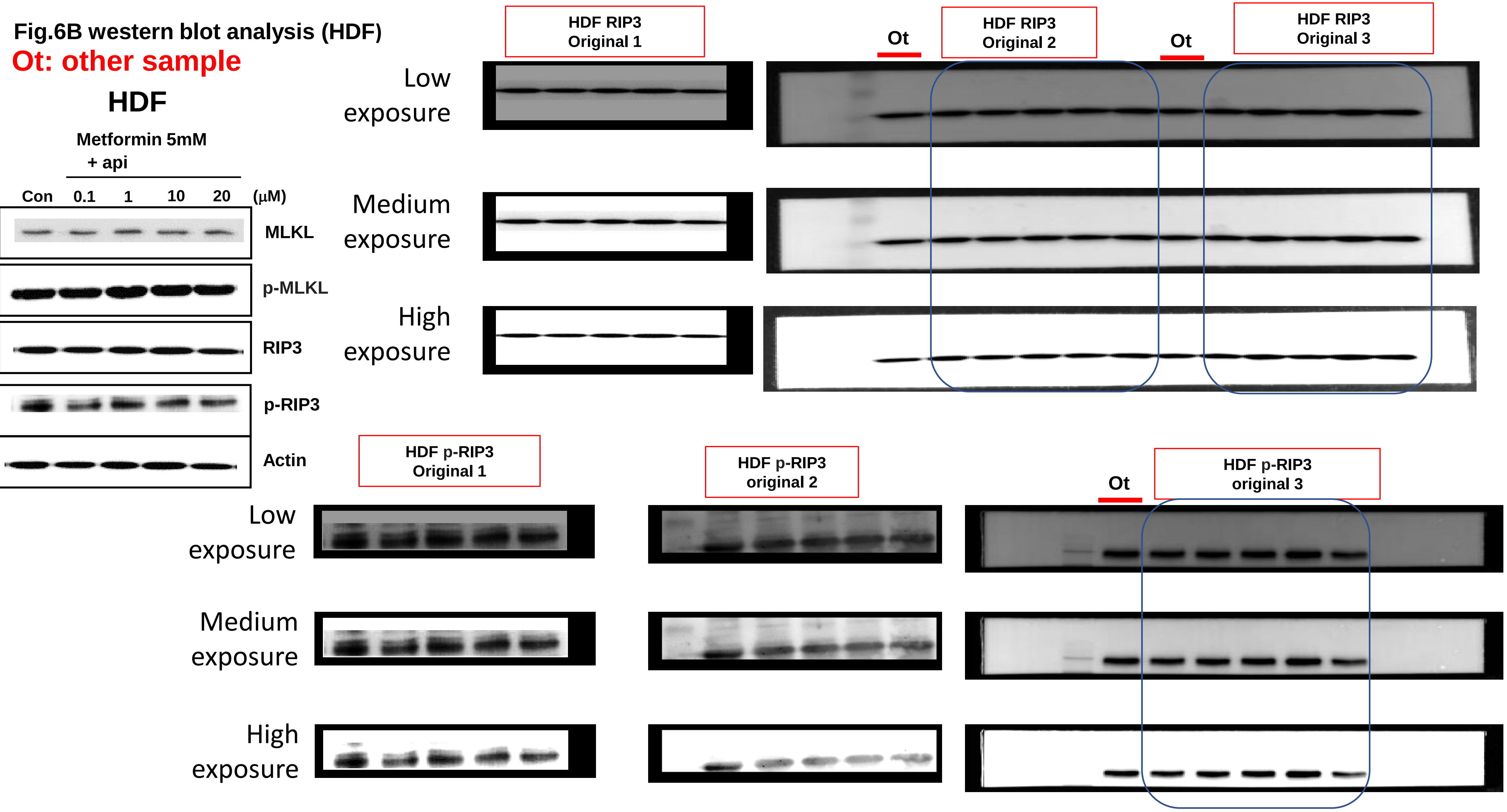

Multiple Exposure Images-Western Blot

Fig.6 B western blot analysis (HDF)

Ot: other sample

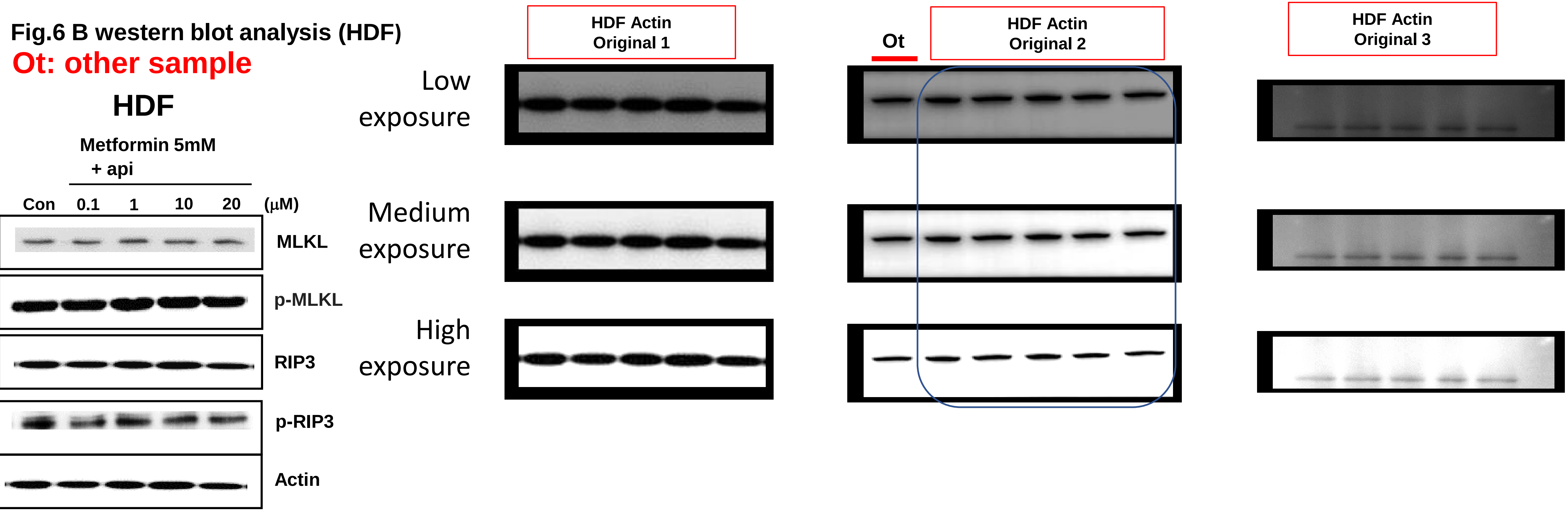

Multiple Exposure Images-Western Blot

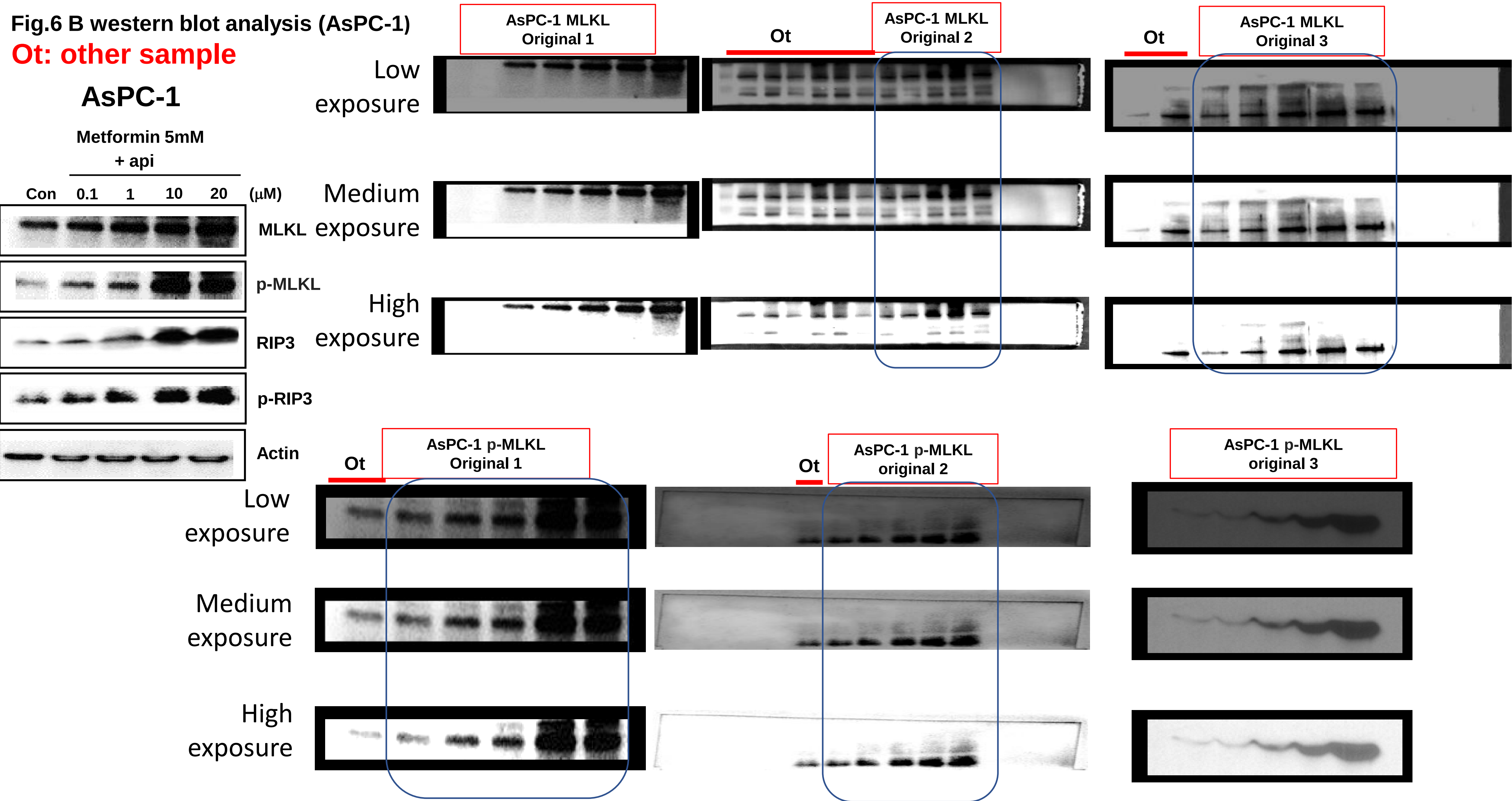

Multiple Exposure Images-Western Blot

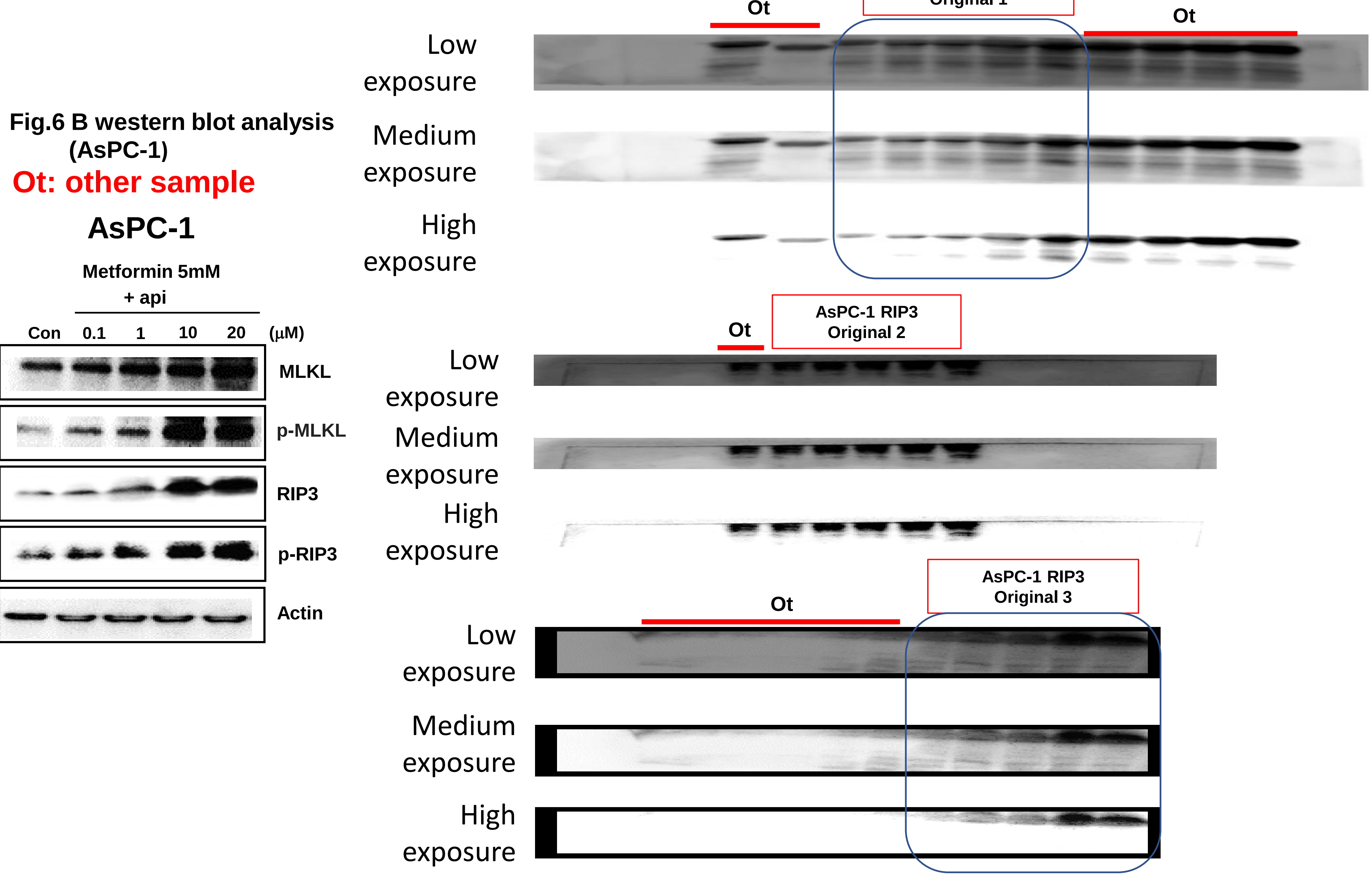

Multiple Exposure Images-Western Blot

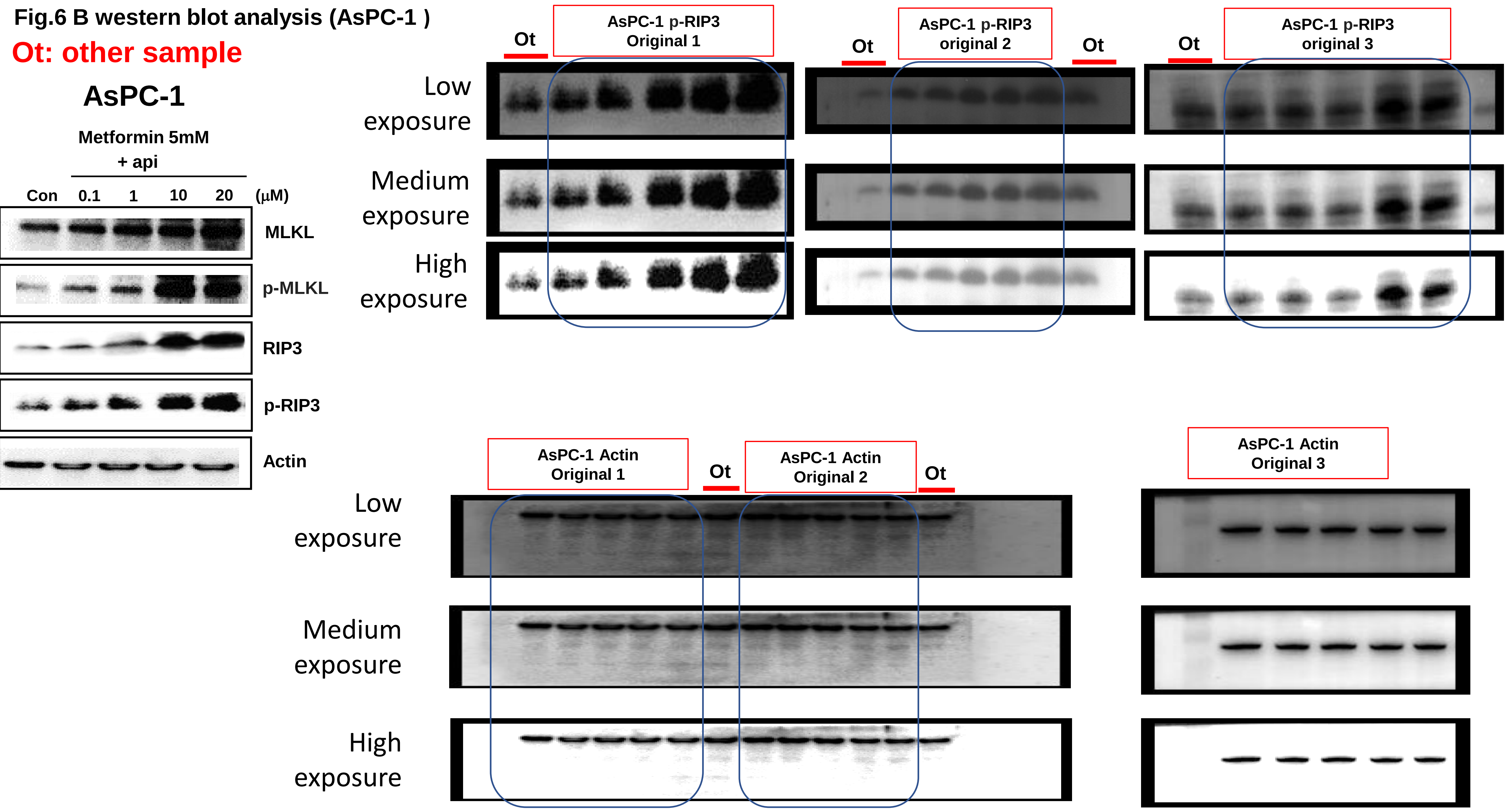

Fig.6 B western blot analysis (AsPC-1)

Ot: other sample
